# Supplementary material for: Unraveling and controlling late-onset neurotoxicity of antisense oligonucleotides through strategic chemical modifications
Source: Mol Ther Nucleic Acids. 2025 Sep 12;36(4):102692. doi: 10.1016/j.omtn.2025.102692 (PMC12744863; doi:10.1016/j.omtn.2025.102692)
Supplement: Document S1. Figures S1–S26 and Tables S1–S3 [file mmc1.pdf]

OMTN, Volume *36*

Supplemental information

Unraveling and controlling late-onset neurotoxicity of antisense oligonucleotides through strategic chemical modifications

Takayuki Kuroda, Kotaro Yoshioka, Su Su Lei Mon, Maho Katsuyama, Kumiko Sato, Eriko Isogai, Kie Yoshida-Tanaka, Rintaro Iwata-Hara, Takao Yamaguchi, Satoshi Obika, and Takanori Yokota

Table S1. Antisense oligonucleotide sequences.

Mapt: microtubule-associated protein tau; *Hdac2*: histone deacetylase 2; *SNCA*: synuclein alpha; *HTT*: huntingtin. Bold letters indicate LNA, bold and italic letters indicate 2′-MOE, and regular letters indicate DNA. C(5) denotes 5-methylcytosine. All internucleoside linkages are phosphorothioate (PS)-bonds, except at underlined positions, which indicate substitution of the PS-bond with a phosphodiester (PO)-bond at the 5′-adjacent site of the DNA monomer.

Name	Target mRNA	Sequence
**ASO1**	*Mapt*	**5′- ATT**TCCAAATTCA**C(5)TT**
**ASO2**	*Hdac2*	**5′- TAG**TCTCTGTCAG**TTA**
**ASO3**	*SNCA*	**5′- C(5)TC(5)A**CCATTTATATAC**AAAC(5)**
**ASO4**	*SNCA*	**5′- C(5)C(5)A**TTTATATACAAA**C(5)AC(5)A**
**ASO5**	*HTT*	**5′-** ***C(5)T<u>C(5)AG</u>***TAAC(5)ATTGAC(5)***A<u>C(5)C(5)A</u>C(5)***

Table S2. Tolerability scoring system.

The tolerability scoring system used in the in mouse (**A**) and rat (**B**) experiments. N.A.: not applicable.

A

		Score 1	Score 2	Score 3	Score 4
Category 1 Consciousness	Decreased exploration or voluntary movement	Decreased exploration	Decreased exploration	No exploration	No exploration
	Decreased responsiveness	Normal	Slightly (e.g., reduced response to touch or handling)	Moderately (e.g., reduced response to lift, no blink)	Marked (e.g., reduced response to a tail pinch)
Category 2 Motor function	Ataxia	(-)	Mild (e.g., slow righting response, swaying)	Moderate (e.g., staggering, impaired walking)	Severe (e.g., crawling)
	Strength	Decreased strength of lower limbs	No ability to climb a slope	No ability to walk	No ability to right
Category 3 Appearance	Abnormal posture	Slight	Mild (e.g., hunched, extended, low posture, straub tail)	Moderate (e.g., ventral recumbency)	Severe (e.g., lateral recumbency)
	Abnormal breathing	Normal	Normal	Shallow	Labored
Category 4 Hyperactivity	Increased home-cage exploration	Slightly increased (e.g., increased exploration)	Increased (e.g., digging, burying)	Moderately increased (e.g., scratching limbs)	Marked increased
	Stereotype	(-)	Slight (e.g., increased grooming)	Moderate (e.g., circling, repetitive behavior)	Marked
Category 5 Involuntary movement	Tremors	Detectable	Marked	N.A.	N.A.
	Seizure (e.g., running, bouncing, clonic and/or tonic seizures)	(-)	(-)	Few or partial	Repeated or continuous (e.g., >1 min)

B

		Score 1	Score 2	Score 3	Score 4
Category 1 Consciousness	Decreased exploration or voluntary movement	Decreased exploration	Decreased exploration	No exploration	No exploration
	Decreased responsiveness	Normal	Slightly (e.g., reduced response to touch or handling)	Moderately (e.g., reduced response to lift, no blink)	Marked (e.g., reduced response to a tail pinch)
Category 2 Motor function	Ataxia	Slight (e.g., swaying)	Mild (e.g., crawling)	Moderate (e.g., rolling around)	Severe (e.g., no ability to move)
	Strength	Decreased strength of lower limbs	No ability to walk on four limbs	No ability to right without movement of lower limbs	No movement of four limbs
Category 3 Appearance	Abnormal posture	Slight	Mild (e.g., hunched, extended, low posture, straub tail)	Moderate (e.g., ventral recumbency with head up)	Severe (e.g., ventral recumbency without head up)
	Abnormal breathing	Normal	Normal	Shallow	Labored
Category 4 Hyperactivity	Increased home-cage exploration	Slightly increased (e.g., increased exploration)	Increased (e.g., digging, burying)	Moderately increased (e.g., scratching limbs)	Marked increased
	Stereotype	(-)	Slight (e.g., increased grooming)	Moderate (e.g., circling, repetitive behavior)	Marked
Category 5 Involuntary movement	Tremors	Detectable	Marked	N.A.	N.A.
	Seizure (e.g., running, bouncing, clonic and/or tonic seizures)	(-)	(-)	Few or partial	Repeated or continuous (e.g., >1 min)

Table S3. Primer and probe sets for qRT-PCR.

Mouse	*Gapdh*	Forward	5′ -GGTTGTCTCCTGCGACTTCAA-3′
		Reverse	5′ -CCGTATTCATTGTCATACCAGGAAA-3′
		Probe	5′ -CTCCCACTCTTCCACCTTCGATGCCG-3′
Human	*ACTB*	Forward	5′ -CGGACTATGACTTAGTTGCGTTACA-3′
		Reverse	5′ -GCCATGCCAATCTCATCTTGT-3′
		Probe	5′ -CCTTTCTTGACAAAACCTAACTTGCGCAGA-3′
	GAPDH	Forward	5′ -GAAGGTGAAGGTCGGAGTC-3′
		Reverse	5′ -GAAGATGGTGATGGGATTTC-3′
		Probe	5′ -CAAGCTTCCCGTTCTCAGCC-3′

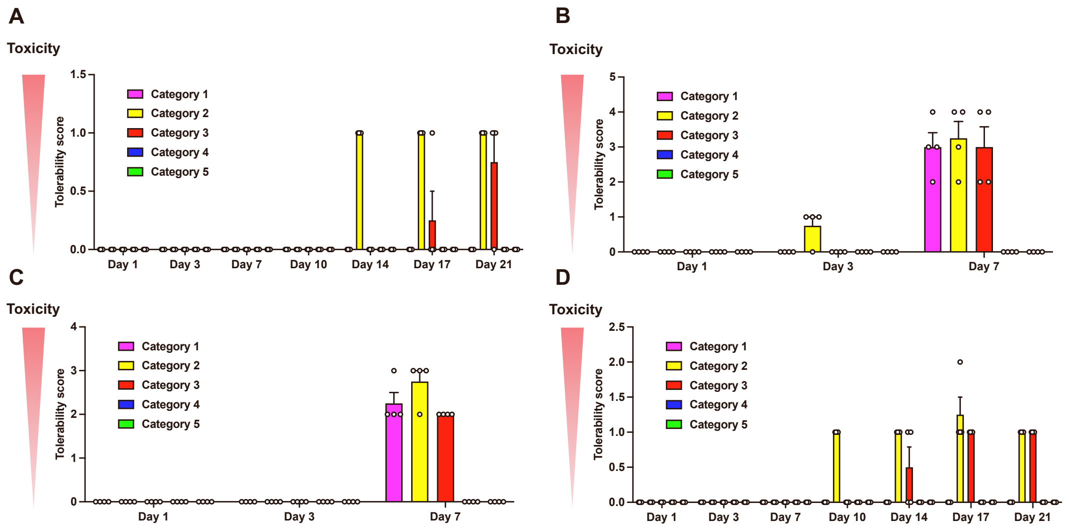

Figure S1. Breakdown of tolerability score by category in mice post-PS-ASO injection.

Seven-week-old female C57BL/6 mice (n = 4 per group) were injected intracerebroventricularly with (**A**) ASO1 (38.4 nmol, 200 µg), (**B**) ASO2 (19.0 nmol, 100 µg), (**C**) ASO3 (15.2 nmol, 100 µg), (**D**) ASO4 (39.9 nmol, 250 µg). Category1: consciousness; Category 2: motor function; Category 3: appearance; Category 4: hyperactivity; Category 5: involuntary movement. Data are presented as mean ± SEM.

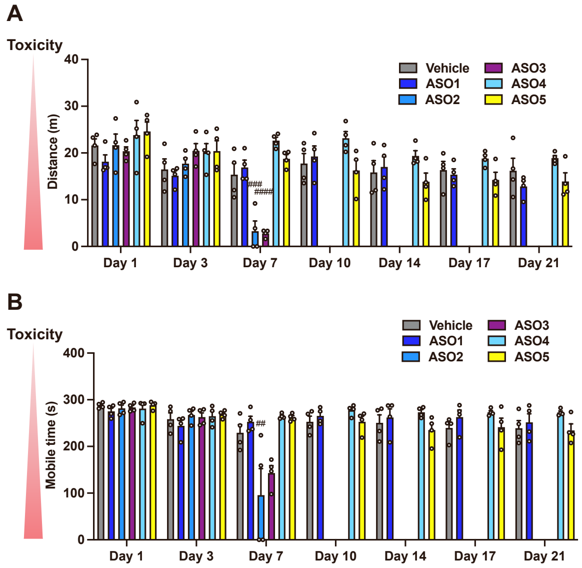

Figure S2. Distance traveled and mobile time in open-field tests in mice post-PS-ASO injection.

Seven-week-old female C57BL/6 mice (n = 4 per group) were injected intracerebroventricularly with ASO1 (38.4 nmol, 200 µg), ASO2 (19.0 nmol, 100 µg), ASO3 (15.2 nmol, 100 µg), ASO4 (39.9 nmol, 250 µg), or ASO5 (39.9 nmol, 280 µg). Mice treated with ASO2 and ASO3 were evaluated up to day 7. (**A**) Distance traveled and (**B**) mobile time in open-field tests. Data are presented as mean ± SEM. # indicates significance against vehicle.

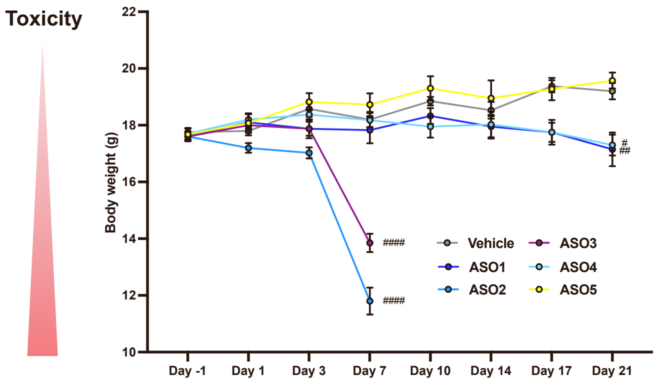

Figure S3. Changes in body weight in mice post-PS-ASO injection.

Seven-week-old female C57BL/6 mice (n = 4 per group) were injected intracerebroventricularly with ASO1 (38.4 nmol, 200 µg), ASO2 (19.0 nmol, 100 µg), ASO3 (15.2 nmol, 100 µg), ASO4 (39.9 nmol, 250 µg), or ASO5 (39.9 nmol, 280 µg). Mice treated with ASO2 and ASO3 were evaluated up to day 7. Data are presented as mean ± SEM. # indicates significance against vehicle.

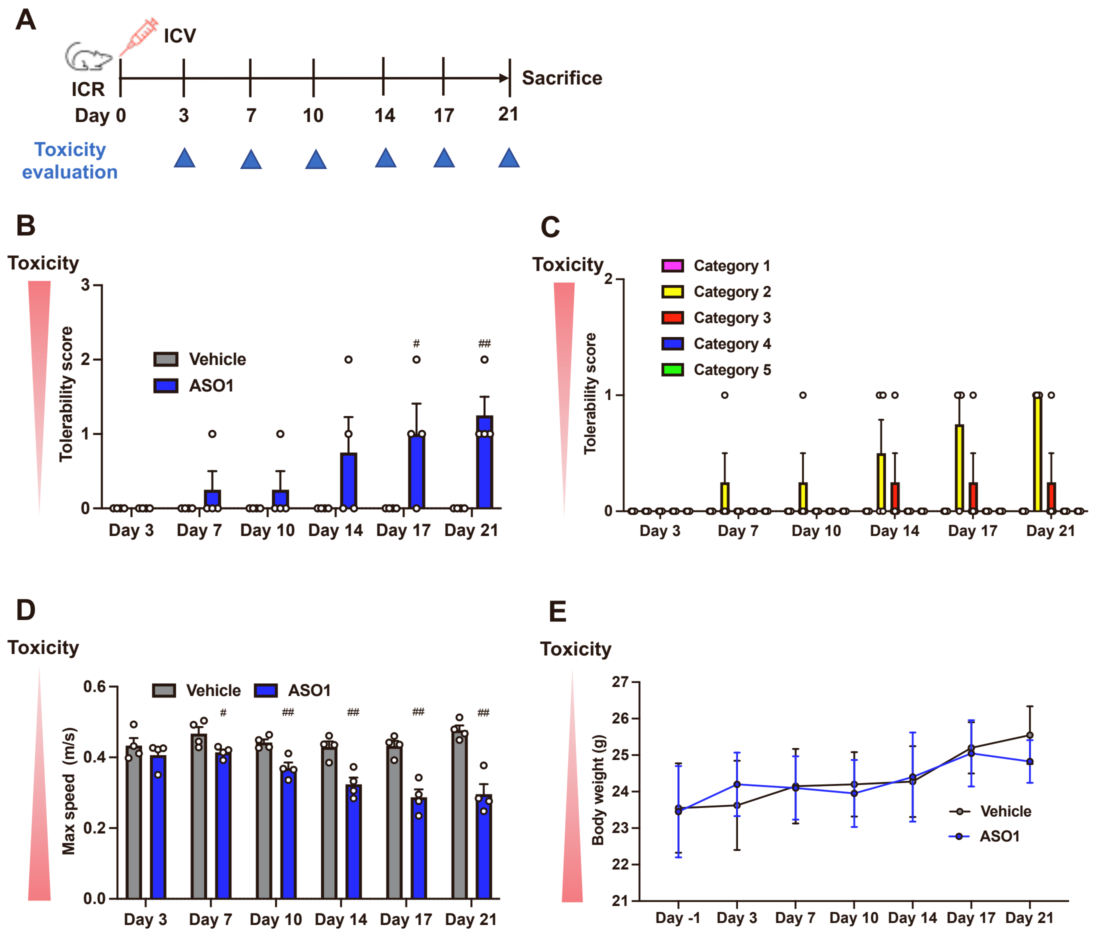

Figure S4. Late-onset neurotoxicity in mice after ICV-injection of ASO1.

(**A-E**) Seven-week-old female ICR mice (n = 4 per group) were injected intracerebroventricularly with 38.4 nmol (200 µg) of ASO1. Toxicity was assessed on the indicated days, and mice were sacrificed 21 days postinjection. (**B**) Tolerability scores over time postinjection. (**C**) Breakdown of tolerability score by category. (**D**) Maximum speed in open-field tests at the indicated time points. (**E**) Changes in body weight over time. Data are presented as mean ± SEM. # indicates significance against vehicle.

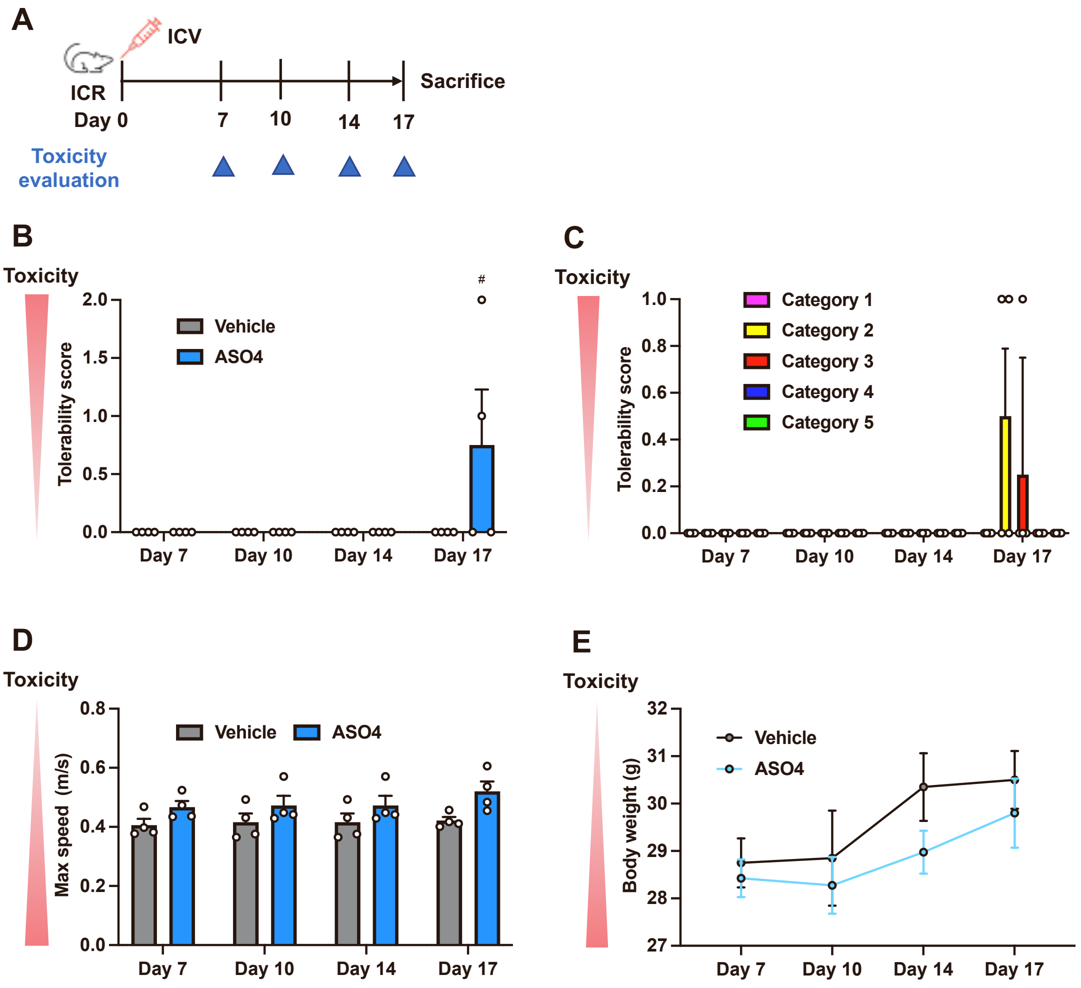

Figure S5. Late-onset neurotoxicity in mice after ICV-injection of ASO4.

(**A-E**) Seven-week-old female ICR mice (n = 4 per group) were injected intracerebroventricularly with 39.9 nmol (250 µg) ASO4. Toxicity was assessed on the indicated days, and mice were sacrificed 17 days postinjection. (**B**) Tolerability scores over time postinjection. (**C**) Breakdown of tolerability score by category. (**D**) Maximum speed in open-field tests at the indicated time points. (**E**) Changes in body weight over time. Data are presented as mean ± SEM. # indicates significance against vehicle.

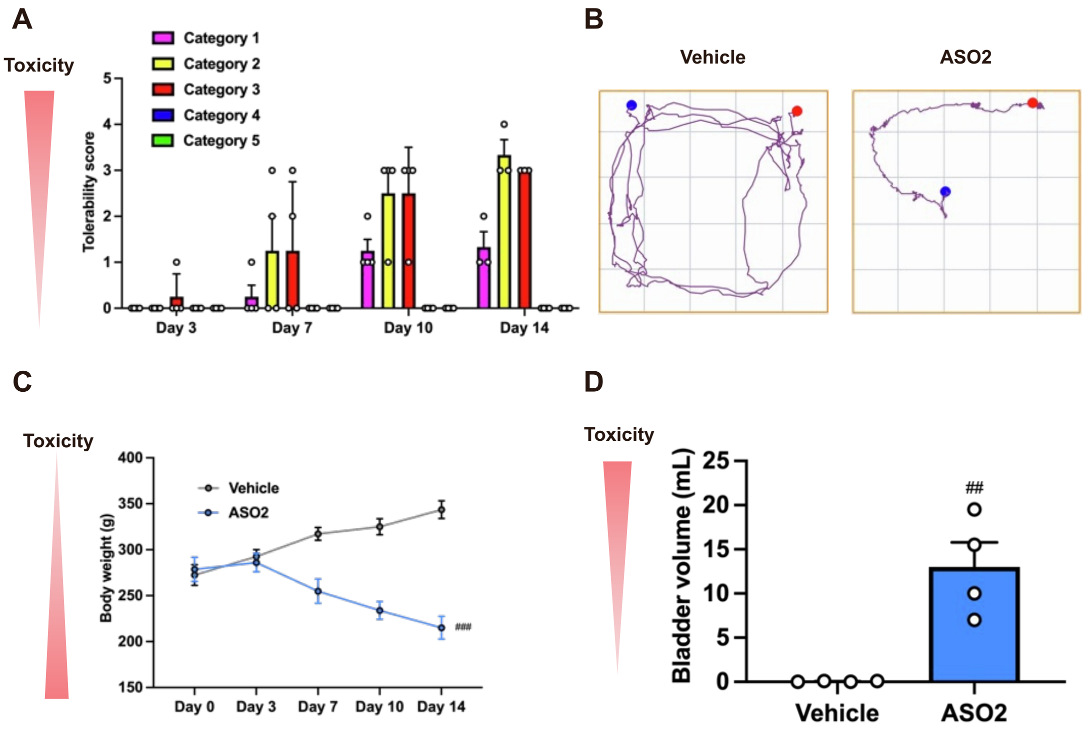

Figure S6. Late-onset neurotoxicity in rats after ICV-injection of ASO2.

Nine-week-old male Slc:SD rats injected intrathecally via spinal canal catheters with 190 nmol (1.0 mg) of ASO2. (**A**) Breakdown of tolerability score by category. (**B**) Representative track plots in open-field tests 14 days postinjection. (**C**) Changes in body weight. (**D**) Bladder volume at sacrifice (14 days postinjection). Data are presented as mean ± SEM. # indicates significance against vehicle.

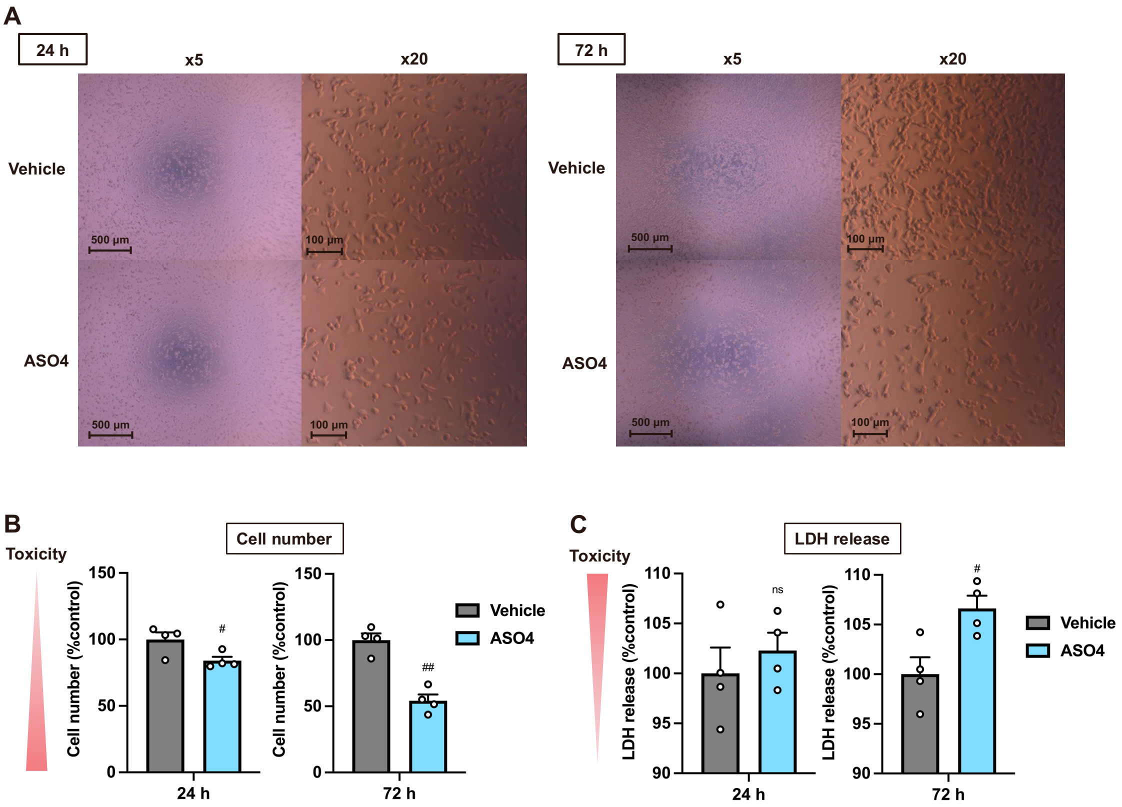

Figure S7. Neuronal cytotoxicity of ASO4 in BE(2)-M17 cells

BE(2)-M17 cells were transfected with 50 nM ASO4. (**A**) Representative microscopic images of cells 24 and 72 h after transfection. (**B**) cell number 24 h and 72 h post-transfection. (**C**) LDH release in medium 24 and 72 h post-transfection. Data in (**B, C**) are presented as mean ± SEM, as a percentage relative to the vehicle (n = 4 per group). Ns, not significant ($p > 0.05$). # indicates significance against vehicle.

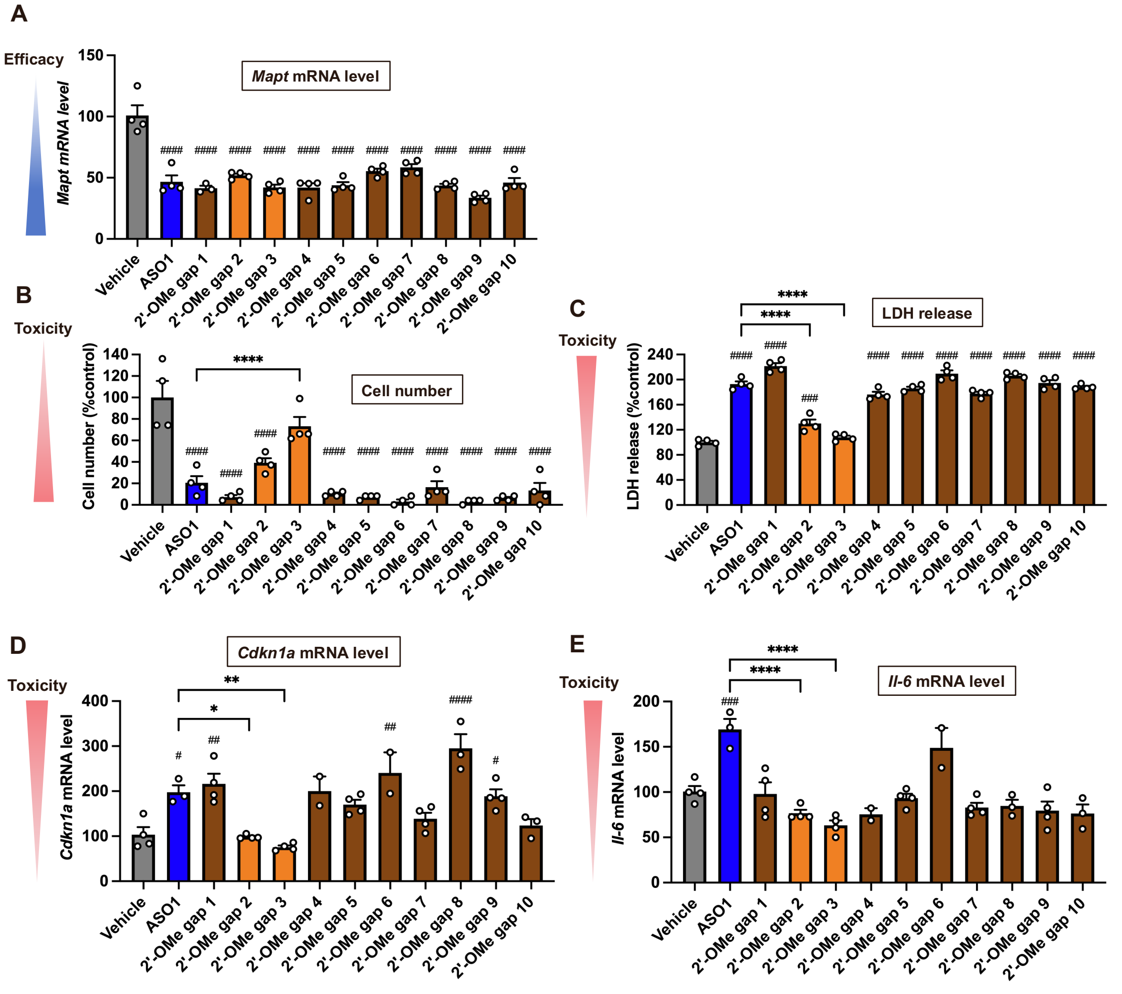

Figure S8. In vitro assessment of 2′-OMe gap walking in ASO1.

Neuro-2a cells were transfected with 4 nM ASO1 (knockdown activity assessment) or 50 nM ASO1 (toxicity assessment), with or without 2′-OMe modification at each gap position. Target *Mapt* mRNA levels (**A**), cell number (**B**). LDH release (**C**), *Cdkn1a* mRNA levels (**D**), and *Il-6* mRNA levels (**E**) 72 h post-transfection are presented as mean ± SEM, as a percentage relative to the vehicle (n = 2 per group for *Il-6* mRNA of ASO1 with 2′-OMe at gap position 4; n = 4 per group for the others). The highlighted sequences with 2′-OMe modifications show an increase in a cell number and a decrease in LDH release compared to ASO1. # and * indicates significance against vehicle and ASO1, respectively.

A

Sequence ASO4	Modification	T_m and ΔT_m (℃)	*SNCA* mRNA level (%control)	Cell number (%control)	LDH release (%control)	*CDKN1A* mRNA level (%control)
CCATTTATATACAAACACA	Parent	57	40	53	113	231
CCATTTATATACAAACACA	2′-OMe gap 1	+1	62 (+22)	49 (-4)	111 (-2)	293 (+62)
CCATTTATATACAAACACA	2′-OMe gap 2	+0	67 (+27)	61 (+8)	106 (-7)	368 (+137)
CCATTTATATACAAACACA	2′-OMe gap 3	+1	59 (+19)	51 (-2)	112 (-1)	210 (-21)
CCATTTATATACAAACACA	2′-OMe gap 4	-1	63 (+23)	66 (+13)	108 (-5)	146 (-85)
CCATTTATATACAAACACA	2′-OMe gap 5	+0	66 (+26)	54 (+1)	113 (+0)	93 (-138)
CCATTTATATACAAACACA	2′-OMe gap 6	-2	66 (+26)	60 (+7)	113 (+0)	41 (-190)

Sequence ASO4	Modification	T_m and ΔT_m (℃)	*SNCA* mRNA level (%control)	Cell number (%control)	LDH release (%control)	*CDKN1A* mRNA level (%control)
CCATTTATATACAAACACA	Parent	57	39	58	107	409
CCATTTATATACAAACACA	2′-OMe gap 7	+0	60 (+21)	48 (-10)	103 (-4)	386 (-23)
CCATTTATATACAAACACA	2′-OMe gap 8	-1	58 (+19)	63 (+5)	107 (+0)	391 (-18)
CCATTTATATACAAACACA	2′-OMe gap 9	+0	54 (+15)	50 (-8)	110 (+3)	269 (-140)
CCATTTATATACAAACACA	2′-OMe gap 10	-1	46 (+7)	52 (-6)	112 (+5)	417 (+8)
CCATTTATATACAAACACA	2′-OMe gap 11	+1	41 (+2)	47 (-11)	112 (+5)	384 (-25)
CCATTTATATACAAACACA	2′-OMe gap 12	-1	51 (+12)	48 (-10)	114 (+7)	365 (-44)

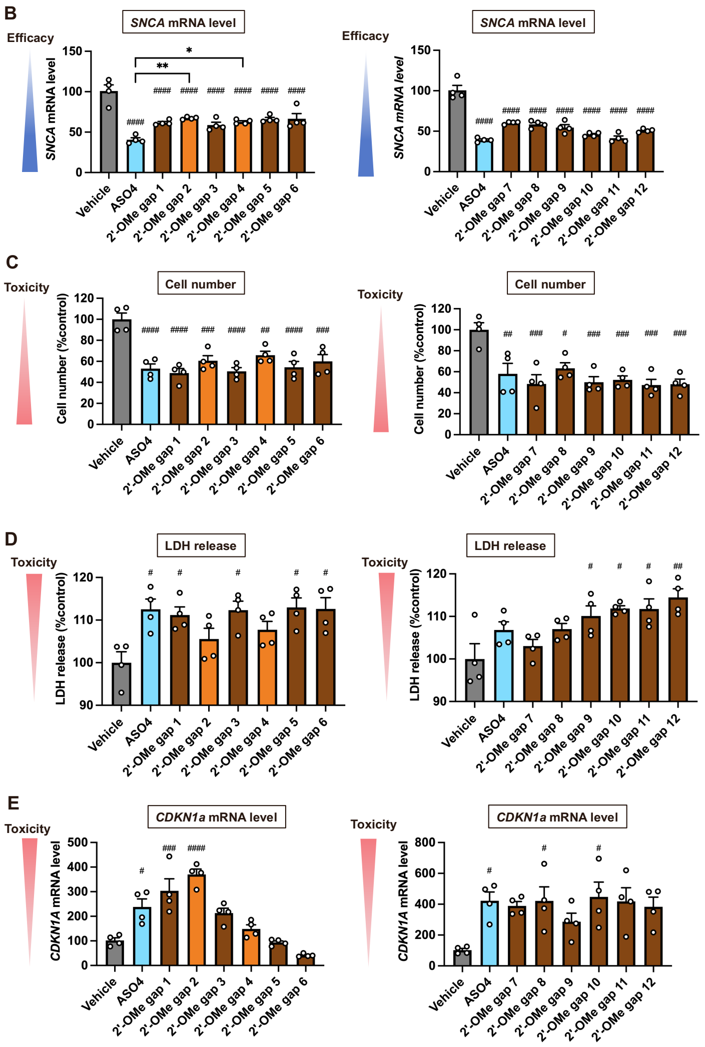

Figure S9. In vitro assessment of 2′-OMe gap walking in ASO4.

(**A**) BE(2)-M17 cells were transfected with 1 nM ASO4 (knockdown activity assessment) or 50 nM ASO4 (toxicity assessment), with or without 2′-OMe modification at each gap position. Blue letters indicate LNA, whereas blue “C” indicates LNA with 5-methylcytosine. Black letters indicate DNA and red letters indicate 2′-OMe modified DNA. ΔT_m indicates the T_m of the sequence minus the T_m of the parent ASO. Target mRNA levels, cell number, LDH release, and *CDKN1A* mRNA levels 72 h post-transfection are shown as the mean percentage relative to vehicle (n = 4 per group). The highlighted sequences with 2′-OMe modifications show an increase in cell number and a decrease in LDH release compared to the parent ASOs. (**B-E**) Dot plots for (**A**): Target *SNCA* mRNA levels (**B**), cell number (**C**). LDH release (**D**), and *CDKN1A* mRNA levels (**E**) 72 h post-transfection. Data are presented as mean ± SEM, as a percentage relative to the vehicle. The highlighted sequences with 2′-OMe modifications show an increase in cell number and a decrease in LDH release compared to ASO4. # and * indicates significance against vehicle and ASO4, respectively.

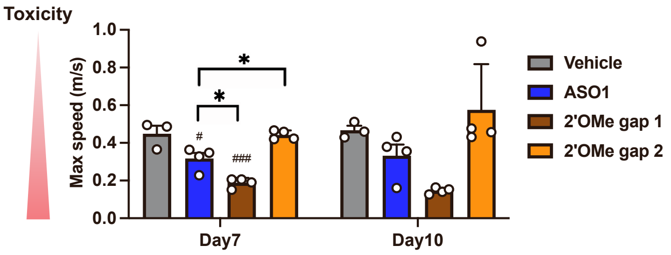

Figure S10. 2′-OMe at gap position 2 reduces late-onset neurotoxicity of ASO1 via ICV-injection.

Seven-week-old female ICR mice (n = 3 per group for vehicle, and n = 4 per group for all other groups) were injected intracerebroventricularly with 38.4 nmol of ASO1 and ASO1 modified with 2′-OMe at gap position 1 or 2. Maximum speed in open-field tests at the indicated time points. Data are presented as mean ± SEM. # and * indicates significance against vehicle and ASO1, respectively.

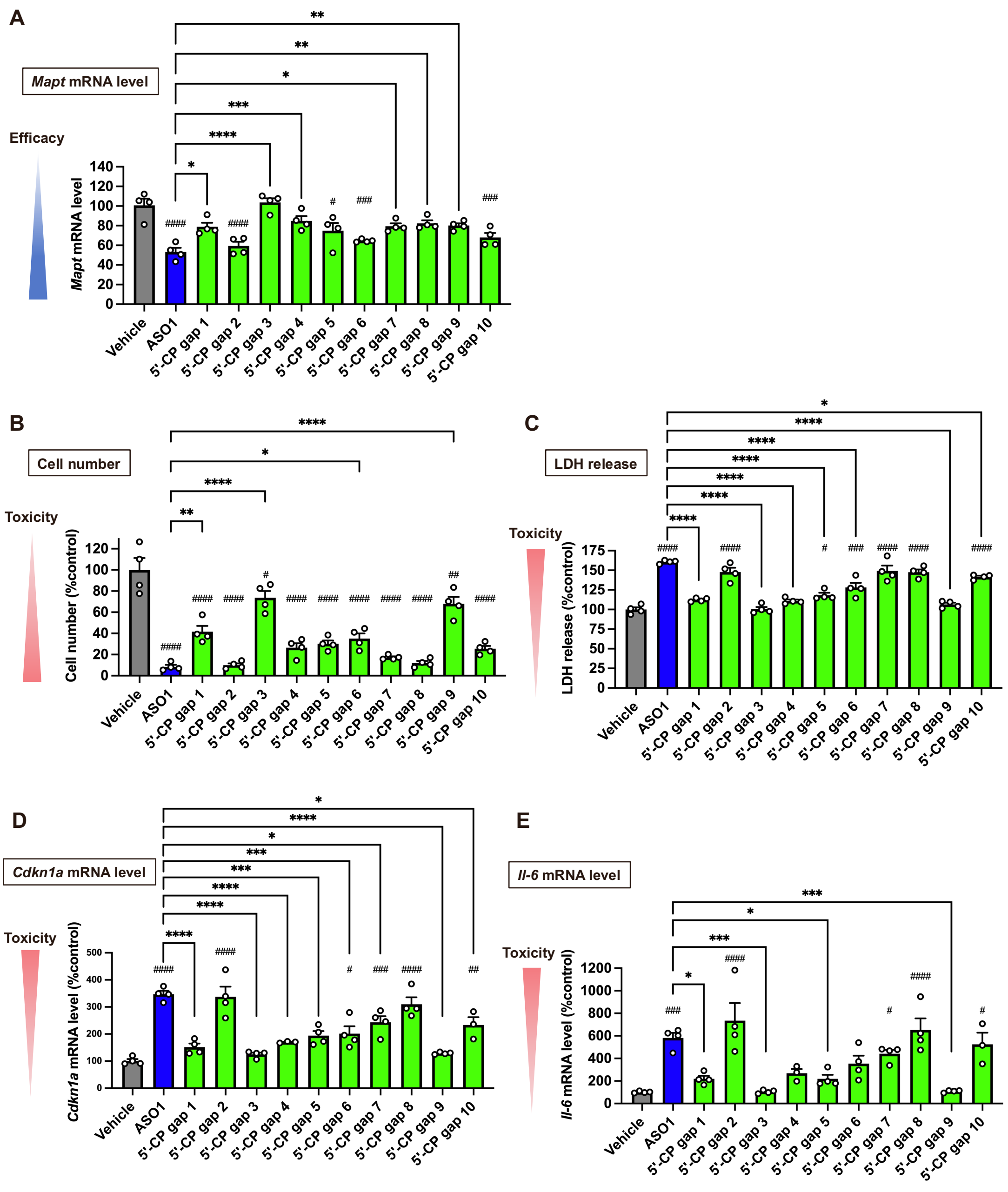

Figure S11. In vitro assessment of 5′-CP gap walking in ASO1.

Neuro-2a cells were transfected with 4 nM ASO1 (knockdown activity assessment) or 50 nM ASO1 (toxicity assessment), with or without 5′-CP modification at each gap position. Target *Mapt* mRNA levels (**A**), cell number (**B**). LDH release (**C**), *Cdkn1a* mRNA levels (**D**), and *Il-6* mRNA levels (**E**) 72 h post-transfection are presented as mean ± SEM, as a percentage relative to the vehicle (n = 3 per group for *Cdkn1a* and *Il-6* mRNA of ASO1 with 5′-CP at gap position 4 and 10; n = 4 per group for others). # and * indicates significance against vehicle and ASO1, respectively.

A

Sequence ASO4	Modification	T_m and ΔT_m (℃)	*SNCA* mRNA level (%control)	Cell number (%control)	LDH release (%control)	*CDKN1A* mRNA level (%control)
CCATTTATATACAAACACA	Parent	57	35	52	106	346
CCATTTATATACAAACACA	5′-CP gap 1	-2	65 (+30)	70 (+18)	100 (-6)	182 (-164)
CCATTTATATACAAACACA	5′-CP gap 2	-2	63 (+28)	64 (+12)	100 (-6)	207 (+139)
CCATTTATATACAAACACA	5′-CP gap 3	-2	58 (+23)	58 (+6)	100 (-6)	235 (-111)
CCATTTATATACAAACACA	5′-CP gap 4	-1	39 (+4)	44 (-8)	103 (-3)	337 (-9)
CCATTTATATACAAACACA	5′-CP gap 5	-2	47 (+12)	46 (-6)	103 (-3)	240 (-106)
CCATTTATATACAAACACA	5′-CP gap 6	-1	43 (+8)	30 (-22)	108 (+2)	325 (-21)

Sequence ASO4	Modification	T_m and ΔT_m (℃)	*SNCA* mRNA level (%control)	Cell number (%control)	LDH release (%control)	*CDKN1A* mRNA level (%control)
CCATTTATATACAAACACA	Parent	57	31	59	105	217
CCATTTATATACAAACACA	5′-CP gap 7	-2	44 (+13)	77 (+18)	102 (-3)	157 (-60)
CCATTTATATACAAACACA	5′-CP gap 8	-2	43 (+12)	78 (+19)	100 (-5)	186 (-31)
CCATTTATATACAAACACA	5′-CP gap 9	-2	46 (+15)	79 (+20)	101 (-4)	134 (-83)
CCATTTATATACAAACACA	5′-CP gap 10	-3	49 (+18)	63 (+4)	106 (+1)	177 (-40)
CCATTTATATACAAACACA	5′-CP gap 11	-2	43 (+8)	47 (-12)	111 (+6)	297 (+80)
CCATTTATATACAAACACA	5′-CP gap 12	-2	47 (+16)	68 (+9)	104 (-1)	170 (-47)

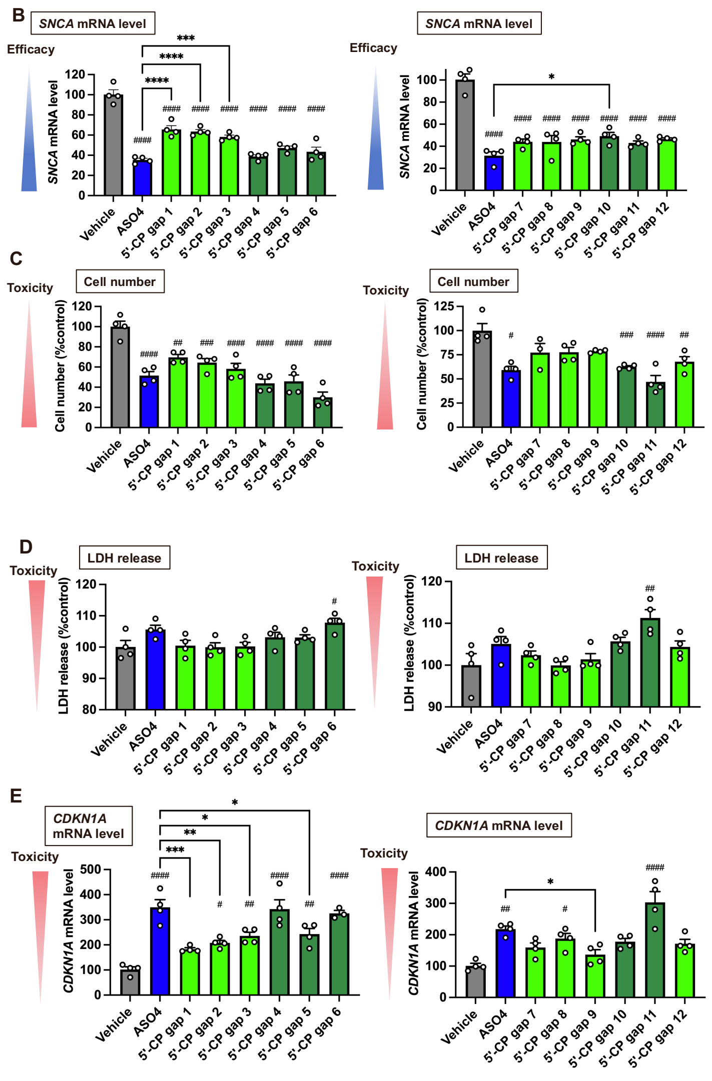

Figure S12. In vitro assessment of 5′-CP gap walking in ASO4.

(**A**) BE(2)-M17 cells were transfected with 1 or 50 nM ASO4, with or without 5′-CP at each gap position. Blue letters indicate LNA, whereas blue "C" indicates LNA with 5-methylcytosine. Black letters indicate DNA and green letters indicate 5′-CP-modified DNA. ΔT_m indicates the T_m of the sequence minus the T_m of the parent ASO. Target mRNA levels, cell number, LDH release, and *CDKN1A* mRNA levels 72 h post-transfection are shown as the mean percentage relative to vehicle (n = 4 per group). The highlighted sequences with 5′-CP modifications show an increase in cell number and a decrease in LDH release compared to the parent ASOs. (**B**-**E**) Dot plots for (**A**): Target *SNCA* mRNA levels (**B**), cell number (**C**). LDH release (**D**), and *CDKN1A* mRNA levels (**E**) 72 h post-transfection. Data are presented as mean ± SEM, as a percentage relative to the vehicle. The highlighted sequences with 5′-CP modifications show an increase in cell number and a decrease in LDH release compared to ASO4. # and * indicates significance against vehicle and ASO4, respectively.

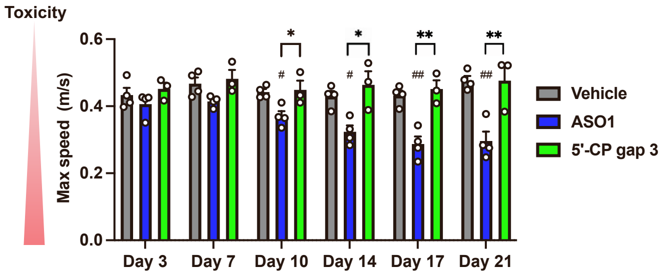

Figure S13. 5′-CP at gap position 3 mitigates late-onset neurotoxicity of ASO1 via ICV-injection.

Seven-week-old female ICR mice (n = 3 per group for ASO1 with 5′-CP at gap position 3, n= 4 per group for other groups) were injected intracerebroventricularly with 38.4 nmol of ASO1, with or without 5′-CP at gap position 3. Maximum speed in open-field tests were recorded on the indicated days postinjection. Data are presented as mean ± SEM. # and * indicates significance against vehicle and ASO1, respectively.

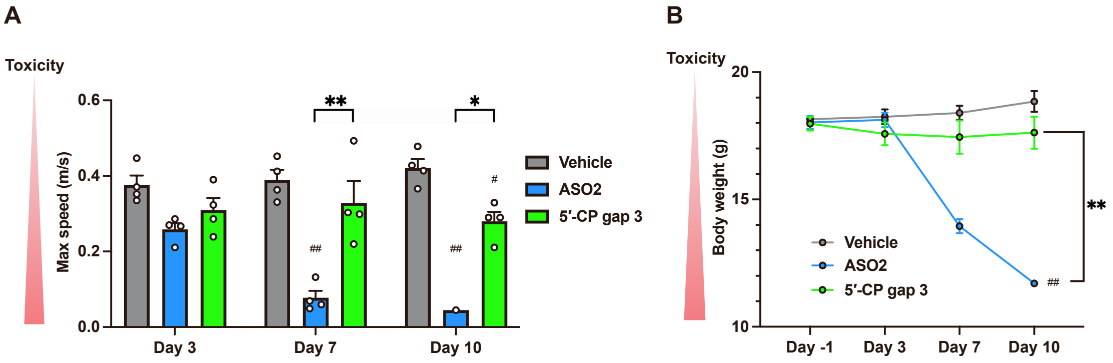

Figure S14. 5′-CP at gap position 3 mitigates late-onset neurotoxicity of ASO2 via ICV-injection.

Seven-week-old female C57BL/6 mice (n = 4 per group) were injected intracerebroventricularly with 19.0 nmol of ASO2, with or without 5′-CP modification at gap position 3. Mice were sacrificed 11 days postinjection. (**A**) Maximum speed in open-field tests were recorded on the indicated days postinjection. (**B**) Changes in body weight over time. Data are presented as mean ± SEM. # and * indicates significance against vehicle and ASO2, respectively.

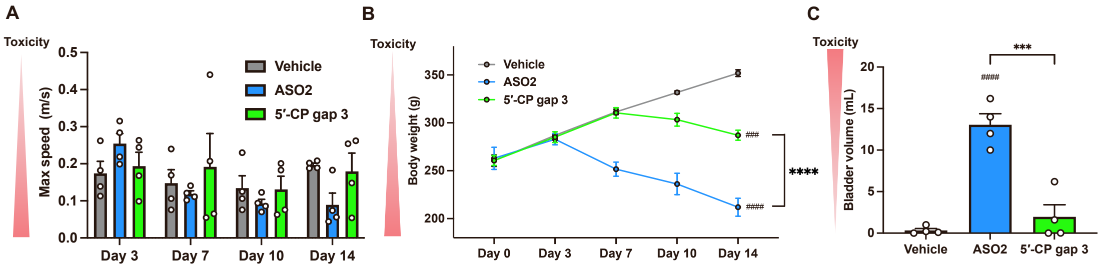

Figure S15. 5′-CP at gap position 3 mitigates late-onset neurotoxicity of ASO2 via IT-injection in rats.

Nine-week-old male Slc:SD rats (n = 4 per group) were injected intrathecally via spinal canal catheters with 190 nmol of ASO2, with or without 5′-CP modification at gap position 3. Rats were sacrificed 14 days postinjection. (**A**) Maximum speed in open-field tests were documented on the indicated days postinjection. (**B**) Changes in Body weight over time. (**C**) Bladder volume at sacrifice. Data are presented as mean ± SEM. # and * indicates significance against vehicle and ASO2, respectively.

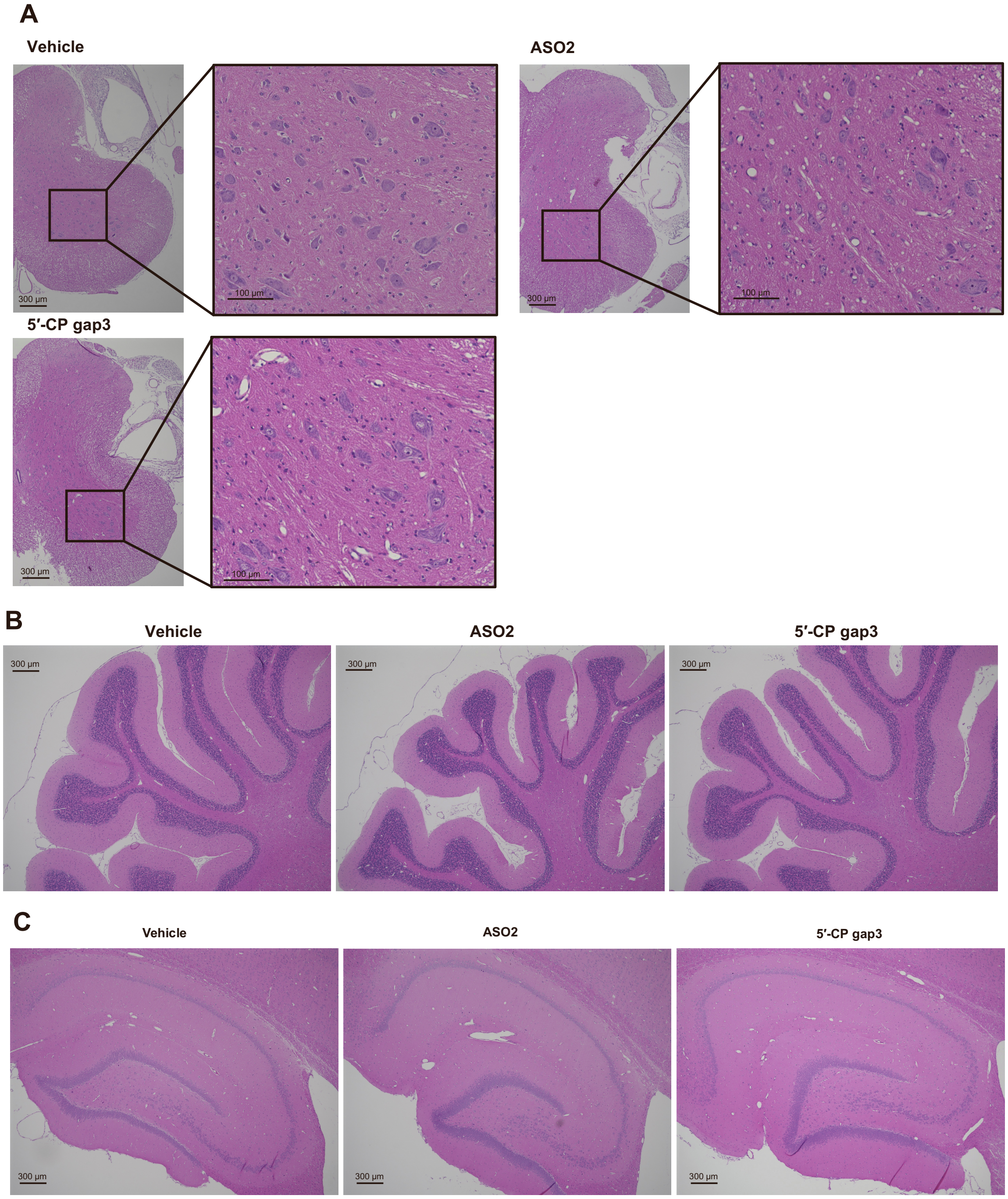

Figure S16. Representative H&E-stained images of rat lumbar cord (A), cerebellum (B), and hippocampus (C), 10 days after intrathecal injection of 190 nmol of ASO2, with or without 5′-CP modification at gap position 3.

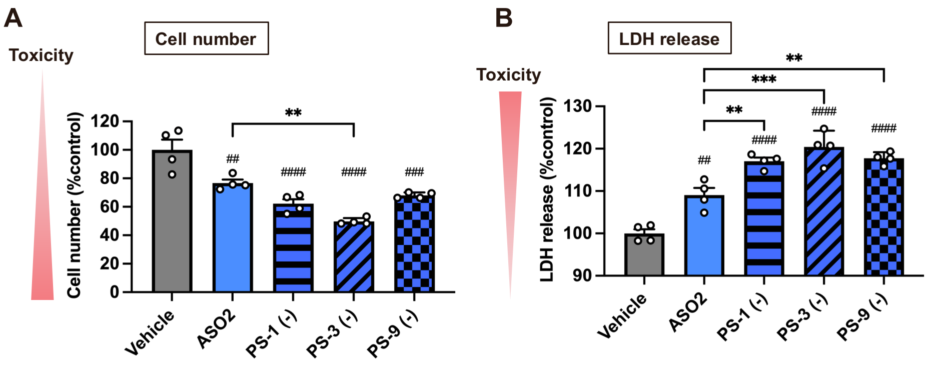

Figure S17. In vitro assessment of PS-1(-), PS-3(-), or PS-9(-) in the gap region in ASO2.

BE(2)-M17 cells were transfected with 20 nM ASO2 with or without replacement of a PS bond 5′-adjacent a DNA monomer at gap position 1, 3, or 9 with a PO bond (PS-1(-), PS-3(-), or PS-9(-)). Cell number (**A**) and LDH release (**B**) 72 h post-transfection are presented as mean ± SEM, as a percentage relative to the vehicle (n = 4 per group). # and * indicates significance against vehicle and ASO2, respectively.

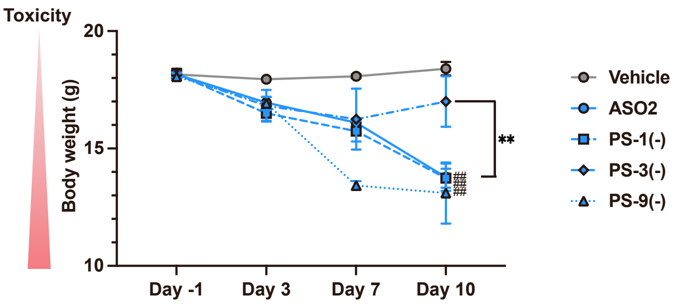

Figure S18. Changes in body weight in mice after ICV-injection of ASO2 with PS-1(-), PS-3(-), and PS-3(-).

Seven-week-old female C57BL/6 mice (n = 4 per group for vehicle and the parent ASO2; n=5 per group for the others) were injected intracerebroventricularly with 19.0 nmol of ASO2 or ASO2 with PS-1(-), PS-3(-), or PS-9(-). Toxicity was assessed on the indicated days, and mice were sacrificed 10 days postinjection. ** indicates a p-value ($p \leq 0.01$) between parent ASO2 and ASO2 PS-3(-). Data are presented as mean ± SEM. # indicates significance against vehicle.

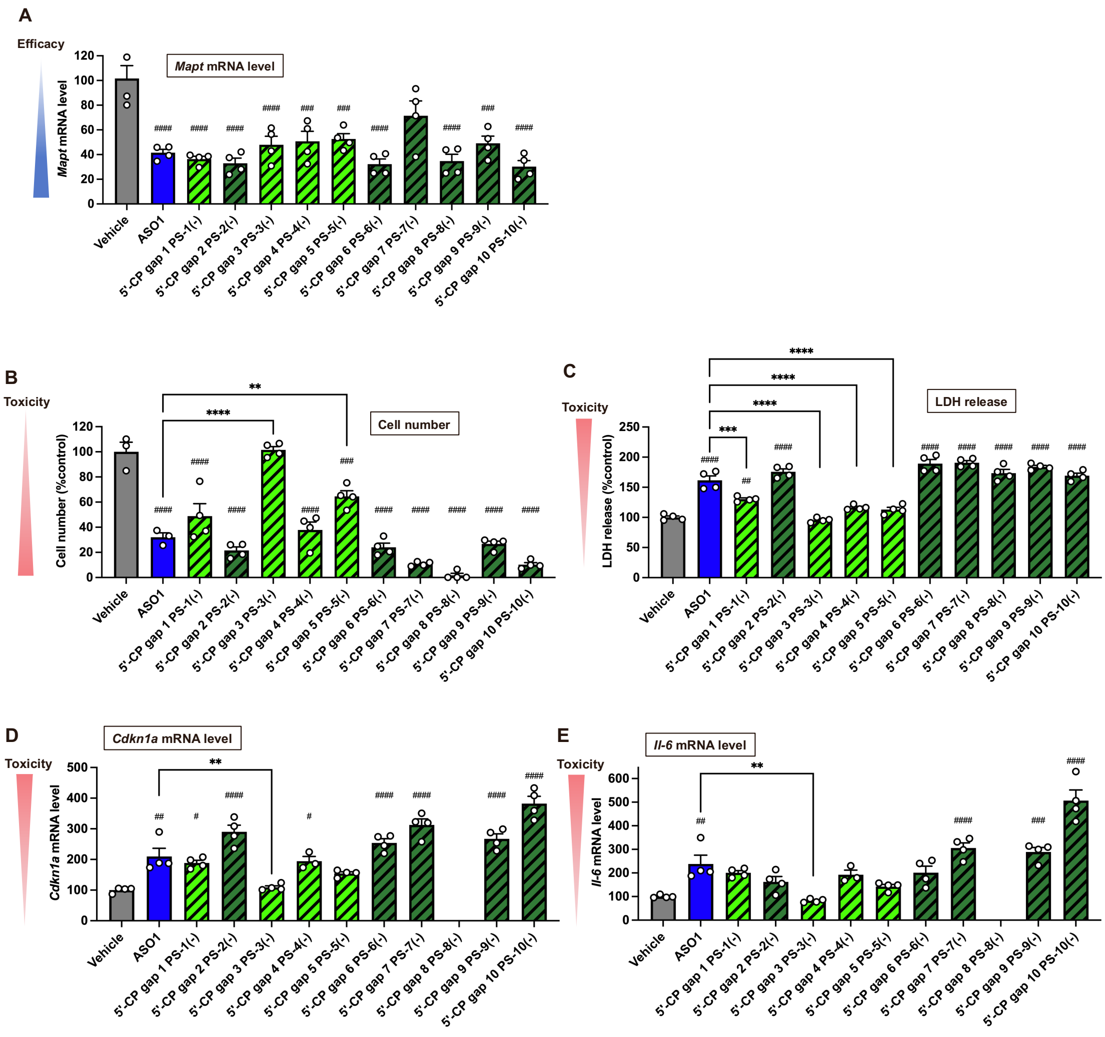

Figure S19. In vitro assessment of 5′-CP with PO-replacement gap walking in ASO1.

Neuro-2a cells were transfected with 4 nM ASO1 (knockdown activity assessment) or 50 nM ASO1 (toxicity assessment), with or without replacement of 5′-CP modification and the 5′-adjacent PS-bond with PO-bond at each gap position. Target *Mapt* mRNA levels (**A**), cell number (**B**). LDH release (**C**), *Cdkn1a* mRNA levels (**D**), and *Il-6* mRNA levels (**E**) 72 h post-transfection are presented as mean ± SEM, as a percentage relative to the vehicle. The highlighted sequences with 5′-CP modifications show an increase in a cell number and a decrease in LDH release compared to ASO1. For cell number, n = 3 per group in vehicle and parent ASO1; n = 4 per group for the others. *Cdkn1a* and *Il-6* mRNA levels for ASO1 with 5′-CP at gap position 8 PS-8(-) were unmeasurable due to insufficient RNA retraction with severe cytotoxicity. # and * indicates significance against vehicle and ASO1, respectively.

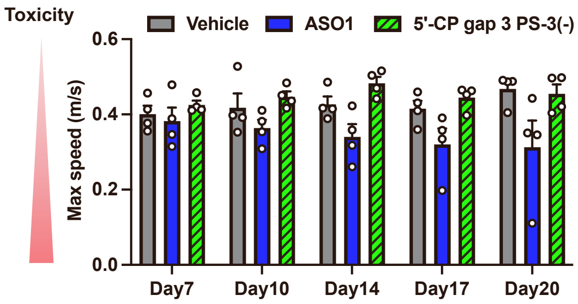

Figure S20. 5′-CP at gap position 3 with PS-3(-) mitigate late-onset neurotoxicity of ASO1 via ICV-injection.

Seven-week-old female ICR mice (n = 4 per group) were injected intracerebroventricularly with 38.4 nmol of ASO1 and ASO1 with 5′-CP at gap position 3 with PS-3(-). Toxicity was assessed on the indicated days, and mice were sacrificed 21 days postinjection. Maximum speed in open-field tests were documented on the indicated days postinjection. Data are presented as mean ± SEM.

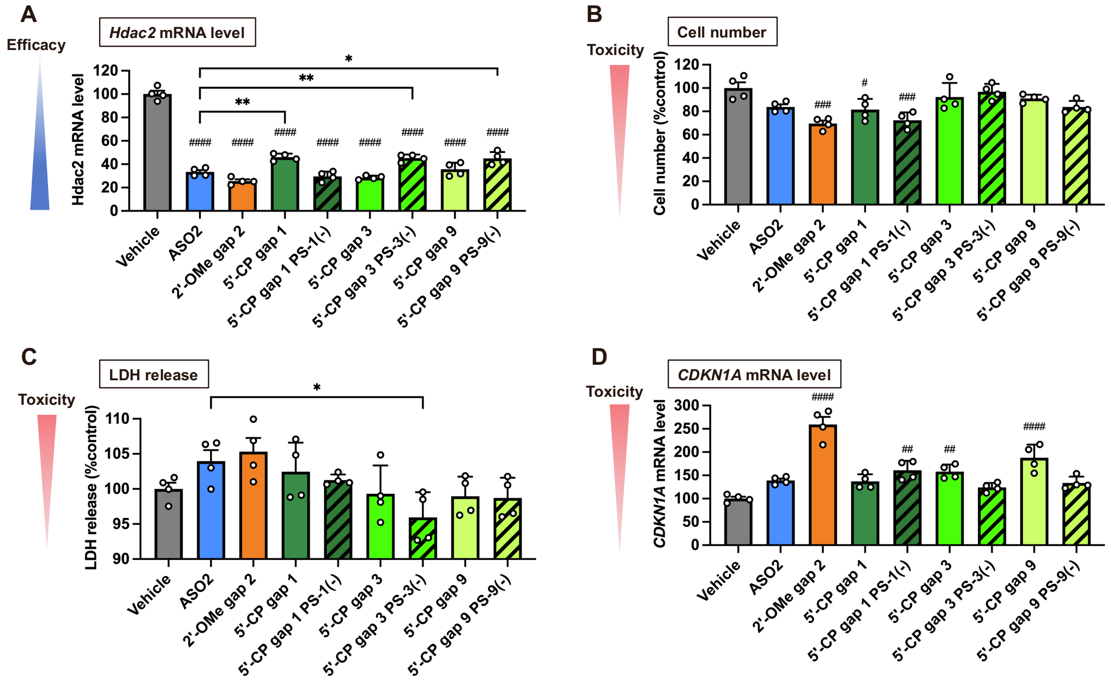

Figure S21. In vitro assessment of 2′-OMe, 5′-CP, and 5′-CP with PO-replacement in the gap region in ASO2.

Neuro-2a cells were transfected with 2 nM ASO (knockdown activity assessment) (**A**) and BE(2)-M17 cells were transfected with 20 nM ASO (toxicity assessment) (**B-D**), using the following ASOs: ASO2, ASO2 with 2′-OMe at gap position 2, and ASO2 with 5′-CP at gap position 1, 3, or 9 with or without PO-replacement. Target *Hdac2* mRNA levels (**A**), cell number (**B**). LDH release (**C**), and *CDKN1A* mRNA levels (**D**) 72 h post-transfection are presented as mean ± SEM, as a percentage relative to the vehicle (n = 4 per group). # and * indicates significance against vehicle and ASO2, respectively.

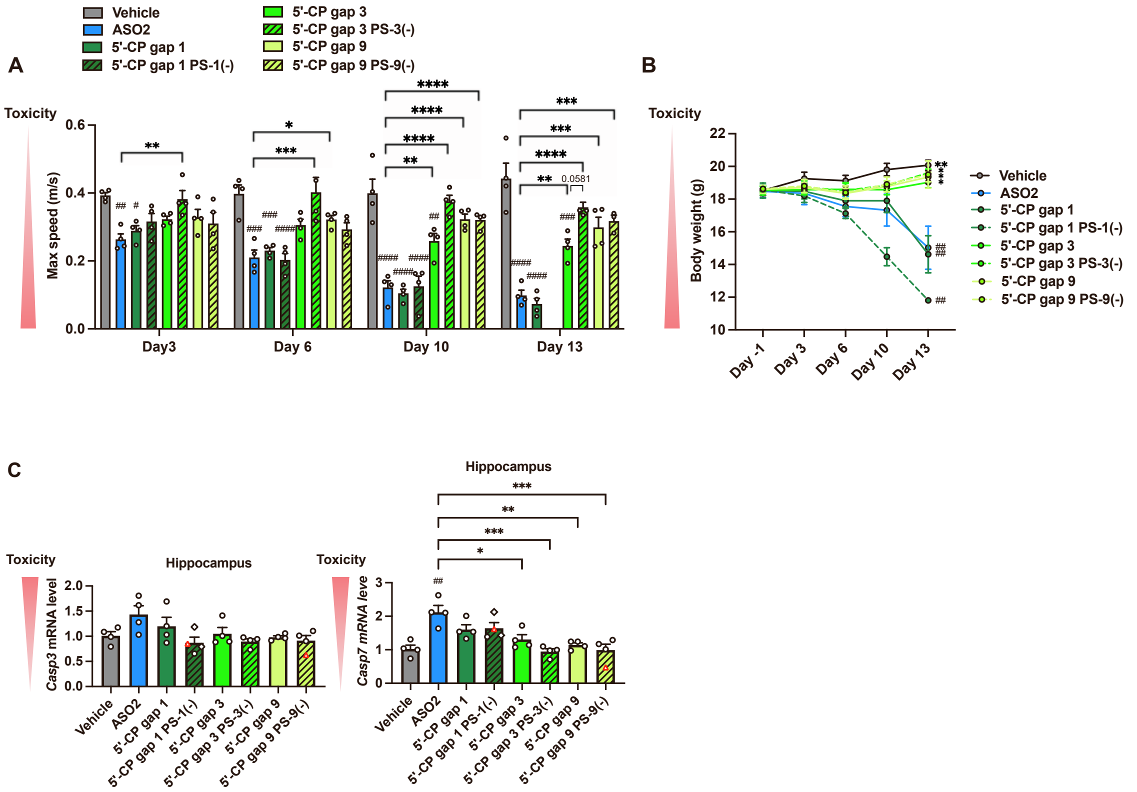

Figure S22. In vivo assessment of ASO2 with 5′-CP at gap positions 1, 3, or 9, with or without PO-replacement via ICV-injection.

Seven-week-old female C57BL/6 mice (n = 4 per group) were injected intracerebroventricularly with 19.0 nmol of the parent ASO2 and ASO2 with 5′-CP at gap positions 1, 3, or 9, with or without PO-replacement. Toxicity was assessed on the indicated days, and mice were sacrificed 14 days postinjection. (**A**) Maximum speed in open-field tests were recorded on the indicated days postinjection. (**B**) Changes in body weight postinjection. (**C**) *Caspase-3* (*Casp3*) and *Caspase-7* (*Casp7*) mRNA levels in the left hippocampus were measured 14 days postinjection. Symbols indicate collection days: rounds (day 14), rhombus (day 10), and red triangles (day 13, post-mortem). Data are presented as mean ± SEM. # and * indicates significance against vehicle and ASO2, respectively.

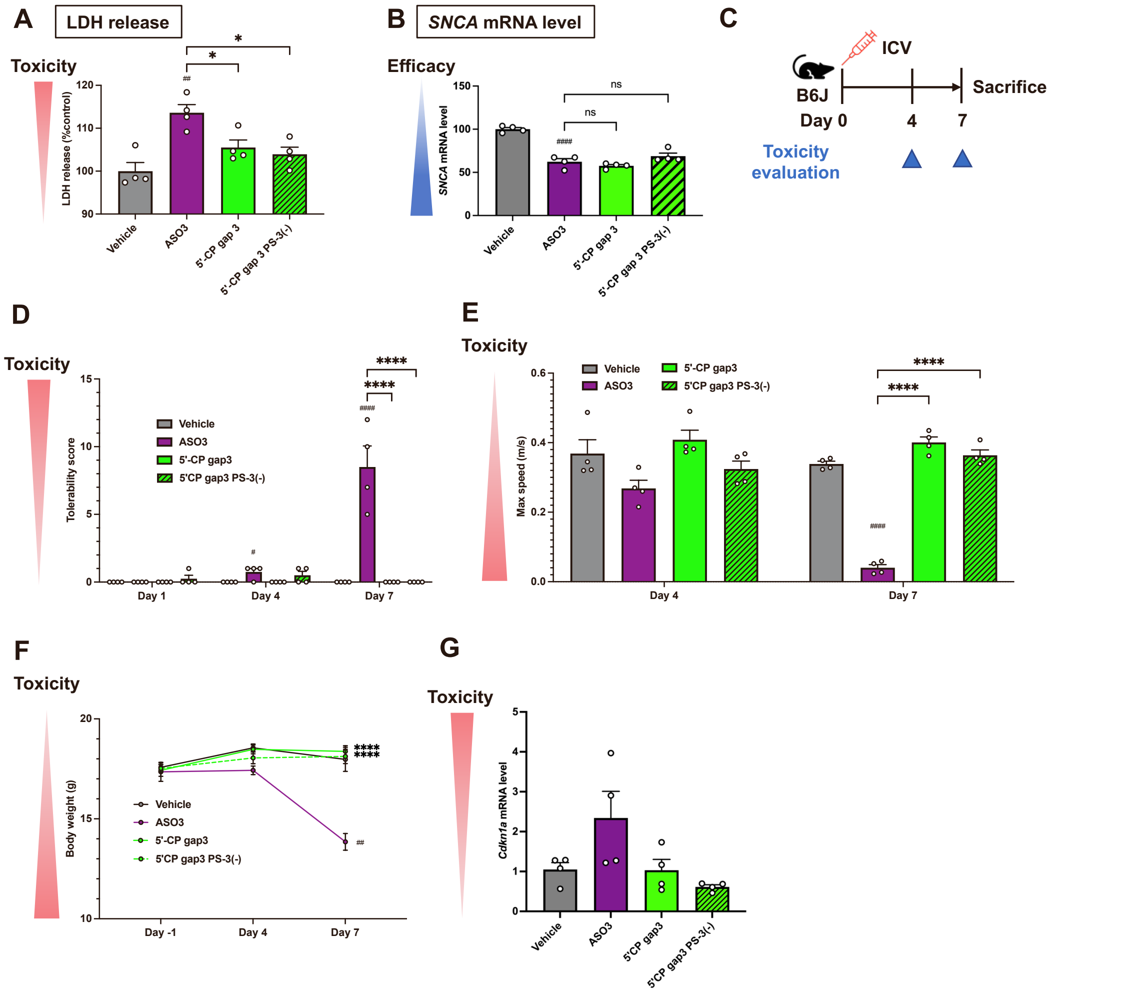

Figure S23. In vitro and in vivo assessment of ASO3 with 5′-CP at gap positions 3 with or without PO-replacement.

(**A, B**) BE(2)-M17 cells were transfected with 50 nM ASO3 (**A**) or 0.4 nM ASO3 (**B**), with 5′-CP at gap positions 3 or 5′-CP at gap positions 3 with PS-3(-). LDH release (**A**) and target mRNA levels (**B**) 72 h post-transfection are shown as mean ± SEM, as a percentage relative to the vehicle. (**C-G**) Seven-week-old female C57BL/6 mice (n = 4 per group) were injected intracerebroventricularly with 15.2 nmol of the parent ASO3 and ASO3 with 5′-CP at gap positions 3 with or without PO-replacement. Tolerability scores (**D**), maximum speed (**E**), change in body weight (**F**) and *Cdkn1a* mRNA levels in the left hippocampus (**G**) were measured. Data are presented as mean ± SEM. $^{\#}p \leq 0.05$, $^{\#\#}p \leq 0.01$, and $^{\#\#\#\#}p \leq 0.0001$; data were analyzed using Student's two-tailed t-tests with vehicle. $^{*}p \leq 0.05$ and ****$p \leq 0.0001$; data were analyzed using one-way ANOVA, followed by Tukey's post hoc tests with ASO3. Ns, not significant ($p > 0.05$).

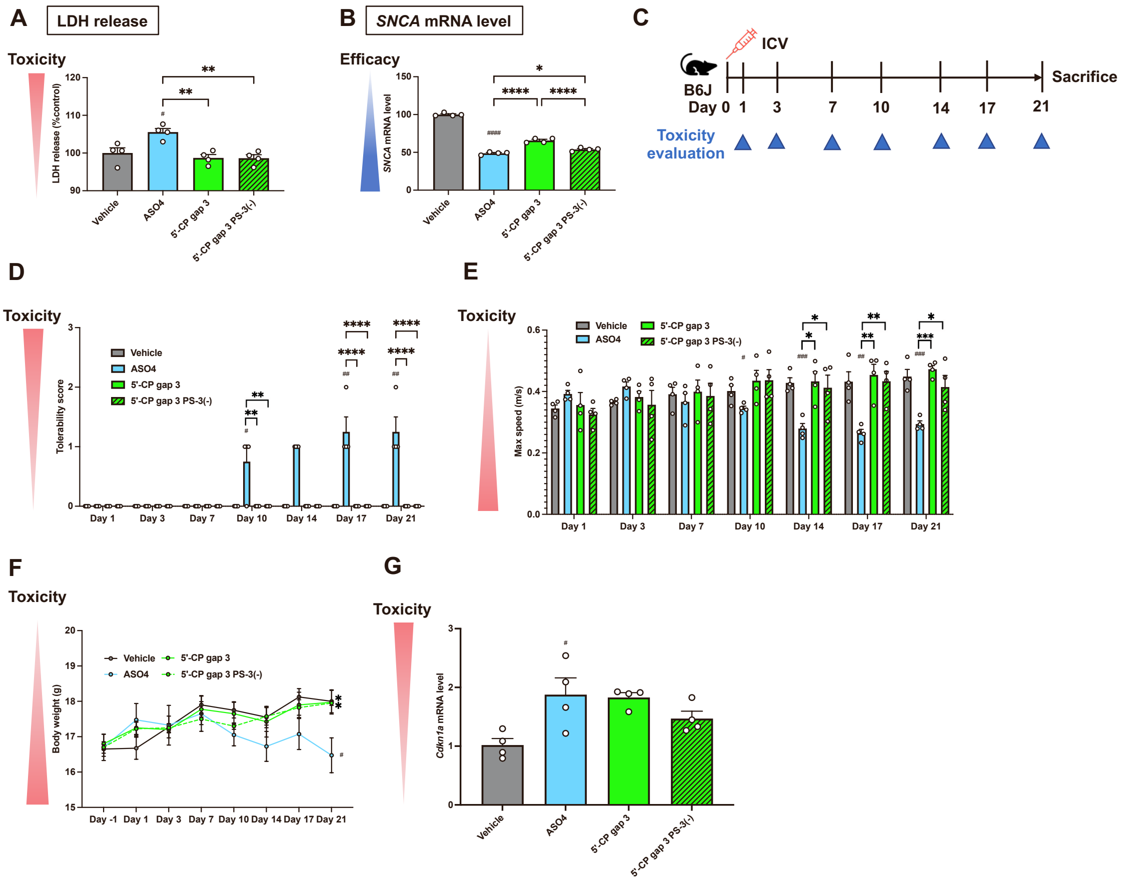

Figure S24. In vitro and in vivo assessment of ASO4 with 5′-CP at gap positions 3 with or without PO-replacement.

(**A, B**) BE(2)-M17 cells were transfected with 50 nM ASO4 (**A**) or 1 nM ASO4 (**B**), with 5′-CP at gap positions 3 or 5′-CP at gap positions 3 with PS-3(-). LDH release (**A**) and target mRNA levels (**B**) 72 h post-transfection are shown as mean ± SEM, as a percentage relative to the vehicle. (**C-G**) Seven-week-old female C57BL/6 mice (n = 4 per group) were injected intracerebroventricularly with 39.9 nmol of the parent ASO4 and ASO4 with 5′-CP at gap positions 3 with or without PO-replacement. Tolerability scores (**D**), maximum speed (**E**), change in body weight (**F**) and *Cdkn1a* mRNA levels in the left hippocampus (**G**) were measured. Data are presented as mean ± SEM. $^{\#}p \leq 0.05$, $^{\#\#}p \leq 0.01$, $^{\#\#\#}p \leq 0.001$, and $^{\#\#\#\#}p \leq 0.0001$; data were analyzed using Student's two-tailed t-tests with vehicle. $^{*}p \leq 0.05$, $^{**}p \leq 0.01$, $^{***}p \leq 0.001$, and $^{****}p \leq 0.0001$; data were analyzed using one-way ANOVA, followed by Tukey's post hoc tests with ASO4, or as indicated.

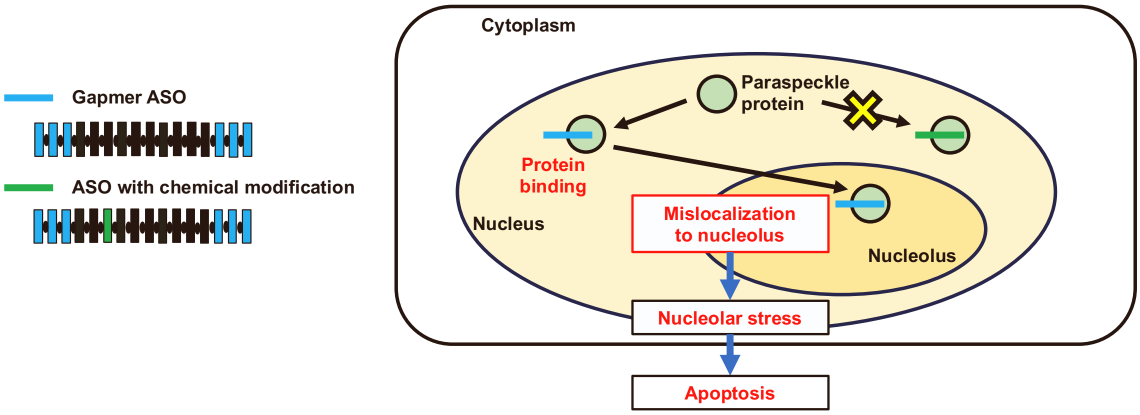

Figure S25. Schematic of PS-ASO-induced cytotoxicity and its prevention by chemical modification.

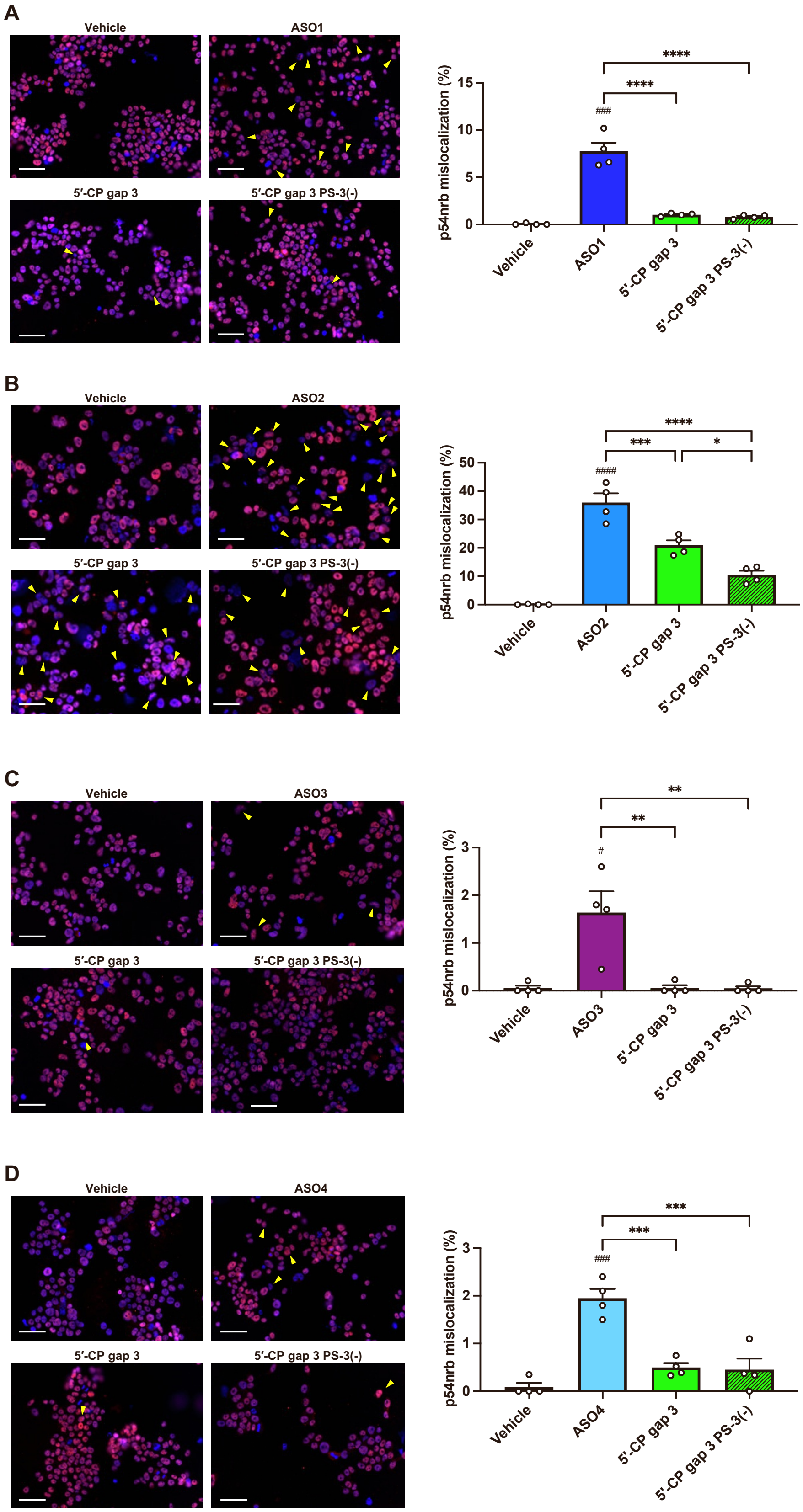
A
Vehicle
ASO1
5′-CP gap 3
5′-CP gap 3 PS-3(-)
p54nrb mislocalization (%)
0
5
10
15
****
****
###
Vehicle
ASO1
5'-CP gap 3
5'-CP gap 3 PS-3(-)
B
Vehicle
ASO2
5′-CP gap 3
5′-CP gap 3 PS-3(-)
p54nrb mislocalization (%)
0
10
20
30
40
50
****
***
*
####
Vehicle
ASO2
5'-CP gap 3
5'-CP gap 3 PS-3(-)
C
Vehicle
ASO3
5′-CP gap 3
5′-CP gap 3 PS-3(-)
p54nrb mislocalization (%)
0
1
2
3
**
**
#
Vehicle
ASO3
5'-CP gap 3
5'-CP gap 3 PS-3(-)
D
Vehicle
ASO4
5′-CP gap 3
5′-CP gap 3 PS-3(-)
p54nrb mislocalization (%)
0
1
2
3
***
***
###
Vehicle
ASO4
5'-CP gap 3
5'-CP gap 3 PS-3(-)

Figure S26. Immunofluorescence study of p54nrb in BE(2)-M17 cells transfected with ASO1-4 modified with 5′-CP at gap position 3, with or without PO-replacement.

BE(2)-M17 cells were transfected for 3 h with 200 nM of ASOs modified with 5′-CP at gap position 3, with or without PO-replacement. Representative microscopic immunostaining images of p54nrb and percentage of p54nrb mislocalization to nucleoli are shown for each ASO: (**A**) ASO1, (**B**) ASO2, (**C**) ASO3, and (**D**) ASO4. Data are presented as mean ± SEM of different microscopic images within the same well (n = 4 per group). $^{\#}p \leq 0.05$, $^{\#\#\#}p \leq 0.001$, and $^{\#\#\#\#}p \leq 0.0001$; data were analyzed using Student's two-tailed t-tests with vehicle. $^{*}p \leq 0.05$, $^{**}p \leq 0.01$, $^{***}p \leq 0.001$, and $^{****}p \leq 0.0001$; data were analyzed using one-way ANOVA, followed by Tukey's post hoc tests. Scale bar, 50 µm.

Videos S1-S3: Videos of mice from the experiment shown in Figures 3E-3H (n=4 per group), injected with either vehicle (Video S1), ASO2 (Video S2), or ASO2 with 2′-OMe modification at gap position 2 (Video S3), recorded 6 days postinjection.

Videos S4-S6: Representative videos of a rat from the experiment shown in Figures 4L and 4M (n=4 per group), injected with either vehicle (Video S4), ASO2 (Video S5), or ASO2 with 5′-CP modification at gap position 3 (Video S6), recorded 14 days postinjection.

Videos S7-S13: Representative videos of two mouse from the experiment shown in Figures 6H-6K (n=4 per group), injected with ASO2 (Video S7), ASO2 with 5′-CP at gap position 1 (Video S8), ASO2 with 5′-CP at gap position 1 with PS-1(-) (Video S9), ASO2 with 5′-CP at gap position 3 (Video S10), ASO2 with 5′-CP at gap position 3 with PS-3(-) (Video S11), ASO2 with 5′-CP at gap position 9 (Video S12), ASO2 with 5′-CP at gap position 9 with PS-9(-) (Video S13), recorded 10 days postinjection.
