# Supplementary material for: Unraveling and controlling late-onset neurotoxicity of antisense oligonucleotides through strategic chemical modifications
Source: Mol Ther Nucleic Acids. 2025 Sep 12;36(4):102692. doi: 10.1016/j.omtn.2025.102692 (PMC12744863; doi:10.1016/j.omtn.2025.102692)
Supplement: Document S14. Article plus supplemental information [file mmc15.pdf]

Molecular Therapy
Nucleic Acids
Original Article

Unraveling and controlling late-onset neurotoxicity of antisense oligonucleotides through strategic chemical modifications

Takayuki Kuroda,[1,2,5] Kotaro Yoshioka,[1,2,3,5] Su Su Lei Mon,[1,2,3,5] Maho Katsuyama,[1,2] Kumiko Sato,[1,2] Eriko Isogai,[1,2] Kie Yoshida-Tanaka,[1,2] Rintaro Iwata-Hara,[1,2] Takao Yamaguchi,[4] Satoshi Obika,[4] and Takanori Yokota[1,2,3]

[1]Department of Neurology and Neurological Science, Graduate School of Medical and Dental Sciences, Institute of Science Tokyo, 1-5-45 Yushima, Bunkyo-Ku, Tokyo 113-8519, Japan; [2]Center for Brain Integration Research, Institute of Science Tokyo, 1-5-45 Yushima, Bunkyo-ku, Tokyo 113-8519, Japan; [3]NucleoTIDE and PepTIDE Drug Discovery Center, Institute of Science Tokyo, 1-5-45 Yushima, Bunkyo-ku, Tokyo 113-8519, Japan; [4]Graduate School of Pharmaceutical Sciences, Osaka University, 1-6 Yamadaoka Suita, Osaka 565-0871, Japan

Antisense oligonucleotides (ASOs) represent an attractive therapeutic approach for CNS disorders. However, ASO-induced neurotoxicity, especially late-onset adverse events, remains a crucial issue, leading to failures in clinical applications. This study aims to determine the neurological features and molecular mechanisms of the late-onset neurotoxicity and provide strategies to overcome this toxicity. We initially established neurobehavioral assays of rodent neurotoxicity with intracerebroventricular and intrathecal injections of various gapmer-type ASOs and a neuronal cytotoxicity analysis. Through both *in vitro* and *in vivo* assessments, we identified a site-specific chemical modification, 5′-cyclopropylene (5′-CP), that significantly reduced late-onset neurotoxicity without compromising knockdown activity, providing useful insights into structure-toxicity and structure-activity relationships in ASOs targeting CNS. Additionally, we revealed a toxicity-related mechanism as an elevation of p53-regulated transcripts and paraspeckle protein mislocalization in neuronal cells, which is alleviated through the chemical modifications. Our findings provide mechanistic insights into late-onset ASO-induced neurotoxicity and highlight the potential of optimized chemical modifications to expand the therapeutic window for clinical applications targeting intractable neurological diseases.

INTRODUCTION

Antisense oligonucleotides (ASOs) are therapeutic molecules that modulate gene expression or splicing by binding to target pre-mRNA through Watson-Crick base pairing.[1] The United States Food and Drug Administration approved numerous ASO therapeutics to treat various diseases over the past decade.[2–5] The central nervous system (CNS) has emerged as a crucial target area for ASO therapies, with two landmark approvals for neurodegenerative diseases: nusinersen for spinal muscular atrophy and tofersen for amyotrophic lateral sclerosis caused by the superoxide dismutase 1 (SOD1) gene mutations.[6,7] ASO technology has also been applied to develop patient-customized therapies for rare and intractable genetic CNS diseases, frequently lacking commercial viability due to their small patient populations.[8–10] Many clinical trials targeting CNS diseases with ASOs are ongoing, but several have been discontinued after failing to meet endpoints.[11]

A major challenge in developing CNS-targeting ASOs is neurotoxicity after intrathecal (i.t.) injection, which limits the tolerable dosing and consequently reduces the therapeutic efficacy.[12,13] Acute-onset neurotoxicity, appearing within 30 min of i.t. injection and resolving within one day, has been reported.[12–15] Previous studies indicate that this toxicity is sequence and chemical modification dependent, potentially involving intracellular calcium homeostasis disruption via glutamate receptors on cell membranes.[12–14,16] In addition to this acute toxicity, late-onset neurotoxicity has recently emerged as a significant concern in clinical trials, including the tofersen trial.[6,17–19] However, *in vitro* and *in vivo* characteristics and molecular mechanisms of this late-onset neurotoxicity, distinct from acute toxicity, remain poorly understood. Furthermore, the need for novel strategies to mitigate late-onset neurotoxicity without compromising the ASO's therapeutic activity remains unmet.

Gapmer-type ASO, including tofersen, represents one of the most promising ASO classes for CNS targeting, providing potent silencing of target RNA through RNase H1-mediated degradation, while

Received 9 January 2025; accepted 20 August 2025;
https://doi.org/10.1016/j.omtn.2025.102692.

[5]These authors contributed equally

Correspondence: Satoshi Obika, Graduate School of Pharmaceutical Sciences, Osaka University, 1-6 Yamadaoka Suita, Osaka 565-0871, Japan.
E-mail: obika@phs.osaka-u.ac.jp
Correspondence: Takanori Yokota, Department of Neurology and Neurological Science, Graduate School of Medical and Dental Sciences, Institute of Science Tokyo, 1-5-45 Yushima, Bunkyo-Ku, Tokyo 113-8519, Japan.
E-mail: tak-yokota.nuro@tmd.ac.jp

© 2025 The Authors.
Published by Elsevier Inc. on behalf of The American Society of Gene and Cell Therapy.
This is an open access article under the CC BY license (http://creativecommons.org/licenses/by/4.0/).

exhibiting variable neurotoxicity tolerance.[6,20–27] Gapmer ASO consists of a central "gap" region and two adjacent "wing" regions flanking the gap. The wing regions comprise chemically modified nucleosides, including 2′-O-methyl RNA (2′-OMe), 2′-*O*-methoxyethyl RNA (2′-MOE), and locked nucleic acid (LNA), increasing binding affinity to the target RNA. The gap region contains DNA to activate RNase H1 for degradation of the target RNA. Internucleoside linkages in gapmer ASOs basically feature phosphorothioate (PS) bonds instead of phosphodiester (PO) bonds, enhancing their nuclease resistance.[28]

Introducing chemical modifications in the gap region of gapmer PS-ASOs modulates ASO-protein binding and mitigates hepatotoxicity in mice after systemic administration.[29–32] For instance, introducing 2′-OMe at the second DNA position from the 5′ end of the gap (position 2)[29] or replacing multiple PS bonds with mesyl-phosphoramidate bonds in the gap[32] reduces hepatotoxicity. Similarly, 5′-modified DNA at specific gap positions, such as S-configured 5′-methyl (Me) DNA at position 3 and R-configured 5′-Me DNA at position 4, mitigates hepatotoxicity.[29,30]

ASO-induced cytotoxicity with PS bonds has been attributed to unintended protein binding by PS modification.[31,33–37] After entering the nucleus, PS-ASOs bind to paraspeckle proteins, delocalizing them to nucleoli and causing nucleolar stress, inducing apoptotic cell death.[29,34,37–39] Whereas this phenomenon has been observed in HeLa cells and mouse hepatocytes,[29] its occurrence in the CNS remains unknown.

This study initially aimed to characterize *in vivo* symptomatic features of CNS dysfunction at the late phases (≥3 days) after intracerebroventricular (i.c.v.) injections into mice as well as i.t. injections into rats with gapmer PS-ASOs, including those that were non-toxic in the acute phase. We next investigated whether site-specific gap modifications could mitigate cytotoxicity using our established *in vitro* assay and an *in vivo* model of late-onset neurotoxicity. For this mitigation, we introduced 2′-OMe and our original 5′-modified DNA, 5′-cyclopropylene (5′-CP).[40] Notably, 5′-CP at certain gap positions significantly improved the late-onset neurotoxicity without reducing knockdown activity, whereas 2′-OMe exacerbated it. Finally, we revealed that mislocalization of the paraspeckle protein, p54nrb, from the nucleoplasm to the nucleolus in neural culture cells is associated with late-onset neurotoxicity. This mislocalization was improved by the gap modifications that mitigated the neurotoxicity *in vivo*, suggesting a potential mechanism for late-onset neurotoxicity and its mitigation.

RESULTS

Neurobehavioral analysis of late-onset CNS toxicity in rodents caused by ASOs with different structures and target genes

To characterize late-onset neurological disturbance induced by PS-ASOs, we designed four gapmer-type PS-ASOs (ASO1–4) with varying lengths and target genes (Table S1).[41–43] The sequences and structures depend on ASOs previously reported to cause intolerable effects in the late phase, more than 2 weeks, after i.c.v. injections into mice.[41–43] We also included fifth ASO (ASO5), non-toxic ASO targeting *HTT* mRNA, already used in the clinical trials, as a reference control.[22,44] To investigate whether the ASOs induce late-"onset" neurotoxicity, distinct from an extension of acute toxicity, we administered these ASOs via the i.c.v. route into C57BL/6 mice or the i.t. route into Slc:SD rats (Figures 1A–1G, S1–S3, and S6). Neurological functions were assessed quantitatively over an extended period using neurological behavior and locomotor analyses, tolerability scoring (Table S2), and the open-field test, previously used to measure ASO-induced neurotoxicity.[14,45] The doses of ASOs used in these tests were the minimum necessary to induce significant neurotoxicity compared with the vehicle, although this threshold may vary depending on the ASO sequence. We set the assessment intervals based on the expected onset and progression of toxicity caused by each parent ASO.

In the tolerability scoring assay, ASO1–4—but not the non-toxic control ASO5—showed increased scores, particularly in motor function and appearance (Figures 1B and S1). For ASO2 and ASO3, impairment was also observed in consciousness. These findings demonstrate that the observed neurotoxicity reflects hypoactivity in motor function and consciousness rather than hyperactivity.

In the open-field test, ASO1–4 showed reduced locomotor activity, consistent with the tolerability scores. Notably, maximum speed (max speed) was decreased with all four ASOs (Figure 1C). Additionally, distance and mobile time were decreased with ASO2 and ASO3 (Figure S2), resulting in more apparent abnormalities in track plots compared with ASO1 and ASO4 (Figure 1D).

ASO2 and ASO3 caused a severe reduction in body weight, whereas ASO1 and ASO4 also induced a significant decrease (Figure S3).

We also confirmed that another mouse strain, Institute of Cancer Research strain (ICR), exhibited late-onset neurotoxicity with ASO1 (tolerability scoring and max speed in open-field tests) and ASO4 (tolerability scoring) (Figures S4 and S5).

In the i.t. injection study, a major method in clinical practice, rats with spinal canal catheters were injected with 190 nmol (1.0 mg) ASO2 (Figure 1E) into the cerebrospinal fluid space around lumbar spinal cord. A tolerability scoring system modified for rats (Table S2) revealed increased scores, including severe paraplegia (Figures 1F and S6A). One of the four rats injected with ASO2 died from severe toxicity on day 14. The open-field test showed decreased max speed and abnormality in track plots (Figures 1G and S6B), along with reduced body weight over time (Figure S6C). These results were consistent with those from the mice experiments. Additionally, increased bladder volume was observed at dissection 14 days postinjection (Figure S6D), indicating urinary dysfunction resulting from damage at the lumbar and sacral spinal cord levels.

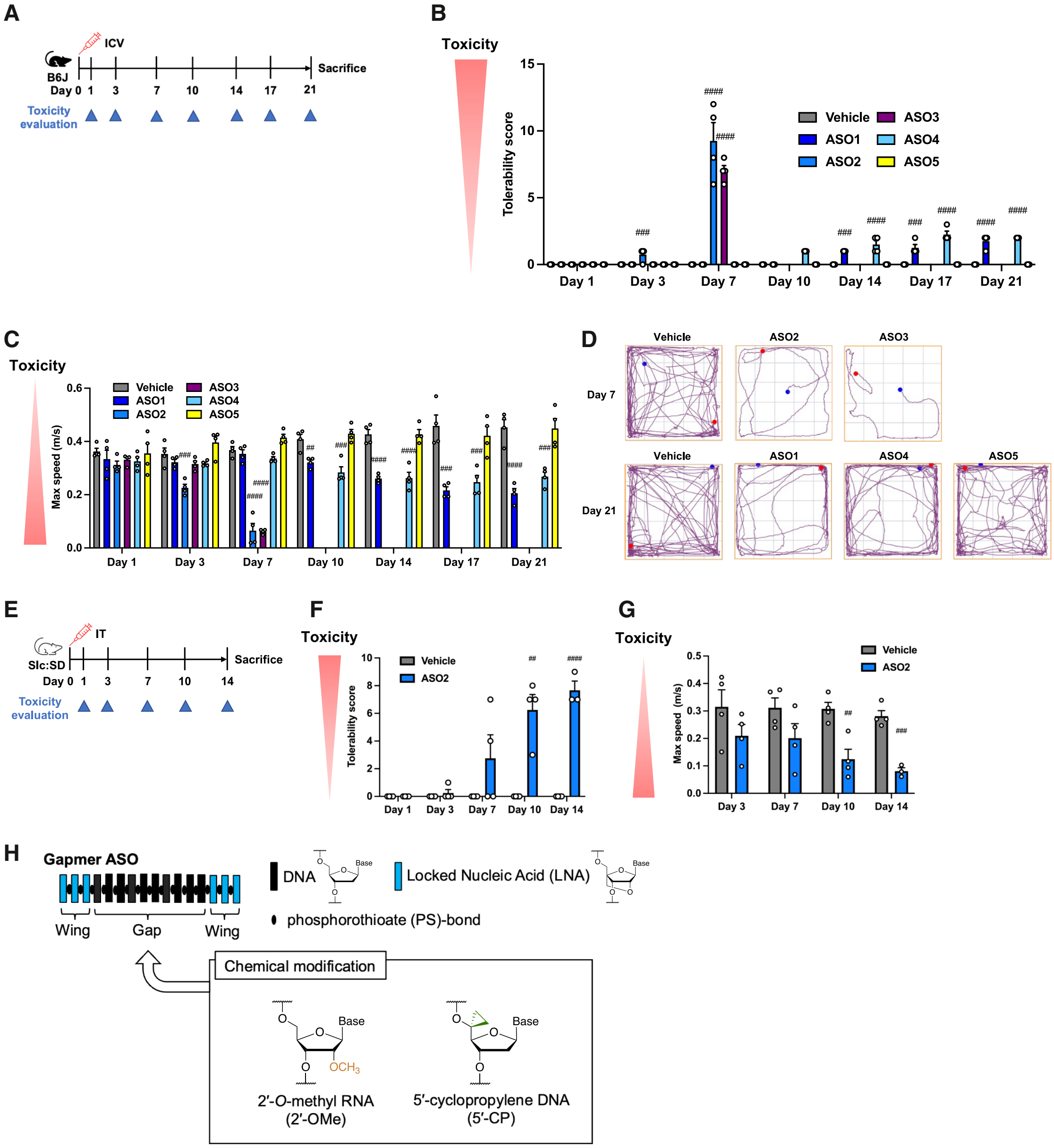

Figure 1. Symptomatic features of late-onset CNS toxicity after i.c.v. and i.t. injections of PS-ASOs

(A–D) Seven-week-old female C57BL/6 mice (n = 4 per group) were injected intracerebroventricularly with ASO1 (38.4 nmol, 200 μg), ASO2 (19.0 nmol, 100 μg), ASO3 (15.2 nmol, 100 μg), ASO4 (39.9 nmol, 250 μg), or ASO5 (39.9 nmol, 280 μg). Toxicity was assessed on the indicated days. ASO5 served as a non-toxic negative control. Mice injected with ASO2 and ASO3 were sacrificed 7 days postinjection; others were sacrificed 21 days postinjection. (B) Tolerability scores over time postinjection. (C) Maximum speed in open-field tests at the indicated time points. (D) Representative track plots in open-field tests at the indicated time points. (E–G) Nine-week-old male Slc:SD rats (n = 4 per group) were injected intrathecally via spinal canal catheters with 190 nmol (1.0 mg) of ASO2. Toxicity was assessed on the indicated days, and rats were sacrificed 14 days postinjection. (F) Tolerability scores over time. (G) Maximum speed in open-field tests. (H) Concept of introducing chemical modifications into the gap region of PS-ASO. Data in (B), (C), (F), and (G) are presented as mean ± SEM. $^{\#\#}p \leq 0.01$, $^{\#\#\#}p \leq 0.001$, and $^{\#\#\#\#}p \leq 0.0001$; data were analyzed using Student's two-tailed t tests (F and G) or two-sided Dunnett's tests (B and C) with vehicle.

These neurological assessments revealed that all four ASOs administered with i.c.v. injections in mice and ASO2 administered with i.t. injection in rats caused *in vivo* neurotoxicity. This toxicity appeared at least 3 days postinjection, persisted for 6 days or more, and worsened over time. Notably, this neurotoxicity at the late phase were characterized as late-"onset," because acute neurotoxicity was not observed with ASO1, ASO3, and ASO4, and acute toxicity from ASO2 resolved completely within 1 day.

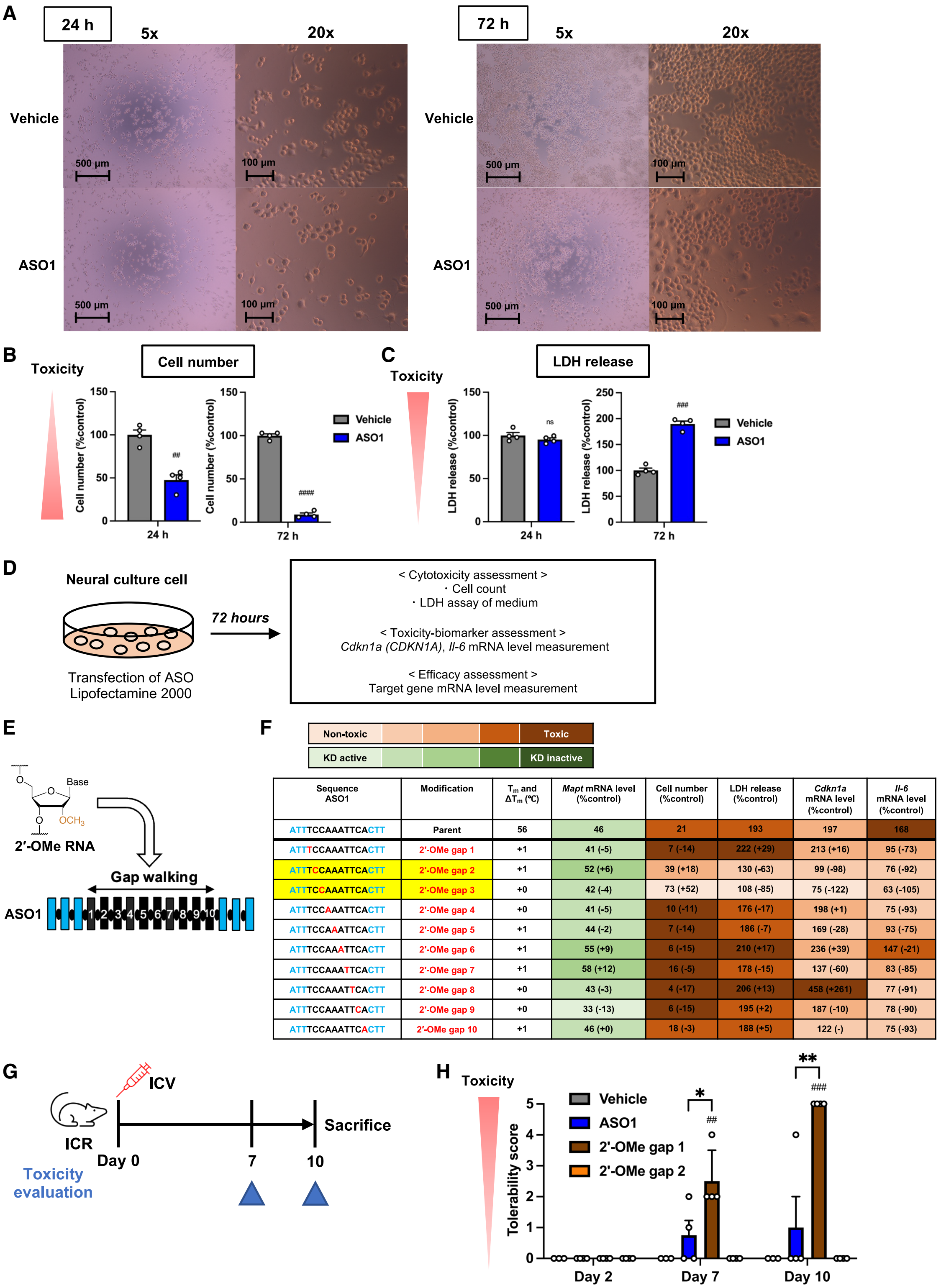

Sequence ASO1	Modification	T_m and ΔT_m (℃)	*Mapt* mRNA level (%control)	Cell number (%control)	LDH release (%control)	*Cdkn1a* mRNA level (%control)	*Il-6* mRNA level (%control)
ATTTCCAAATTCACTT	Parent	56	46	21	193	197	168
ATTTCCAAATTCACTT	2′-OMe gap 1	+1	41 (-5)	7 (-14)	222 (+29)	213 (+16)	95 (-73)
ATTTCCAAATTCACTT	2′-OMe gap 2	+1	52 (+6)	39 (+18)	130 (-63)	99 (-98)	76 (-92)
ATTTCCAAATTCACTT	2′-OMe gap 3	+0	42 (-4)	73 (+52)	108 (-85)	75 (-122)	63 (-105)
ATTTCCAAATTCACTT	2′-OMe gap 4	+0	41 (-5)	10 (-11)	176 (-17)	198 (+1)	75 (-93)
ATTTCCAAATTCACTT	2′-OMe gap 5	+1	44 (-2)	7 (-14)	186 (-7)	169 (-28)	93 (-75)
ATTTCCAAATTCACTT	2′-OMe gap 6	+1	55 (+9)	6 (-15)	210 (+17)	236 (+39)	147 (-21)
ATTTCCAAATTCACTT	2′-OMe gap 7	+1	58 (+12)	16 (-5)	178 (-15)	137 (-60)	83 (-85)
ATTTCCAAATTCACTT	2′-OMe gap 8	+0	43 (-3)	4 (-17)	206 (+13)	458 (+261)	77 (-91)
ATTTCCAAATTCACTT	2′-OMe gap 9	+0	33 (-13)	6 (-15)	195 (+2)	187 (-10)	78 (-90)
ATTTCCAAATTCACTT	2′-OMe gap 10	+1	46 (+0)	18 (-3)	188 (+5)	122 (-)	75 (-93)

(legend on next page)

Using these late-onset neurological toxic ASOs, we next aimed to test the hypothesis that chemical modifications at specific gap positions can reduce the late-onset neurotoxicity of gapmer PS-ASOs (Figure 1H).

2′-OMe at gap position 2 reduces late-onset neurotoxicity of gapmer PS-ASOs

To optimize site-specific chemical modification for mitigating late-onset neurotoxicity, we first established an *in vitro* screening assay to assess neuronal cytotoxicity. Mouse Neuro-2a or human BE(2)-M17 neuronal cells treated with ASO1 or ASO4, respectively, exhibited abnormal morphology, reduced cell numbers, and elevated lactate dehydrogenase (LDH) release, with these effects becoming pronounced at 72 h post-transfection (Figures 2A–2C and S7).

Using this *in vitro* assay, we subsequently evaluated the cytotoxicity, RNA-binding affinity (as melting temperature, T_m), and knockdown efficiency of ASO1 and ASO4 with single DNA substitutions by 2′-OMe at different positions within the gap regions (gap walking) (Figures 2D–2F, S8, and S9). ASO1 and ASO4 were selected because they differ in sequence length, allowing us to assess whether the effect of chemical modifications depends on sequence length. Additionally, we assessed *Cyclin-dependent kinase inhibitor 1A* (*Cdkn1a* and *CDKN1A)* and *interleukin-6* (*Il-6*) mRNA levels at 72 h post-transfection as toxicity biomarkers to monitor p53 activation and immunogenic response, respectively. ASO doses for knockdown assessment were set to achieve around 50% target mRNA reduction by the parent ASOs. Among the gap positions, the substitution of 2′-OMe modification at position 2 and 3 reduced ASO1 cytotoxicity without significantly decreasing its knockdown efficacy and binding affinity, whereas 2′-OMe substitution at position 2 and 4 mitigated ASO4 cytotoxicity with a moderate reduction in knockdown activity.

Given that 2′-OMe at gap position 2 reduced cytotoxicity in both sequences, we further investigated its *in vivo* effects, alongside 2′-OMe at position 1—which increased the cytotoxicity of ASO1 and served as a positive control—on the late-onset ASO1 toxicity (Figures 2G, 2H, and S10). Consistent with the *in vitro* results, 2′-OMe at position 2 tended to alleviate the late-onset neurotoxicity by the parent ASO1 after i.c.v. injection into mice. Conversely, the 2′-OMe at position 1 significantly exacerbated neurotoxicity.

Increase in late-onset neurotoxicity with 2′-OMe at gap position 2

We further investigated the effect of 2′-OMe at gap position 2 on cytotoxicity and *in vivo* neurotoxicity of ASO2. Contrary to the previous results, 2′-OMe at gap position 2 worsened cytotoxicity (Figures 3A–3D) and late-onset neurotoxicity after i.c.v. injection into mice (Figures 3E–3H; Videos S1, S2, and S3). These findings demonstrated that 2′-OMe modification at gap position 2 can increase late-onset neurotoxicity of PS-ASO in a sequence-dependent manner, suggesting the need for alternative chemical modifications to mitigate this ASO's neurotoxicity.

Gap modification of 5′-CP mitigates cytotoxicity and *in vivo* neurotoxicity across various PS-ASOs

We then developed 5′-CP, our original chemical modification at 5′-site of DNA, and introduced this modification at each site of the gap region, conducting a gap walking study of ASO1 and ASO4 (Figures 4A, 4B, S11, and S12). For ASO1, 5′-CP at gap positions 1, 3, 6, and 9 significantly reduced cytotoxicity, with position 3 being the least toxic. However, these 5′-CP introductions decreased both T_m and knockdown activity. For ASO4, 5′-CP at gap positions 1, 2, 3, 7, 8, 9, and 12 reduced cytotoxicity, with a moderate knockdown activity reduction.

The gap walking study revealed that the gap position 3 was optimal for mitigating cytotoxicity in neuronal cells for ASO1. Although this modification reduced knockdown activity *in vitro*, we prioritized toxicity mitigation and therefore further investigated its effect on late-onset neurotoxicity *in vivo* after i.c.v. injection into mice (Figures 4C–4E and S13). Consistent with the *in vitro* results, 5′-CP modification at position 3 mitigated late-onset neurotoxicity of the parent ASO1, as demonstrated by improved tolerability scores and increased max speed in the open-field test (Figures 4D and S13). However, 5′-CP at position 3 reduced knockdown activity (Figure 4E).

To mitigate ASO2 neurotoxicity, we examined the effects of 5′-CP at gap position 3 in ASO2 (Figures 4F–4J and S14). Gap position 3 was

Figure 2. 2′-OMe modification at gap position 2 mitigates late-onset neurotoxicity of i.c.v.-injected ASO1

(A–C) Neuro-2a cells were transfected with 50 nM ASO1. (A) Representative microscopic images of cells 24 and 72 h after transfection. (B) Cell number 24 and 72 h post-transfection. (C) LDH release in medium 24 and 72 h post-transfection. Data in (B) and (C) are presented as mean ± SEM, as a percentage relative to vehicle (Lipofectamine 2000 with PBS) (*n* = 4 biological replicates per group). ns, not significant ($p > 0.05$). $^{##}p \leq 0.01$, $^{###}p \leq 0.001$, and $^{####}p \leq 0.0001$; data were analyzed using Student's two-tailed t tests with vehicle. (D) Schematic of the *in vitro* experiment and assessment workflow. (E) Schematic of the *in vitro* experiment introducing 2′-OMe RNA at each gap position (gap walking) in ASO1. (F) Neuro-2a cells were transfected with 4 nM ASO1 (knockdown activity assessment) or 50 nM ASO1 (toxicity assessment), with or without 2′-OMe modification at each gap position. KD, knockdown. Blue letters indicate LNA, whereas blue "C" indicates LNA with 5-methylcytosine. Black letters indicate DNA, and red letters indicate 2′-OMe-modified DNA. ΔT_m indicates the T_m of the sequence minus the T_m of the parent ASO. Target mRNA levels, cell number, LDH release, *Cdkn1a* mRNA levels, and *Il-6* mRNA levels 72 h post-transfection are shown as the mean percentage relative to vehicle (*n* = 2 biological replicates per group for *Il-6* mRNA of ASO1 with 2′-OMe at gap position 4; *n* = 4 for others). The highlighted sequences with 2′-OMe modifications show an increase in cell number and a decrease in LDH release compared to the parent ASOs. See also Figure S8. (G and H) Seven-week-old female ICR mice (*n* = 3 per group for the vehicle and *n* = 4 for all other groups) were injected intracerebroventricularly with 38.4 nmol of ASO1 and ASO1 modified with 2′-OMe at gap position 1 or 2. Toxicity was assessed on the indicated days, and mice were sacrificed 10 days postinjection. (H) Tolerability scores were measured at the indicated time points, and data are presented as mean ± SEM. $^{##}p \leq 0.01$ and $^{###}p \leq 0.001$; data were analyzed using Student's two-tailed t tests with vehicle. $^{*}p \leq 0.05$ and $^{**}p \leq 0.01$; data were analyzed using one-way ANOVA, followed by Tukey's *post hoc* tests.

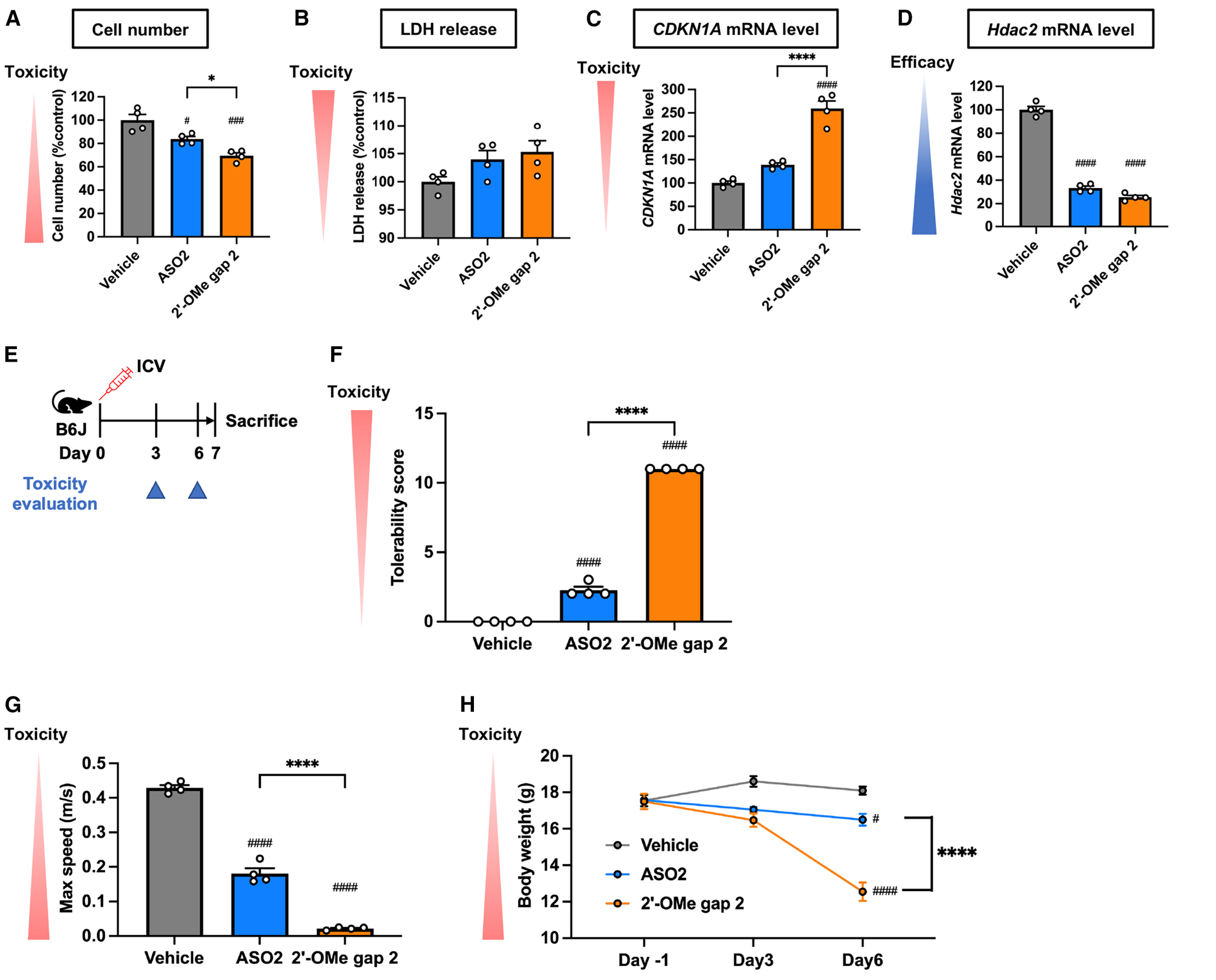

Figure 3. 2′-OMe at gap position 2 increases late-onset neurotoxicity of i.c.v.-injected ASO2

(A–C) BE(2)-M17 cells were transfected with 20 nM ASO2, with or without 2′-OMe modification at gap position 2 Cell number (A), LDH release in medium (B), and *CDKN1A* mRNA levels (C) are shown as the mean percentage relative to vehicle (n = 4 biological replicates per group). (D) Neuro-2a cells were transfected with 2 nM ASO2, with or without 2′-OMe modification at gap position 2. Target *Hdac2* mRNA levels 72 h post-transfection are shown as the mean percentage relative to vehicle (n = 4 biological replicates per group). (E–H) Seven-week-old female C57BL/6 mice (n = 4 per group) were injected intracerebroventricularly with 19.0 nmol of ASO2, with or without 2′-OMe modification at gap position 2. Toxicity was assessed on the indicated days, and mice were sacrificed 7 days postinjection. Tolerability scores (F) and maximum speed in open-field tests (G) were documented 6 days postinjection. Body weight was measured at 3 and 6 days postinjection (H). Data in (A)–(D) and (F)–(H) are presented as mean ± SEM. $^{\#}p \leq 0.05$, $^{\#\#\#}p \leq 0.001$, and $^{\#\#\#\#}p \leq 0.0001$; data were analyzed using Student's two-tailed t tests with vehicle. $^{*}p \leq 0.05$ and $^{****}p \leq 0.0001$; data were analyzed using one-way ANOVA, followed by Tukey's *post hoc* tests.

chosen based on its minimal cytotoxicity in ASO1 and its cytotoxicity-reducing effect in ASO4. Mice were sacrificed when they reached a tolerability score ≥11 and could no longer drink water independently, in accordance with ethical guidelines. As a result, sacrifice time points varied in the parent ASO2 group (days 8, 9, and 11), whereas mice in the vehicle and the ASO2 5′-CP at gap position 3 groups were sacrificed on day 11. Surprisingly, 5′-CP modification at position 3 reduced ASO2's late-onset neurotoxicity, as evidenced by improvements in tolerability scores, max speed, and body weight (Figures 4G, 4H, and S14), without significantly reducing knockdown activity (Figure 4I). Elevated mRNA levels of *Cdkn1a*, induced by the parent ASO2, were not induced by the 5′-CP modification (Figure 4J). To determine whether the 5′-CP modification affected the delivery capability of ASOs to CNS tissues, fluorescence-labeled ASOs were injected via i.c.v. route into mice (Figure 4K). ASO2 with 5′-CP at gap position 3 showed no less accumulation in the brain

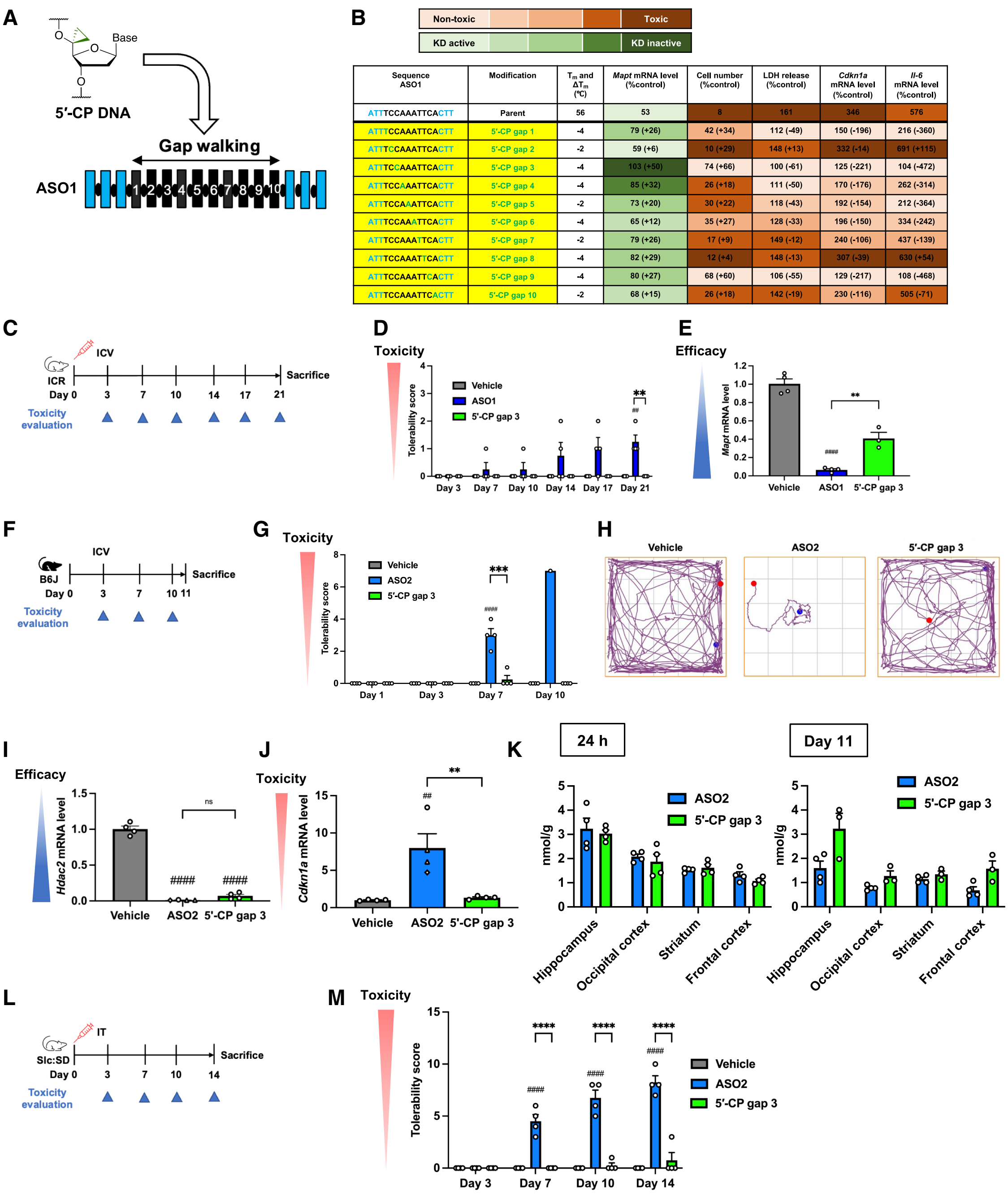

Figure 4. 5′-CP modification in gap mitigates neurotoxicity of both ASO1 and ASO2

(A) Schematic of introducing 5′-CP DNA at each gap position in ASO1. (B) Neuro-2a cells were transfected with 4 or 50 nM ASO1, with or without 5′-CP at each gap position. Green letters indicate 5′-CP-modified DNA. $n = 3$ biological replicates per group for *Cdkn1a* and *Il-6* mRNA of ASO1 with 5′-CP gap positions 4 and 10; $n = 4$ for others. See also Figure S11. (C–E) Seven-week-old female ICR mice were injected intracerebroventricularly with 38.4 nmol of ASO1, with or without 5′-CP position 3 ($n = 3$ for ASO1 with 5′-CP position 3; $n = 4$ for others) and sacrificed 21 days postinjection. Tolerability scores at the indicated times (D) and left hippocampal *Mapt* mRNA levels at 21 days postinjection (E) were measured. (F–J) Seven-week-old female C57BL/6 mice ($n = 4$) were injected intracerebroventricularly with 19.0 nmol of ASO2, with or without 5′-CP position 3, and sacrificed 11 days postinjection. (G) Tolerability scores were recorded. (H) Representative track plots 10 days postinjection. (I and J) Target *Hdac2* and *Cdkn1a* mRNA levels in the left hippocampus were measured 8–11 days postinjection, with round, triangle, and rhombus symbols indicating measurements at days 11, 9, and 8, respectively. (K) Seven-week-old female C57BL/6 mice ($n = 3$–4 per group) were injected intracerebroventricularly with 9.49 nmol of ASO2, with or without 5′-CP position 3, labeled with Alexa Fluor 647 on the 3′ end. ASO delivery was assessed by measuring fluorescence intensity across brain regions 24 h and 11 days postinjection. (L and M)

(legend continued on next page)

than the parent ASO2 at both 24 h and 11 days postinjection, suggesting that the improved tolerability of the 5′-CP modified ASO2 was not due to impaired delivery.

Furthermore, in an i.t.-injection study, ASO2 with 5′-CP at position 3 significantly reduced tolerability scores and bladder volume at sacrifice and improved body weight, indicating mitigation of late-onset neurotoxicity (Figure 4L, 4M; Figures S15B and S17C; Videos S4, S5, and S6). This modification also showed a non-significant trend toward improved max speed in the open-field tests. (Figure S15A). Histopathological analysis of the lumbar cord, cerebellum, and hippocampus revealed no neuronal cell death or inflammation for either ASO, indicating that severe neurodegeneration or gliosis cannot explain *in vivo* late-onset neurological dysfunction by ASO2 and its mitigation by the 5′-CP modification (Figure S16).

These findings indicate that site-specific 5′-CP modification mitigates late-onset neurotoxicity *in vitro* and *in vivo* for various PS-ASOs compared with 2′-OMe, via both i.c.v. and i.t. routes. A modest reduction in knockdown activity of ASO1 by 5′-CP was observed with i.c.v. injection, whereas knockdown activity of ASO2 was maintained by 5′-CP.

PS-bond replacement with a PO bond to mitigate cytotoxicity and maintain knockdown activity

Although PS bonds confer nuclease resistance and help maintain ASO stability, they have also been shown to interact with various proteins, contributing to cytotoxicity.[29,31] In addition, PS bonds can reduce the binding affinity of ASOs to their target RNA.[46] Previous studies have reported that partial substitution of the PS bond with a PO bond in ASOs can reduce toxicity, while maintaining efficacy or enhancing binding affinity.[12,47,48] Based on these observations, we hypothesized that combining PO replacement with 5′-CP modification—which enhances nuclease resistance—could compensate for the reduced stability associated with PO replacement alone. As an initial test, we replaced the PS bond with a PO bond in the gap region, specifically at the 5′-adjacent site of the DNA monomer, positions 1, 3, and 9 (PS-1(−), PS-3(−), and PS-9(−)), to investigate whether these substitutions reduced cytotoxicity. These positions were chosen because they were the most effective sites for 5′-CP to reduce cytotoxicity in ASO1, and they also showed a reduction in cytotoxicity in ASO4. *In vitro* assay, PO replacements at these positions in ASO2 unexpectedly increased cytotoxicity compared with the parent ASO2 (Figures 5A, 5B, and S17). Consistently, *in vivo* study demonstrated higher mortality from ASO2 with PS-1(−), PS-3(−), and PS-9(−) during the experimental period (Figure 5C). Specifically, two of the five mice in the PS-1(−) group, one of the five mice in the PS-3(−) group, and three of the five mice in the PS-9(−) group either succumbed to severe toxicity or were euthanized between 2 and 9 days postinjection. Surviving mice injected with ASO2 PS-3(−) exhibited reduced late-onset neurotoxicity but also showed a significant decrease in knockdown activity (Figures 5D–5G and S18). These findings suggest that merely replacing the PS bond with a PO bond cannot reduce the ASO's cytotoxicity and late-onset neurotoxicity.

The 5′-CP gap modification allows PS to PO replacement, reducing late-onset neurotoxicity and maintaining knockdown activity in the CNS

We next investigated the combination of PO replacement and 5′-CP via *in vitro* screening assays (Figure 6A), hypothesizing that 5′-CP would maintain stability and knockdown activity. We replaced each nucleotide in the gap region of ASO1 with 5′-CP and the 5′-adjacent PS bond with PO bond, ranging from 5′-CP at gap position 1 PS-1(−) to 5′-CP at gap position 10 PS-10(−). This gap walking revealed that this combination at positions 1, 3, 4, and 5 reduced cytotoxicity and toxicity-biomarker levels, including *Cdkn1a* and *Il-6* mRNA, without significantly decreasing its knockdown efficacy (Figures 6B and S19). 5′-CP with PO replacement decreased T_m values less than 5′-CP with PS bond in most positions, suggesting that PO replacement helped maintain binding affinity.

For *in vivo* assays with i.c.v. injection into mice, the parent ASO1 and ASO1 modified with 5′-CP at position 3 with PS-3(−) were injected (Figures 6C–6E and S20). 5′-CP at position 3 with PS-3(−) reduced late-onset neurotoxicity (Figures 6D and S20) and maintained knockdown activity in the hippocampus and lumbar cord compared to the parent ASO1 (Figure 6E). These findings, along with the activity reduction observed for 5′-CP with all PS bonds, suggest that 5′-CP with the PO replacement is suitable for mitigating the neurotoxicity without reducing its activity.

We further introduced 5′-CP at gap positions 1, 3, or 9 with or without the PO replacement in ASO2 (Figure 6F) because 5′-CP without PO replacement at these positions had shown the most effective reduction in cytotoxicity in ASO1 and also demonstrated cytotoxicity reduction in ASO4. As a result, 5′-CP at position 3, 5′-CP at position 3 with PS-3(−), 5′-CP at position 9, and 5′-CP at position 9 with PS-9(−) exhibited higher cell viability and lower LDH release compared with the parent ASO2, indicating a trend toward reduced cytotoxicity (Figures 6G and S21). The knockdown activity was maintained or slightly decreased with modified ASOs. *In vivo* assays with i.c.v. injection into mice evaluated these modifications for late-onset neurotoxicity and knockdown activity in the CNS (Figures 6H–6K; Figure S22; Videos S7, S8, S9, S10, S11, S12, and S13). Some of the mice were sacrificed due to severe toxicity or found dead before 14 days postinjection: two of the four mice in the 5′-CP at position 1 group, all four mice in the

Nine-week-old male Slc:SD rats (n = 4) were injected intrathecally via spinal canal catheters with 190 nmol of ASO2, with or without 5′-CP position 3, and sacrificed 14 days postinjection. (M) Tolerability scores were documented postinjection. Data in (D), (E), (G), (I), (J), (K), and (M) are presented as mean ± SEM. $^{\#\#}p \leq 0.01$ and $^{\#\#\#\#}p \leq 0.0001$; data were analyzed using Student's two-tailed t tests with vehicle. ns, not significant ($p > 0.05$). $^{**}p \leq 0.01$, $^{***}p \leq 0.001$, and $^{****}p \leq 0.0001$; data were analyzed using one-way ANOVA, followed by Tukey's *post hoc* tests.

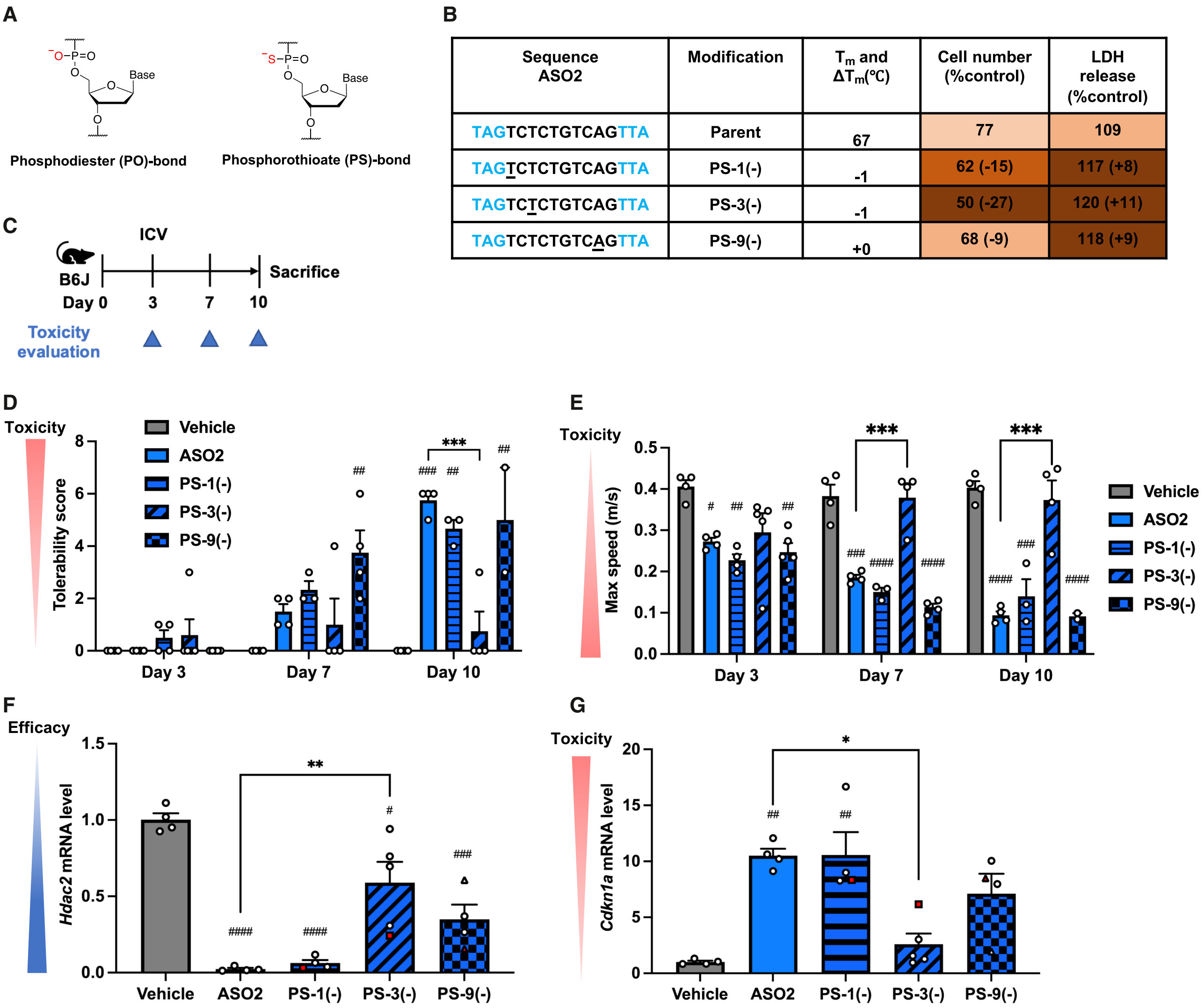

Sequence ASO2	Modification	T_m and ΔT_m(℃)	Cell number (%control)	LDH release (%control)
TAGTCTCTGTCAGTTA	Parent	67	77	109
TAG<u>T</u>CTCTGTCAGTTA	PS-1(-)	-1	62 (-15)	117 (+8)
TAGTC<u>T</u>CTGTCAGTTA	PS-3(-)	-1	50 (-27)	120 (+11)
TAGTCTCTGTC<u>A</u>GTTA	PS-9(-)	+0	68 (-9)	118 (+9)

Figure 5. *In vitro* and *in vivo* neurotoxicity evaluation of PS-ASOs with PO replacement

(A) Structures of a phosphodiester (PO) bond and a phosphorothioate (PS) bond. (B) BE(2)-M17 cells were transfected with 20 nM ASO2 with or without replacement of PS bond 5′-adjacent to a DNA monomer at gap positions 1, 3, or 9 with a PO bond (PS-1(−), PS-3(−), or PS-9(−)). Cell number and LDH release 72 h post-transfection are shown as the mean percentage relative to vehicle ($n = 4$ biological replicates per group). Underlined letters indicate substitution of a PS bond with a PO bond at the 5′-adjacent site of the DNA monomer. See also Figure S17. (C–G) Seven-week-old female C57BL/6 mice were injected intracerebroventricularly with 19.0 nmol of ASO2 or ASO2 with PS-1(−), PS-3(−), or PS-9(−) ($n = 4$ per group for vehicle and the parent ASO2; $n = 5$ per group for the others). Toxicity was assessed on the indicated days, and mice were sacrificed 10 days postinjection. Tolerability scores (D) and maximum speed in open-field tests (E) were documented postinjection. Target *Hdac2* and *Cdkn1a* mRNA levels in the left hippocampus were measured 6–10 days postinjection (F and G). White round and triangle symbols represent measurements taken on days 10 and 9 postinjection, respectively. Red triangle and square symbols represent samples taken postmortem on days 9 and 6 postinjection, respectively. For (F) and (G), $n = 5$ per group for ASO2 PS-3(−); $n = 4$ per group for others. Data in (D)–(G) are presented as mean ± SEM. $^{\#}p \leq 0.05$, $^{\#\#}p \leq 0.01$, $^{\#\#\#}p \leq 0.001$, and $^{\#\#\#\#}p \leq 0.0001$; data were analyzed using Student's two-tailed t tests with vehicle. $^{*}p \leq 0.05$, $^{**}p \leq 0.01$, and $^{***}p \leq 0.001$; data were analyzed using one-way ANOVA, followed by Tukey's *post hoc* tests.

5′-CP at position 1 with PS-1(−) group, and one of the four mice in the 5′-CP at position 9 with PS-9(−) group. 5′-CP at position 3, 5′-CP at position 3 with PS-3(−), 5′-CP at position 9, and 5′-CP at position 9 with PS-9(−) reduced late-onset neurotoxicity without significantly decreasing knockdown activity (Figures 6I, 6J, S22A, and S22B). Some of these modifications increased *Cdkn1a* mRNA levels (Figure 6K). To further evaluate the involvement of apoptosis, we measured *Caspase-3* (*Casp3*) and *Caspase-7* (*Casp7*) mRNA levels (Figure S22C). ASO2 tended to increase *Casp3* and significantly upregulated *Casp7*, indicating apoptotic induction. In contrast, 5′-CP-modified ASOs that reduced late-onset neurotoxicity did not elevate *Casp3* or *Casp7*, indicating that toxicity mitigation effect of 5′-CP can be explained partially by improving induction of apoptosis.

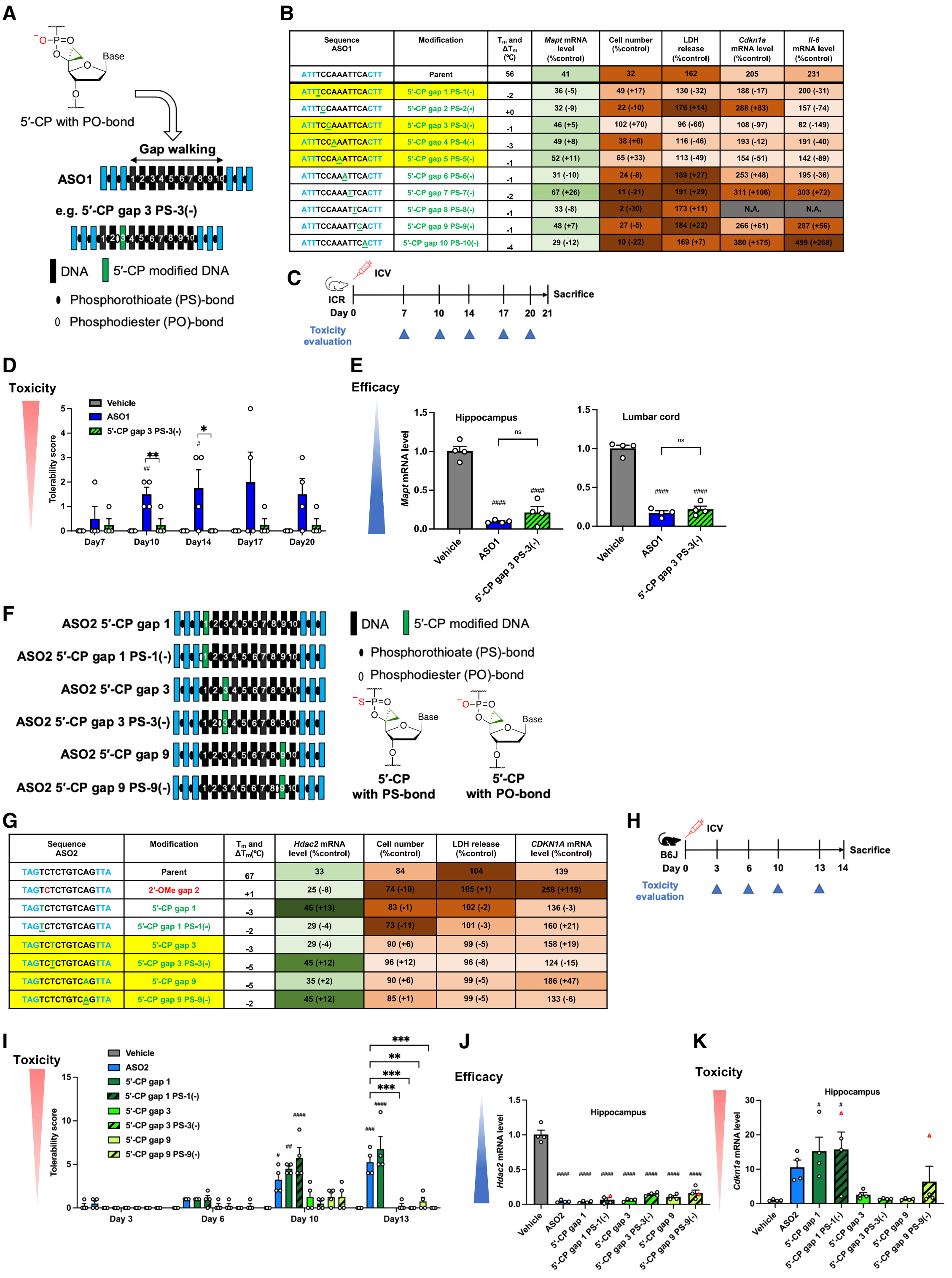

Sequence ASO1	Modification	T_m and ΔT_m (℃)	*Mapt* mRNA level (%control)	Cell number (%control)	LDH release (%control)	*Cdkn1a* mRNA level (%control)	*Il-6* mRNA level (%control)
ATTTCCAAATTCACTT	Parent	56	41	32	162	205	231
ATTTCCAAATTCACTT	5′-CP gap 1 PS-1(-)	-2	36 (-5)	49 (+17)	130 (-32)	188 (-17)	200 (-31)
ATTTCCAAATTCACTT	5′-CP gap 2 PS-2(-)	+0	32 (-9)	22 (-10)	176 (+14)	288 (+83)	157 (-74)
ATTTCCAAATTCACTT	5′-CP gap 3 PS-3(-)	-1	46 (+5)	102 (+70)	96 (-66)	108 (-97)	82 (-149)
ATTTCCAAATTCACTT	5′-CP gap 4 PS-4(-)	-3	49 (+8)	38 (+6)	116 (-46)	193 (-12)	191 (-40)
ATTTCCAAATTCACTT	5′-CP gap 5 PS-5(-)	-1	52 (+11)	65 (+33)	113 (-49)	154 (-51)	142 (-89)
ATTTCCAAATTCACTT	5′-CP gap 6 PS-6(-)	-1	31 (-10)	24 (-8)	189 (+27)	253 (+48)	195 (-36)
ATTTCCAAATTCACTT	5′-CP gap 7 PS-7(-)	-2	67 (+26)	11 (-21)	191 (+29)	311 (+106)	303 (+72)
ATTTCCAAATTCACTT	5′-CP gap 8 PS-8(-)	-1	33 (-8)	2 (-30)	173 (+11)	N.A.	N.A.
ATTTCCAAATTCACTT	5′-CP gap 9 PS-9(-)	-1	48 (+7)	27 (-5)	184 (+22)	266 (+61)	287 (+56)
ATTTCCAAATTCACTT	5′-CP gap 10 PS-10(-)	-4	29 (-12)	10 (-22)	169 (+7)	380 (+175)	499 (+268)

Sequence ASO2	Modification	T_m and ΔT_m(℃)	*Hdac2* mRNA level (%control)	Cell number (%control)	LDH release (%control)	*CDKN1A* mRNA level (%control)
TAGTCTCTGTCAGTTA	Parent	67	33	84	104	139
TAGTCTCTGTCAGTTA	2′-OMe gap 2	+1	25 (-8)	74 (-10)	105 (+1)	258 (+119)
TAGTCTCTGTCAGTTA	5′-CP gap 1	-3	46 (+13)	83 (-1)	102 (-2)	136 (-3)
TAGTCTCTGTCAGTTA	5′-CP gap 1 PS-1(-)	-2	29 (-4)	73 (-11)	101 (-3)	160 (+21)
TAGTCTCTGTCAGTTA	5′-CP gap 3	-3	29 (-4)	90 (+6)	99 (-5)	158 (+19)
TAGTCTCTGTCAGTTA	5′-CP gap 3 PS-3(-)	-5	45 (+12)	96 (+12)	96 (-8)	124 (-15)
TAGTCTCTGTCAGTTA	5′-CP gap 9	-5	35 (+2)	90 (+6)	99 (-5)	186 (+47)
TAGTCTCTGTCAGTTA	5′-CP gap 9 PS-9(-)	-2	45 (+12)	85 (+1)	99 (-5)	133 (-6)

Figure 6. 5′-CP modification with PO replacement mitigates late-onset neurotoxicity of i.c.v.-injected PS-ASOs without reducing knockdown activity

(A) Schematic of introducing DNA monomers with 5′-CP modification and PO replacement of the 5′-adjacent PS bond at each ASO1 gap position. (B) Neuro-2a cells were transfected with 4 or 50 nM ASO1, with or without replacement of 5′-CP with PO replacement. $n = 3$ biological replicates per group for cell number of vehicle and ASO1; $n = 4$ for others. N.A., not applicable due to insufficient RNA. See also Figure S19. (C–E) Seven-week-old female ICR mice ($n = 4$) were injected intracerebroventricularly with

(legend continued on next page)

We additionally conducted both *in vitro* and *in vivo* experiments using ASO3 and ASO4 with or without modifications: 5′-CP at position 3 and 5′-CP at position 3 with PS-3(−) (Figures S23 and S24). These modifications reduced the late-onset neurotoxicity of the parent ASOs without major reduction in knockdown activity.

These findings suggest that site-specific incorporation of 5′-CP with PO replacement can mitigate late-onset neurotoxicity of ASOs *in vivo* without compromising knockdown activity. Notably, for ASO1 and ASO4, 5′-CP at position 3 reduced knockdown activity of the parent ASOs (Figures 4E and S24B), whereas the addition of PS-3(−) maintained knockdown activity more effectively (Figures 6E and S24B), suggesting this combination is a more optimal modification for knockdown activity.

Elucidating molecular mechanism of late-onset neurotoxicity caused by PS-ASO

We hypothesized that paraspeckle protein mislocalization into nucleoli is a major mechanism underlying cytotoxicity in neural cells and the *in vivo* late-onset neurotoxicity of PS-ASO (Figure S25).[29,31,37–39,49] To investigate this hypothesis, we conducted immunohistochemistry assays on human neuronal cells transfected with the ASOs, focusing on paraspeckle proteins. Neurotoxic ASOs induced p54nrb mislocalization into the nucleoli (Figure 7A). For ASO1, 2′-OMe at position 2 and 5′-CP at positions 1, 3, and 9 reduced this mislocalization compared with the parent ASO1 (Figures 7B and 7C). These results align with the mitigation effects of the chemical modifications on cytotoxicity observed *in vitro* and the late-onset neurotoxicity *in vivo*. For ASO2, the immunohistochemistry assay revealed that 2′-OMe at gap position 2 increased this mislocalization. In contrast, 5′-CP at positions 1, 3, and 9 reduced the mislocalization dose-dependently (Figures 7D and 7E), consistent with *in vivo* assessments of neurotoxicity, except for 5′-CP at position 1, which did not reduce late-onset neurotoxicity *in vivo* (Figure 6I).

Additionally, 5′-CP at position 3 with or without PO replacement reduced p54nrb mislocalization induced by each of ASO1-4 (Figure S26). Especially in ASO2, the 5′-CP at position 3 with PS-3 (−) significantly reduced p54nrb mislocalization compared with the 5′-CP at position 3 alone, which aligns with a trend toward mitigation of late-onset neurotoxicity observed *in vivo* (Figure S22A).

These results indicate that the mislocalization of paraspeckle protein is associated with neuronal cytotoxicity and may be a key mechanism underlying the late-onset neurotoxicity. Furthermore, the effects of chemical modifications in PS-ASOs on their toxicity appear closely linked to their impact on this mislocalization phenomenon.

DISCUSSION

This study characterized the late-onset neurotoxicity induced by various gapmer-type ASOs targeting the CNS with both *in vitro* and *in vivo* models. Our findings reveal the correlation between neuronal cell death with p53 activation and motor-dominant neurological dysfunction. Furthermore, through the models, we investigated the effects of site-specific chemical modifications including our original DNA analog, 5′-CP, to mitigate this toxicity without reducing knockdown activity. Our results provide key insights into the molecular mechanisms involving the mislocalization of paraspeckle protein that underlie late-onset neurotoxicity and strategies to improve the therapeutic index of CNS-targeting ASOs.

The late-onset neurobehavioral abnormalities with our multiple ASOs manifested primarily as motor dysfunction. These symptoms progressed to impaired consciousness, reduced locomotor activity, weight loss, and eventual fatality. Especially, in the i.t. injection study, rats showed notable severe paraplegia and urinary disorders, which can be explained by disturbance at the lumbar and sacral spinal cord levels where the ASOs administered intrathecally via catheter are most efficiently distributed.

Importantly, this late-onset neurotoxicity is clearly distinct from the acute neurotoxicity in several aspects: its induction by the ASOs that show no toxicity in the acute phase and its different time course. Late-onset neurotoxicity occurs several days postinjection and persists for over 1 week, whereas acute neurotoxicity appears immediately after injection and resolves within 1 day, as previously reported.[13,14,16,45] These distinctions suggest differences in molecular mechanisms and cellular localizations of toxicity-related molecules, and pathways compared with the acute neurotoxicity, which has been previously reported to be associated with glutamate receptor inhibition, including α-amino-3-hydroxy-5-methyl-4-isoxazole propionic acid (AMPA) receptor on cell membranes, leading to ionotropic disturbance by blocking calcium influx.[14,16] In contrast, the late-onset neurotoxicity observed in this study occurred still earlier than some neurotoxic effects reported in clinical trials, which can manifest several weeks after treatment.[19] This difference in timing likely reflects interspecies variations in pharmacokinetics.[50,51]

38.4 nmol of ASO1 and ASO1 with 5′-CP position 3 PS-3(−) and sacrificed 21 days postinjection. Tolerability scores (D) and target *Mapt* mRNA levels in the left hippocampus and lumbar cord (E) were measured. (F) 5′-CP positions 1,3, or 9 with or without PO replacement in ASO2. (G) Neuro-2a cells were transfected with 2 nM ASOs and BE(2)-M17 cells for 20 nM ASOs: ASO2; ASO2 2′-OMe position 2; and ASO2 5′-CP position 1, 3, or 9 with or without PO replacement (n = 4 biological replicates per group). See also Figure S21. (H–K) Seven-week-old female C57BL/6 mice (n = 4) were injected intracerebroventricularly with 19.0 nmol ASO2, with 5′-CP positions 1, 3, or 9, with or without PO replacement, and sacrificed 14 days postinjection. (I) Tolerability scores were documented. Target *Hdac2* (J) and *Cdkn1a* (K) mRNA levels in the left hippocampus were measured 10–14 days postinjection. Symbols indicate collection days: rounds (day 14), rhombus (day 10), and red triangles (day 13, postmortem). Data in (D), (E), and (I)–(K) are presented as mean ± SEM. $^{\#}p \leq 0.05$, $^{\#\#}p \leq 0.01$, $^{\#\#\#}p \leq 0.001$, and $^{\#\#\#\#}p \leq 0.0001$; data were analyzed using Student's two-tailed t tests with vehicle. ns, not significant ($p > 0.05$). $^{*}p \leq 0.05$, $^{**}p \leq 0.01$, and $^{***}p \leq 0.001$; data were analyzed using one-way ANOVA, followed by Tukey's *post hoc* tests.

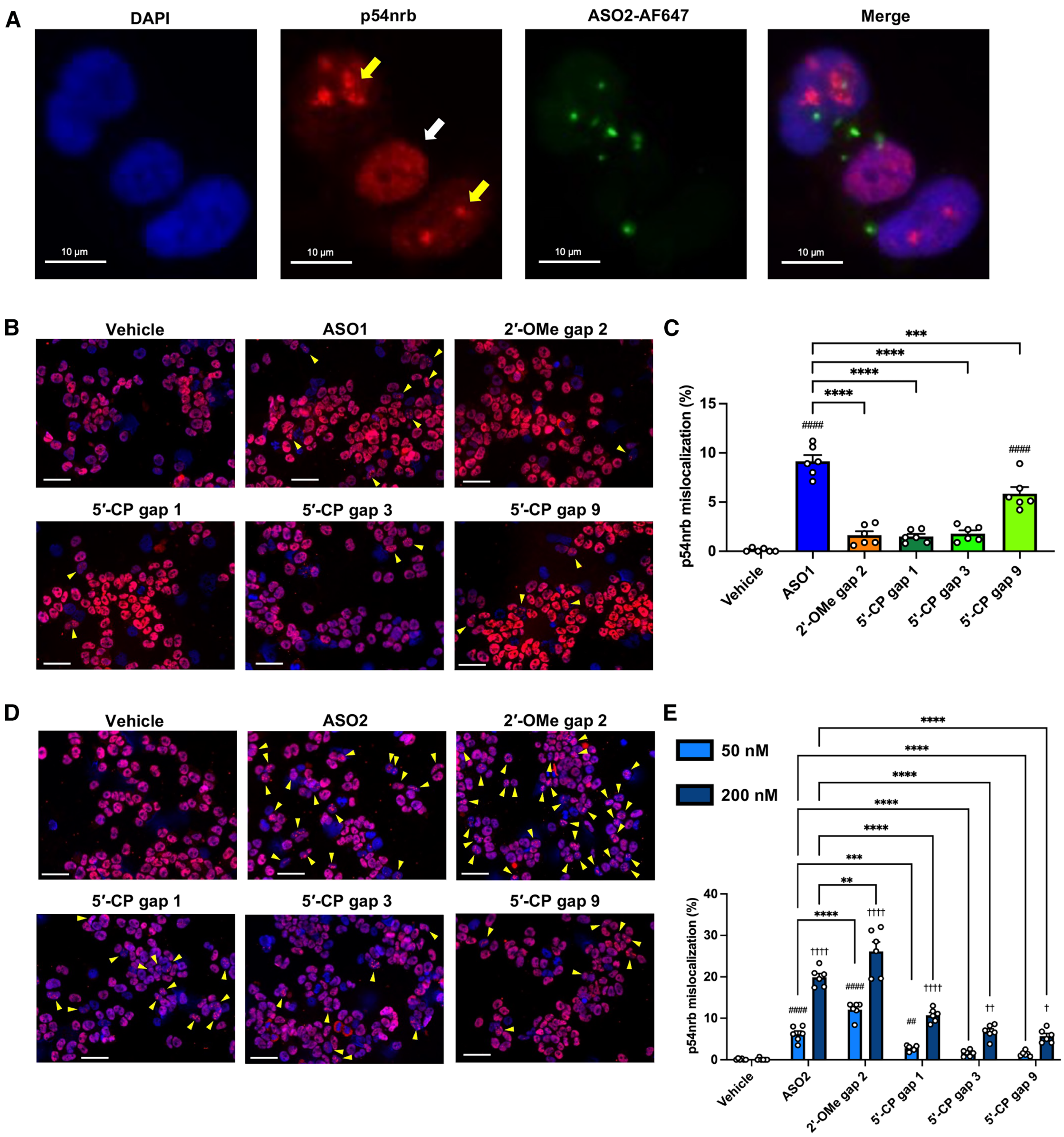

Figure 7. Mislocalization of p54nrb induced by PS-ASOs in neural cells is improved by the gap modifications

(A) Representative microscopic immunostaining images of p54nrb in BE(2)-M17 cells transfected with 200 nM of ASO2 labeled with Alexa Fluor 647 at the 3′ end for 3 h. White and yellow arrows indicate normally distributed and mislocalized p54nrb, respectively. (B and C) BE(2)-M17 cells were transfected for 3 h with 200 nM of ASO1; ASO1 modified with 2′-OMe at gap position 2; or 5′-CP at gap position 1, 3, or 9. (B) Representative microscopic immunostaining images of p54nrb. (C) Percentage of p54nrb mislocalization to nucleoli in BE(2)-M17 cells. (D and E) BE(2)-M17 cells were transfected for 3 h with 50 or 200 nM of ASO2; ASO2 modified with 2′-OMe at gap position 2; or 5′-CP at gap position 1, 3, or 9. $^{\#\#\#\#}p \leq 0.0001$; data were analyzed using Student's two-tailed t tests with vehicle. $^{***}p \leq 0.001$ and $^{****}p \leq 0.0001$; data were analyzed using one-way ANOVA, followed by Tukey's *post hoc* tests. (D) Representative microscopic immunostaining images of p54nrb. (E) Percentage of p54nrb mislocalization to nucleoli in BE(2)-M17 cells. Data in (C) and (E) are presented as mean ± SEM of different microscopic images within the same well (n = 6 per group). Scale bar, 50 μm (B and D). $^{\dagger}p \leq 0.05$, $^{\#\#, \dagger\dagger}p \leq 0.01$, and $^{\#\#\#\#, \dagger\dagger\dagger\dagger}p \leq 0.0001$; data were analyzed using Student's two-tailed t tests with vehicle ($^{\#}$ for 50 nM and † for 200 nM). $^{**}p \leq 0.01$, $^{***}p \leq 0.001$, and $^{****}p \leq 0.0001$; data were analyzed using one-way ANOVA, followed by Tukey's *post hoc* tests.

Our *in vitro* analyses provide mechanistic insights into late-onset toxicity *in vivo*, demonstrating cytotoxicity with elevated p53-regulated transcripts, *Cdkn1a* mRNA levels, and mislocalization of the paraspeckle protein, p54nrb. Both toxic phenomena were caused by the neurotoxic ASOs and improved by the site-specific gap modifications that reduced the late-onset neurotoxicity *in vivo*. Given crucial roles of paraspeckle proteins in RNA processing and nuclear organization, their mislocalization likely leads to cellular

stress and neuronal dysfunction.[33,39,49] Our histopathological analysis showed no neuronal cell death in CNS tissues of rats exhibiting lethal toxicity in neurobehavioral tests (Figures 4M and S16), suggesting that the neurological abnormalities in motor function may stem from functional impairments in neuronal cells preceding cell death.

Although 5′-CP at gap position 1 reduced p54nrb mislocalization, this modification did not reduce late-onset neurotoxicity of ASO2 *in vivo*. Other toxicity mechanisms, such as immune-stimulatory effects, may also contribute to late-onset neurotoxicity independent of toxicity from ASO-p54nrb interactions.

Our comprehensive analysis of structure-"neuro" toxicity relationship provides insights into mechanistic difference between neurotoxicity and other organ toxicity induced by ASOs. We revealed sequence-dependent effects of 2′-OMe at gap position 2 on late-onset neurotoxicity, reducing or exacerbating both the *in vitro* and *in vivo* toxicity. This modification pattern has been reported to mitigate hepatotoxicity of systemically administered PS-ASO by modulating ASO-protein interactions. However, sequence dependency in this mitigation effect of hepatotoxicity was not clear.[29] Additionally, our immunohistochemistry analysis showed that neurotoxic ASOs did not co-localize with p54nrb, whereas previous studies have shown co-localization of hepatotoxic ASOs and p54nrb.[29,38] These findings indicate that neural cytotoxicity may involve different interactions with other proteins, compared with other cytotoxicity types, indirectly leading to p54nrb mislocalization.

We demonstrated that 5′-CP gap modification has the potential to reduce late-onset neurotoxicity of PS-ASO without reducing knockdown activity *in vivo*. In particular, 5′-CP at gap position 3 significantly reduced late-onset neurotoxicity across multiple ASOs. These results are consistent with previous findings on other 5′-modifications, such as 5′-methyl or 5′-ethyl, in reducing hepatotoxicity of systemically administered PS-ASO.[30] The most favorable position for S-configured 5′-methyl DNA to reduce hepatotoxicity was position 3, consistent with our findings. From a chemical perspective, compared to the previous 5′-modifications, 5′-CP has a synthesis-related advantage of eliminating the need to control chirality, unlike 5′-methyl or 5′-ethyl. Additionally, 5′-CP in ASO2 enabled the replacement of a PS bond 5′-adjacent a DNA monomer with a PO bond without compromising knockdown activity *in vivo*, although PS-3(−) alone reduced the activity (Figures 5F and 6J). Moreover, 5′-CP at gap position 3 with PS-3(−) maintained knockdown activity more effectively than 5′-CP at gap position 3 in ASO1 and ASO4 (Figures 4E, 6E, and S24B). The greater nuclear stability of DNA with a 5′-CP compared with DNA with a PS bond may help maintain the knockdown activity of PS-ASOs *in vivo*.[40]

Several limitations warrant further investigation. First, we selected LNA for the modification in the wing region of the gapmer PS-ASO, because LNA-gapmers often show higher neurotoxic characteristics than other modifications[14] and serve as an optimal positive control for investigating neurotoxicity mitigation. Further studies are needed to optimize 5′-CP and other gap modifications for broader application across different gapmer-type ASOs, including MOE-gapmers like tofersen. Second, this study demonstrated that the mitigation of the late neurotoxicity *in vitro* and *in vivo* occurs without reducing the silencing effects on several target genes (on-target effects), indicating that change in off-target effects by our chemical modifications cannot fully explain the mitigation. However, further study examining whether off-target effects cause neurotoxicity in various ASOs will be preferable, because 5′-CP modifications decreased T_m values of the ASOs. Third, the method of *in vitro* studies was transfection with Lipofectamine 2000. Although this method does not reflect all aspects of cellular uptake with *in vivo* delivery of ASO, we believe that the findings from the experiments with this method can be useful for screening and elucidation of the neurotoxicity mechanism, because the previous reports demonstrated *in vitro* screening and mechanism analysis for ASO hepatotoxicity with this method.[29,30] Finally, our chemical modifications in the gap did not reduce the acute-onset neurotoxicity of ASO2 (data not shown), indicating the need for alternative strategies, such as G-clamp modification in the wing region of gapmer ASOs.

Our findings have important implications for developing ASO therapeutics targeting CNS diseases. The late-onset neurotoxicity observed in preclinical models, particularly with high doses, poses a significant challenge for clinical applications. Our established *in vitro* and *in vivo* models provide valuable tools for assessing the late-onset neurotoxicity of therapeutic oligonucleotides. Additionally, our findings provide mechanistic insights into the late-onset neurotoxicity of ASOs, including the mislocalization of paraspeckle proteins. Moreover, our results indicate that rational chemical modification of ASOs, particularly with 5′-CP modifications with PO replacement, offers a promising approach to reduce late-onset neurotoxicity without compromising therapeutic efficacy. This advancement potentially promotes the clinical application of ASOs for treating intractable neurological diseases, thereby expanding the therapeutic potential of this ASO strategy.

MATERIALS AND METHODS

ASO design and synthesis

DNA/LNA gapmer PS-ASOs targeting *Microtubule-associated protein tau* (*Mapt*), *Histone deacetylase 2* (*Hdac2*), *Synuclein alpha* (*SNCA*), and *Huntingtin* (*HTT*) mRNAs, as well as gap-modified PS-ASOs with 2′-OMe and 5′-CP, were synthesized by GeneDesign (Osaka, Japan) (Table S1).

In vitro toxicity evaluation: LDH assay and cell counting

Neuro-2a or BE(2)-M17 cells (1.0–3.0 × 10^4 cells) in a 48-well plate were transfected with ASOs using Lipofectamine 2000 (Thermo Fisher Scientific, Waltham, MA). Each ASO was tested in a cell line where the corresponding ASO demonstrated knockdown activity. For ASO2, BE(2)-M17 was used in cytotoxicity assays to avoid on-target effects from target *Hdac2* knockdown, which has been reported to induce apoptosis.[52–54] Lipofectamine 2000 was diluted

1:600, and incubation time was 72 h. Lipofectamine 2000 with PBS was used as the vehicle for the negative control. After transfection, LDH activity in the supernatant medium was measured with cytotoxicity LDH assay kit-water-soluble tetrazolium salt (WST) (Dojindo, Kumamoto, Japan). The absorbance was recorded with a Tecan M1000Pro (Tecan, Mänedorf, Switzerland). After removing the medium, cells were detached with trypsin, suspended in PBS, and counted with a Scepter 3.0 handheld automated cell counter (Merck, Darmstadt, Germany) or FACSCOPE B (WAKENBTECH, Kyoto, Japan). Toxicity was visualized with a color scale, with darker orange indicating higher toxicity. Experiments in Figures 3A–3D, 6G, and S21 share the same vehicle, ASO2, and ASO2 with 2′-OMe at gap position 2.

T_m measurement (RNA-binding affinity assessment)

ASOs were annealed with complementary RNA by heating at 95°C for 5 min and gradually cooled to room temperature. Melting curves were generated using TMSPC-8 temperature-controlled accessory (SHIMADZU, Kyoto, Japan) by heating from 20°C to 95°C at 260 nm. T_m values were determined from the first derivative of the absorbance curve.

Animal experiment regulations

Mice and rats were housed in a pathogen-free experimental room with controlled temperature (22°C ± 3°C) and humidity (50% ± 20%) and a 12-h light/dark cycle. All experiments complied with ethical and safety guidelines for animal research (Basic Policies for the Conduct of Animal Experiments in Research Institutions by Ministry of Education, Culture, Sports, Science and Technology and Animal Research: Reporting of In Vivo Experiments (ARRIVE) guidelines). The protocols were approved by the ethics committee of the Institute of Science Tokyo (A2025-125A). Efforts were made to minimize animal numbers, pain, and discomfort.

Intracerebroventricular administration of ASO in mice

Wild-type 7-week-old female ICR or C57BL/6 mice (Oriental Yeast or Sankyo Labo Service Corporation, Tokyo, Japan) were used depending on stock availability. The mice were randomly grouped and anesthetized with isoflurane. After fixation using SR-5M-HT (Narishige, Tokyo, Japan), a burr hole was made 1 mm to the left and 0.2 mm behind the bregma using a 0.3 mm microdrill. A needle of 1702RN Neuros syringe (Hamilton, Reno, NV) was vertically inserted into the burr hole 3 mm in depth to reach the left ventricle in 10 s using an MDS-1 micromanipulator (Narishige). Prior to the administration, ASO compounds were dissolved in vehicle at concentrations ranging from 730 to 3,100 μM. After 2 min, 13–15 μL of ASO was injected automatically over 3 min using an IMS-20 microinjector (Narishige), followed by a 5-min rest. Subsequently, the needle was pulled out in 10 s, and the scalp was closed with nylon sutures. 5 mg kg^{-1} of carprofen (0.5 mg mL^{-1}) and 0.02 μg kg^{-1} of gentamycin were subcutaneously injected postoperatively. For postmortem analyses, mice were anesthetized with isoflurane and euthanized by transcardiac perfusion with 1× PBS. The data shown in Figures 4C–4E, S4, and S13 are from the same experiment.

Intrathecal administration of ASO in rats

Wild-type 9-week-old male Slc:SD rats with spinal canal catheters (Sankyo Labo Service Corporation) were randomly grouped and single-housed. The tip of the catheter was placed at the lumbar spinal cord level. Prior to the administration, ASO compounds were dissolved in vehicle at 4,800 μM. After anesthesia with isoflurane, 40 μL of ASO was injected manually over 30 s, followed by 180 μL of artificial cerebrospinal fluid over 2 min 15 s. PBS was used as the vehicle for the negative control. For postmortem analyses, rats were anesthetized with isoflurane, the abdominal cavity was opened, and bladder volume was measured. Rats were then euthanized by transcardiac perfusion with PBS.

In vivo neurotoxicity evaluation

Behavioral assessments, locomotor activity (open-field test), and body weight were measured after ASO administration. A blinded rater scored behavior using a tolerability scoring system (Table S2). Mice were sacrificed if they scored ≥11 and could not drink water independently. Locomotor activity was tracked for 5 min using the ANY-maze video tracking system (Stoelting, Wood Stale, IL).

RNA isolation and quantitative real-time PCR assay (knockdown efficacy and toxicity-biomarker evaluation)

RNA was extracted from cells or tissues with ISOGEN, ISOGEN-LS (Nippon Gene, Tokyo, Japan), or miRNeasy mini kit (QIAGEN, Hilden, Germany). Complementary DNA was synthesized with Takara 5× PrimeScript RT master mix (Takara Bio, Shiga, Japan) and quantified by quantitative real-time PCR (real-time qPCR) with LightCycler 480 system (Roche Diagnostics, Rotkreuz, Switzerland) with LightCycler 480 probes master kit (Roche Diagnostics GmbH, Mannheim, Germany) and primers. Data were normalized with internal control mRNA (*Glyceraldehyde-3-phosphate dehydrogenase*, *Gapdh*; *Beta-actin*, *Actb*; or *Hypoxanthine phosphoribosyl transferase 1*, *Hprt*1). The primers and probes for mouse *Mapt* (Mm0052 1988_m1), *Cdkn1a* (Mm01303209_m1), *Il-6* (Mm00446190_m1), *Hdac2* (Mm00515108_m1), *Casp3* (Mm01195085_m1), *Casp7* (Mm00432322_m1), human *SNCA* (Hs01103383_m1), *CDKN1A* (Hs00355782_m1), rat *Hprt1* (Rn01527840_m1), and *Cdkn1a* (Rn00589996_m1) were purchased from Thermo Fisher Scientific. The other primers and probes used were in Table S3. All studies were performed according to Minimum Information for Publication of Quantitative Real-Time PCR Experiments (MIQE) guidelines.[55] Toxicity-biomarker levels and knockdown activity were color-coded, darker orange and darker green indicating higher toxicity and lower activity, respectively.

Immunofluorescence study

BE(2)-M17 cells were transfected with an ASO in an eight-well slide and chamber (Watson, Tokyo, Japan) for 3 h. PBS was used as the vehicle for the negative control. After removing the medium, the cells were fixed with 4% paraformaldehyde for 30 min at room temperature. After washing with ice-cold PBS three times, the cells were permeabilized with 0.1% Triton X-100 in 1× PBS for 5 min at room

temperature. The cells were washed with PBS three times and blocked with 1 mg mL^{-1} of BSA in 1× PBS overnight at 4°C. The cells were incubated in the same blocking buffer with primary antibodies against p54nrb (mouse monoclonal, sc-376865, 1:50, Santa Cruz, Dallas, TX) for 2 h at room temperature. After washing the cells with 0.1% NP-40 in 1× PBS for 5 min each, three times, the cells were incubated in the blocking buffer with 2 μg mL^{-1} of secondary antibodies against mice with Alexa Fluor 568 (goat immunoglobulin G, A11004, 1:1000, Thermo Fisher Scientific) for 1 h at room temperature. After washing with PBS three times, the cells were mounted with an antifade mounting medium with 4′,6-diamidino-2-phenylindole (VECTASHIELD, Vector Laboratories, Newark, CA). Immunofluorescence images were acquired with a confocal microscope BZ-X700 (KEYENCE, Osaka, Japan), and p54nrb mislocalization was manually counted.

Histological study of rat brain and spinal cord

After euthanasia, tissues were immediately fixed with 4% paraformaldehyde, dehydrated with step-by-step increasing concentrations of ethanol, cleared with xylene to remove ethanol, and infiltrated with paraffin at 65°C. Using a HistoStar paraffin embedding station (Thermo Fisher Scientific), the samples were embedded in paraffin and, after cooling, sectioned and mounted on slide glasses using a microtome REM-710 (Yamato Kohki, Saitama, Japan). After deparaffinization and rehydration, samples were stained with hematoxylin and eosin. Microscopic images were acquired using a confocal microscope BZ-X700 (KEYENCE).

ASO delivery evaluation in mouse brain

Mice were intracerebroventricularly injected with Alexa Fluor 647-labeled ASO as described earlier. Brain samples were obtained and homogenized upon weight measurement at 24 h or 11 days post-injection. The fluorescence intensity of the homogenate was measured with a Tecan M1000Pro (Tecan). The amount of ASO delivered to the brain tissue was calculated based on the weight and corrections from the calibration curve.

Statistical analysis

Data are presented as mean with or without standard error of the mean (SEM). Statistical significance was determined with GraphPad Prism 10 software (version 10.1.0). Multiple comparisons among three or more groups were analyzed using one-way analysis of variance (ANOVA), followed by *post hoc* two-sided Dunnett's tests for Figures 1B and 1C, S2, and S3 or Tukey's tests for the rest. Differences between two groups were analyzed using Student's two-tailed t tests. Significance was defined as $^{*}p \leq 0.05$, $^{**}p \leq 0.01$, $^{***}p \leq 0.001$, and $^{****}p \leq 0.0001$. # or † indicates significance against vehicle: $^{\#,\ \dagger}p \leq 0.05$, $^{\#\#,\ \dagger\dagger}p \leq 0.01$, $^{\#\#\#}p \leq 0.001$, and $^{\#\#\#\#,\ \dagger\dagger\dagger\dagger}p \leq 0.0001$. The p values of >0.05 but ≤0.1 are partially indicated.

DATA AVAILABILITY

All data presented in the main text and the supplemental information are available from the corresponding authors upon request.

ACKNOWLEDGMENTS

This study was supported by Japan Agency for Medical Research and Development (JP21wm0525032 to K.Y.; JP18am0301003, JP20am0401006, and JP21ae0121026 to T. Yokota.; and JP19am0401003 to S.O. and T. Yamaguchi.); Japan Science and Technology Agency FOREST Program (JPMJFR216H to K.Y.); Japan Society for the Promotion of Science KAKENHI Grant-in-Aid for Challenging Research (Exploratory) (JP20K21882 to K.Y.) and Scientific Research (B) from MEXT (JP22H02979 to K.Y.); and Takeda Science Foundation (to K.Y.). We thank S. Ebihara and T. Ishii for supervising the histopathological study. The authors would like to thank Enago (www.enago.jp) for the English language review.

AUTHOR CONTRIBUTIONS

T.K.: conceptualization, data curation, formal analysis, investigation, methodology, project administration, visualization, and writing the original draft. K.Y.: conceptualization, formal analysis, funding acquisition, investigation, methodology, project administration, supervision, visualization, and writing the original draft. S.S.L.M.: conceptualization, data curation, formal analysis, investigation, methodology, and resources. M.K.: investigation. K.S.: investigation and resources. E.I.: investigation and methodology. K.Y.-T.: investigation and resources. R.I.-H.: conceptualization, project administration, and supervision. T. Yamaguchi: conceptualization, funding acquisition, project administration, and supervision. S.O.: conceptualization, funding acquisition, project administration, and supervision. T. Yokota: conceptualization, funding acquisition, project administration, and supervision.

DECLARATION OF INTERESTS

T. Yokota collaborates with Takeda Pharmaceuticals Co., Ltd.; Daiichi Sankyo Co., Ltd.; Rena Therapeutics Co., Ltd.; Toray Industries Co., Ltd.; Eisai Co., Ltd.; and Sumitomo Pharma Co., Ltd., and serves as the academic adviser for Rena Therapeutics, Inc., and Braizon Therapeutics, Inc. S.O. collaborates with Luxna Biotech Co., Ltd. A patent (WO 2024/071099 A1) describing the work and chemistry used in this study has been submitted (authors: T. Yokota, K.Y., T.K., T. Yamaguchi, and S.O.).

SUPPLEMENTAL INFORMATION

Supplemental information can be found online at https://doi.org/10.1016/j.omtn.2025.102692.

REFERENCES

1. Bennett, C.F., Krainer, A.R., and Cleveland, D.W. (2019). Antisense Oligonucleotide Therapies for Neurodegenerative Diseases. Annu. Rev. Neurosci. *42*, 385–406.
2. Egli, M., and Manoharan, M. (2023). Chemistry, structure and function of approved oligonucleotide therapeutics. Nucleic Acids Res. *51*, 2529–2573.
3. Collotta, D., Bertocchi, I., Chiapello, E., and Collino, M. (2023). Antisense oligonucleotides: a novel Frontier in pharmacological strategy. Front. Pharmacol. *14*, 1304342.
4. Roberts, T.C., Langer, R., and Wood, M.J.A. (2020). Advances in oligonucleotide drug delivery. Nat. Rev. Drug Discov. *19*, 673–694.
5. McDowall, S., Aung-Htut, M., Wilton, S., and Li, D. (2024). Antisense oligonucleotides and their applications in rare neurological diseases. Front. Neurosci. *18*, 1414658.
6. Miller, T.M., Cudkowicz, M.E., Genge, A., Shaw, P.J., Sobue, G., Bucelli, R.C., Chiò, A., Van Damme, P., Ludolph, A.C., Glass, J.D., et al. (2022). Trial of Antisense Oligonucleotide Tofersen for SOD1 ALS. N. Engl. J. Med. *387*, 1099–1110.
7. Finkel, R.S., Mercuri, E., Darras, B.T., Connolly, A.M., Kuntz, N.L., Kirschner, J., Chiriboga, C.A., Saito, K., Servais, L., Tizzano, E., et al. (2017). Nusinersen versus Sham Control in Infantile-Onset Spinal Muscular Atrophy. N. Engl. J. Med. *377*, 1723–1732.
8. Kim, J., Hu, C., Moufawad El Achkar, C., Black, L.E., Douville, J., Larson, A., Pendergast, M.K., Goldkind, S.F., Lee, E.A., Kuniholm, A., et al. (2019). Patient-Customized Oligonucleotide Therapy for a Rare Genetic Disease. N. Engl. J. Med. *381*, 1644–1652.
9. Kim, J., Woo, S., de Gusmao, C.M., Zhao, B., Chin, D.H., DiDonato, R.L., Nguyen, M.A., Nakayama, T., Hu, C.A., Soucy, A., et al. (2023). A framework for individualized splice-switching oligonucleotide therapy. Nature *619*, 828–836.

10. Wilton-Clark, H., Yan, E., and Yokota, T. (2024). Preparing for Patient-Customized N-of-1 Antisense Oligonucleotide Therapy to Treat Rare Diseases. Genes *15*, 821.

11. Van Daele, S.H., Masrori, P., Van Damme, P., and Van Den Bosch, L. (2024). The sense of antisense therapies in ALS. Trends Mol. Med. *30*, 252–262.

12. Moazami, M.P., Rembetsy-Brown, J.M., Sarli, S.L., McEachern, H.R., Wang, F., Ohara, M., Wagh, A., Kelly, K., Krishnamurthy, P.M., Weiss, A., et al. (2024). Quantifying and mitigating motor phenotypes induced by antisense oligonucleotides in the central nervous system. Mol. Ther. *32*, 4401–4417.

13. Miller, R., Paquette, J., Barker, A., Sapp, E., McHugh, N., Bramato, B., Yamada, N., Alterman, J., Echeveria, D., Yamada, K., et al. (2024). Preventing acute neurotoxicity of CNS therapeutic oligonucleotides with the addition of Ca^{2+} and Mg^{2+} in the formulation. Mol. Ther. Nucleic Acids *35*, 102359.

14. Jia, C., Lei Mon, S.S., Yang, Y., Katsuyama, M., Yoshida-Tanaka, K., Nagata, T., Yoshioka, K., and Yokota, T. (2023). Change of intracellular calcium level causes acute neurotoxicity by antisense oligonucleotides via CSF route. Mol. Ther. Nucleic Acids *31*, 182–196.

15. Easton, A., Jensen, M.L., Wang, C., Hagedorn, P.H., Li, Y., Weed, M., Meredith, J.E., Guss, V., Jones, K., Gill, M., et al. (2022). Identification and characterization of a MAPT-targeting locked nucleic acid antisense oligonucleotide therapeutic for tauopathies. Mol. Ther. Nucleic Acids *29*, 625–642.

16. Hagedorn, P.H., Brown, J.M., Easton, A., Pierdomenico, M., Jones, K., Olson, R.E., Mercer, S.E., Li, D., Loy, J., Høg, A.M., et al. (2022). Acute Neurotoxicity of Antisense Oligonucleotides After Intracerebroventricular Injection Into Mouse Brain Can Be Predicted from Sequence Features. Nucleic Acid Ther. *32*, 151–162.

17. Simonini, C., Zucchi, E., Martinelli, I., Gianferrari, G., Lunetta, C., Sorarù, G., Trojsi, F., Pepe, R., Piras, R., Giacchino, M., et al. (2025). Neurodegenerative and neuroinflammatory changes in SOD1-ALS patients receiving tofersen. Sci. Rep. *15*, 11034.

18. Lovett, A., Chary, S., Babu, S., Bruneteau, G., Glass, J.D., Karlsborg, M., Ladha, S., Mayl, K., McDermott, C., Bucelli, R.C., et al. (2025). Serious Neurologic Adverse Events in Tofersen Clinical Trials for Amyotrophic Lateral Sclerosis. Muscle Nerve *71*, 1006–1015.

19. Wiesenfarth, M., Dorst, J., Brenner, D., Elmas, Z., Parlak, Ö., Uzelac, Z., Kandler, K., Mayer, K., Weiland, U., Herrmann, C., et al. (2024). Effects of tofersen treatment in patients with SOD1-ALS in a "real-world" setting - a 12-month multicenter cohort study from the German early access program. eClinicalMedicine *69*, 102495.

20. van Roon-Mom, W., Ferguson, C., and Aartsma-Rus, A. (2023). From Failure to Meet the Clinical Endpoint to U.S. Food and Drug Administration Approval: 15th Antisense Oligonucleotide Therapy Approved Qalsody (Tofersen) for Treatment of SOD1 Mutated Amyotrophic Lateral Sclerosis. Nucleic Acid Ther. *33*, 234–237.

21. McCampbell, A., Cole, T., Wegener, A.J., Tomassy, G.S., Setnicka, A., Farley, B.J., Schoch, K.M., Hoye, M.L., Shabsovich, M., Sun, L., et al. (2018). Antisense oligonucleotides extend survival and reverse decrement in muscle response in ALS models. J. Clin. Investig. *128*, 3558–3567.

22. McColgan, P., Thobhani, A., Boak, L., Schobel, S.A., Nicotra, A., Palermo, G., Trundell, D., Zhou, J., Schlegel, V., Sanwald Ducray, P., et al. (2023). Tominersen in Adults with Manifest Huntington's Disease. N. Engl. J. Med. *389*, 2203–2205.

23. Estevez-Fraga, C., Tabrizi, S.J., and Wild, E.J. (2023). Huntington's Disease Clinical Trials Corner: August 2023. J. Huntingtons Dis. *12*, 169–185.

24. Aronin, N. (2022). Perspective: Tominersen Testing Finds a Way Forward. J. Huntingtons Dis. *11*, 119–120.

25. Korobeynikov, V.A., Lyashchenko, A.K., Blanco-Redondo, B., Jafar-Nejad, P., and Shneider, N.A. (2022). Antisense oligonucleotide silencing of FUS expression as a therapeutic approach in amyotrophic lateral sclerosis. Nat. Med. *28*, 104–116.

26. Hagemann, T.L., Powers, B., Lin, N.H., Mohamed, A.F., Dague, K.L., Hannah, S.C., Bachmann, G., Mazur, C., Rigo, F., Olsen, A.L., et al. (2021). Antisense therapy in a rat model of Alexander disease reverses GFAP pathology, white matter deficits, and motor impairment. Sci. Transl. Med. *13*, eabg4711.

27. Mummery, C.J., Börjesson-Hanson, A., Blackburn, D.J., Vijverberg, E.G.B., De Deyn, P.P., Ducharme, S., Jonsson, M., Schneider, A., Rinne, J.O., Ludolph, A.C., et al. (2023). Tau-targeting antisense oligonucleotide MAPT(Rx) in mild Alzheimer's disease: a phase 1b, randomized, placebo-controlled trial. Nat. Med. *29*, 1437–1447.

28. Eckstein, F. (2014). Phosphorothioates, essential components of therapeutic oligonucleotides. Nucleic Acid Ther. *24*, 374–387.

29. Shen, W., De Hoyos, C.L., Migawa, M.T., Vickers, T.A., Sun, H., Low, A., Bell, T.A., Rahdar, M., Mukhopadhyay, S., Hart, C.E., et al. (2019). Chemical modification of PS-ASO therapeutics reduces cellular protein-binding and improves the therapeutic index. Nat. Biotechnol. *37*, 640–650.

30. Vasquez, G., Freestone, G.C., Wan, W.B., Low, A., De Hoyos, C.L., Yu, J., Prakash, T. P., Ǿstergaard, M.E., Liang, X.H., Crooke, S.T., et al. (2021). Site-specific incorporation of 5'-methyl DNA enhances the therapeutic profile of gapmer ASOs. Nucleic Acids Res. *49*, 1828–1839.

31. Crooke, S.T., Vickers, T.A., and Liang, X.H. (2020). Phosphorothioate modified oligonucleotide-protein interactions. Nucleic Acids Res. *48*, 5235–5253.

32. Anderson, B.A., Freestone, G.C., Low, A., De-Hoyos, C.L., Iii, W.J.D., Østergaard, M.E., Migawa, M.T., Fazio, M., Wan, W.B., Berdeja, A., et al. (2021). Towards next generation antisense oligonucleotides: mesylphosphoramidate modification improves therapeutic index and duration of effect of gapmer antisense oligonucleotides. Nucleic Acids Res. *49*, 9026–9041.

33. Shen, W., De Hoyos, C.L., Sun, H., Vickers, T.A., Liang, X.H., and Crooke, S.T. (2018). Acute hepatotoxicity of 2' fluoro-modified 5-10-5 gapmer phosphorothioate oligonucleotides in mice correlates with intracellular protein binding and the loss of DBHS proteins. Nucleic Acids Res. *46*, 2204–2217.

34. Liang, X.H., Sun, H., Shen, W., and Crooke, S.T. (2015). Identification and characterization of intracellular proteins that bind oligonucleotides with phosphorothioate linkages. Nucleic Acids Res. *43*, 2927–2945.

35. Bailey, J.K., Shen, W., Liang, X.H., and Crooke, S.T. (2017). Nucleic acid binding proteins affect the subcellular distribution of phosphorothioate antisense oligonucleotides. Nucleic Acids Res. *45*, 10649–10671.

36. Kakiuchi-Kiyota, S., Whiteley, L.O., Ryan, A.M., and Mathialagan, N. (2016). Development of a Method for Profiling Protein Interactions with LNA-Modified Antisense Oligonucleotides Using Protein Microarrays. Nucleic Acid Ther. *26*, 93–101.

37. Liang, X.H., De Hoyos, C.L., Shen, W., Zhang, L., Fazio, M., and Crooke, S.T. (2021). Solid-Phase Separation of Toxic Phosphorothioate Antisense Oligonucleotide-Protein Nucleolar Aggregates Is Cytoprotective. Nucleic Acid Ther. *31*, 126–144.

38. Shen, W., Liang, X.H., and Crooke, S.T. (2014). Phosphorothioate oligonucleotides can displace NEAT1 RNA and form nuclear paraspeckle-like structures. Nucleic Acids Res. *42*, 8648–8662.

39. Vickers, T.A., Rahdar, M., Prakash, T.P., and Crooke, S.T. (2019). Kinetic and subcellular analysis of PS-ASO/protein interactions with P54nrb and RNase H1. Nucleic Acids Res. *47*, 10865–10880.

40. Obika, S., Yamaguchi, T., Sakaue, S., Inoue, T., Yoshida, T. (2023). Patent No. WO2023167094A1.

41. Hagedorn, P., Olson, R.E., Høg, A.M., Jensen, M.L., Li, D., Nielsen, N.F. (2018). Patent No. US20180023081A1.

42. Seth, P.P., Oestergaard, M., Migawa, M.T., Liang, X.H., Shen, W., Crooke, S.T., Swayze, E.E. (2021). Patent No. US20210261945A1.

43. Hagedorn, P., Olson, R.E., Cacace, A.M., Jensen, M.L., Brown, J.M., Meredith, J.E., Pendri, A., Mcdonald, I.M., Gill, M. (2019). Patent No. WO2019138057A1.

44. Rook, M.E., and Southwell, A.L. (2022). Antisense Oligonucleotide Therapy: From Design to the Huntington Disease Clinic. BioDrugs *36*, 105–119.

45. Matsubayashi, T., Yoshioka, K., Lei Mon, S.S., Katsuyama, M., Jia, C., Yamaguchi, T., Hara, R.I., Nagata, T., Nakagawa, O., Obika, S., and Yokota, T. (2024). Favorable efficacy and reduced acute neurotoxicity by antisense oligonucleotides with 2',4'-BNA/LNA with 9-(aminoethoxy)phenoxazine. Mol. Ther. Nucleic Acids *35*, 102161.

46. Freier, S.M., and Altmann, K.H. (1997). The ups and downs of nucleic acid duplex stability: structure-stability studies on chemically-modified DNA:RNA duplexes. Nucleic Acids Res. *25*, 4429–4443.

47. Tran, H., Moazami, M.P., Yang, H., McKenna-Yasek, D., Douthwright, C.L., Pinto, C., Metterville, J., Shin, M., Sanil, N., Dooley, C., et al. (2022). Suppression of mutant C9orf72 expression by a potent mixed backbone antisense oligonucleotide. Nat. Med. *28*, 117–124.

48. Le, B.T., Chen, S., and Veedu, R.N. (2024). Rational Design of Chimeric Antisense Oligonucleotides on a Mixed PO-PS Backbone for Splice-Switching Applications. Biomolecules *14*, 883.

49. Mircsof, D., Langouët, M., Rio, M., Moutton, S., Siquier-Pernet, K., Bole-Feysot, C., Cagnard, N., Nitschke, P., Gaspar, L., Žnidarič, M., et al. (2015). Mutations in NONO lead to syndromic intellectual disability and inhibitory synaptic defects. Nat. Neurosci. *18*, 1731–1736.

50. Mortberg, M.A., Gentile, J.E., Nadaf, N.M., Vanderburg, C., Simmons, S., Dubinsky, D., Slamin, A., Maldonado, S., Petersen, C.L., Jones, N., et al. (2023). A single-cell map of antisense oligonucleotide activity in the brain. Nucleic Acids Res. *51*, 7109–7124.

51. Jafar-Nejad, P., Powers, B., Soriano, A., Zhao, H., Norris, D.A., Matson, J., DeBrosse-Serra, B., Watson, J., Narayanan, P., Chun, S.J., et al. (2021). The atlas of RNase H antisense oligonucleotide distribution and activity in the CNS of rodents and non-human primates following central administration. Nucleic Acids Res. *49*, 657–673.

52. Schüler, S., Fritsche, P., Diersch, S., Arlt, A., Schmid, R.M., Saur, D., and Schneider, G. (2010). HDAC2 attenuates TRAIL-induced apoptosis of pancreatic cancer cells. Mol. Cancer *9*, 80.

53. Huang, B.H., Laban, M., Leung, C.H.W., Lee, L., Lee, C.K., Salto-Tellez, M., Raju, G. C., and Hooi, S.C. (2005). Inhibition of histone deacetylase 2 increases apoptosis and p21Cip1/WAF1 expression, independent of histone deacetylase 1. Cell Death Differ. *12*, 395–404.

54. Wang, M., Liao, J., Wang, J., Xu, M., Cheng, Y., Wei, L., and Huang, A. (2024). HDAC2 promotes autophagy-associated HCC malignant progression by transcriptionally activating LAPTM4B. Cell Death Dis. *15*, 593.

55. Bustin, S.A., Benes, V., Garson, J.A., Hellemans, J., Huggett, J., Kubista, M., Mueller, R., Nolan, T., Pfaffl, M.W., Shipley, G.L., et al. (2009). The MIQE guidelines: minimum information for publication of quantitative real-time PCR experiments. Clin. Chem. *55*, 611–622.

OMTN, Volume *36*

Supplemental information

Unraveling and controlling late-onset neurotoxicity of antisense oligonucleotides through strategic chemical modifications

Takayuki Kuroda, Kotaro Yoshioka, Su Su Lei Mon, Maho Katsuyama, Kumiko Sato, Eriko Isogai, Kie Yoshida-Tanaka, Rintaro Iwata-Hara, Takao Yamaguchi, Satoshi Obika, and Takanori Yokota

Table S1. Antisense oligonucleotide sequences.

Mapt: microtubule-associated protein tau; *Hdac2*: histone deacetylase 2; *SNCA*: synuclein alpha; *HTT*: huntingtin. Bold letters indicate LNA, bold and italic letters indicate 2′-MOE, and regular letters indicate DNA. C(5) denotes 5-methylcytosine. All internucleoside linkages are phosphorothioate (PS)-bonds, except at underlined positions, which indicate substitution of the PS-bond with a phosphodiester (PO)-bond at the 5′-adjacent site of the DNA monomer.

Name	Target mRNA	Sequence
**ASO1**	*Mapt*	**5′- ATT**TCCAAATTCA**C(5)TT**
**ASO2**	*Hdac2*	**5′- TAG**TCTCTGTCAG**TTA**
**ASO3**	*SNCA*	**5′- C(5)TC(5)A**CCATTTATATAC**AAAC(5)**
**ASO4**	*SNCA*	**5′- C(5)C(5)A**TTTATATACAAA**C(5)AC(5)A**
**ASO5**	*HTT*	**5′-** ***C(5)TC(5)AG***TAAC(5)ATTGAC(5)***AC(5)C(5)AC(5)***

Table S2. Tolerability scoring system.

The tolerability scoring system used in the in mouse (**A**) and rat (**B**) experiments. N.A.: not applicable.

A

		Score 1	Score 2	Score 3	Score 4
Category 1 Consciousness	Decreased exploration or voluntary movement	Decreased exploration	Decreased exploration	No exploration	No exploration
	Decreased responsiveness	Normal	Slightly (e.g., reduced response to touch or handling)	Moderately (e.g., reduced response to lift, no blink)	Marked (e.g., reduced response to a tail pinch)
Category 2 Motor function	Ataxia	(-)	Mild (e.g., slow righting response, swaying)	Moderate (e.g., staggering, impaired walking)	Severe (e.g., crawling)
	Strength	Decreased strength of lower limbs	No ability to climb a slope	No ability to walk	No ability to right
Category 3 Appearance	Abnormal posture	Slight	Mild (e.g., hunched, extended, low posture, straub tail)	Moderate (e.g., ventral recumbency)	Severe (e.g., lateral recumbency)
	Abnormal breathing	Normal	Normal	Shallow	Labored
Category 4 Hyperactivity	Increased home-cage exploration	Slightly increased (e.g., increased exploration)	Increased (e.g., digging, burying)	Moderately increased (e.g., scratching limbs)	Marked increased
	Stereotype	(-)	Slight (e.g., increased grooming)	Moderate (e.g., circling, repetitive behavior)	Marked
Category 5 Involuntary movement	Tremors	Detectable	Marked	N.A.	N.A.
	Seizure (e.g., running, bouncing, clonic and/or tonic seizures)	(-)	(-)	Few or partial	Repeated or continuous (e.g., >1 min)

B

		Score 1	Score 2	Score 3	Score 4
Category 1 Consciousness	Decreased exploration or voluntary movement	Decreased exploration	Decreased exploration	No exploration	No exploration
	Decreased responsiveness	Normal	Slightly (e.g., reduced response to touch or handling)	Moderately (e.g., reduced response to lift, no blink)	Marked (e.g., reduced response to a tail pinch)
Category 2 Motor function	Ataxia	Slight (e.g., swaying)	Mild (e.g., crawling)	Moderate (e.g., rolling around)	Severe (e.g., no ability to move)
	Strength	Decreased strength of lower limbs	No ability to walk on four limbs	No ability to right without movement of lower limbs	No movement of four limbs
Category 3 Appearance	Abnormal posture	Slight	Mild (e.g., hunched, extended, low posture, straub tail)	Moderate (e.g., ventral recumbency with head up)	Severe (e.g., ventral recumbency without head up)
	Abnormal breathing	Normal	Normal	Shallow	Labored
Category 4 Hyperactivity	Increased home-cage exploration	Slightly increased (e.g., increased exploration)	Increased (e.g., digging, burying)	Moderately increased (e.g., scratching limbs)	Marked increased
	Stereotype	(-)	Slight (e.g., increased grooming)	Moderate (e.g., circling, repetitive behavior)	Marked
Category 5 Involuntary movement	Tremors	Detectable	Marked	N.A.	N.A.
	Seizure (e.g., running, bouncing, clonic and/or tonic seizures)	(-)	(-)	Few or partial	Repeated or continuous (e.g., >1 min)

Table S3. Primer and probe sets for qRT-PCR.

Mouse	*Gapdh*	Forward	5′ -GGTTGTCTCCTGCGACTTCAA-3′
		Reverse	5′ -CCGTATTCATTGTCATACCAGGAAA-3′
		Probe	5′ -CTCCCACTCTTCCACCTTCGATGCCG-3′
Human	*ACTB*	Forward	5′ -CGGACTATGACTTAGTTGCGTTACA-3′
		Reverse	5′ -GCCATGCCAATCTCATCTTGT-3′
		Probe	5′ -CCTTTCTTGACAAAACCTAACTTGCGCAGA-3′
	GAPDH	Forward	5′ -GAAGGTGAAGGTCGGAGTC-3′
		Reverse	5′ -GAAGATGGTGATGGGATTTC-3′
		Probe	5′ -CAAGCTTCCCGTTCTCAGCC-3′

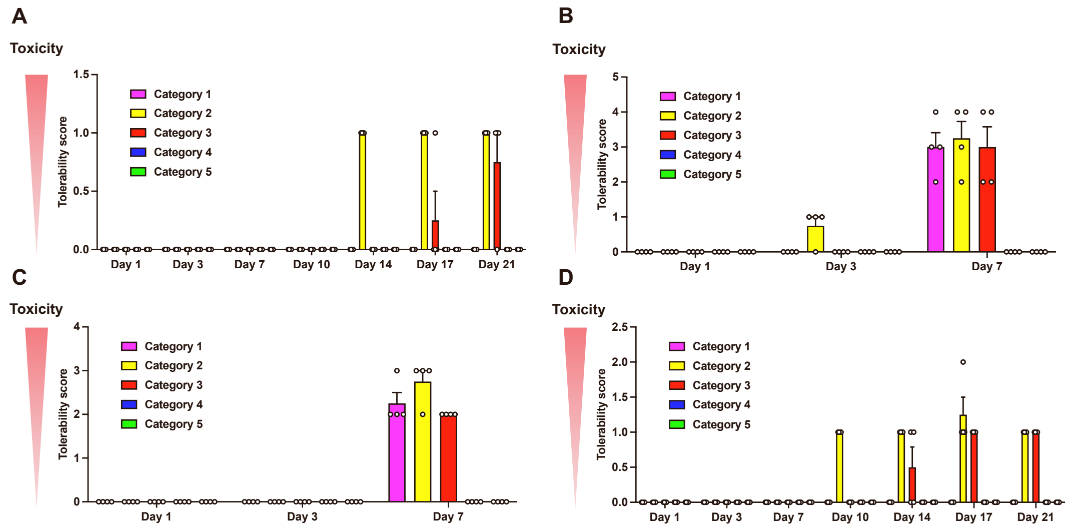

Figure S1. Breakdown of tolerability score by category in mice post-PS-ASO injection.

Seven-week-old female C57BL/6 mice (n = 4 per group) were injected intracerebroventricularly with (**A**) ASO1 (38.4 nmol, 200 µg), (**B**) ASO2 (19.0 nmol, 100 µg), (**C**) ASO3 (15.2 nmol, 100 µg), (**D**) ASO4 (39.9 nmol, 250 µg). Category1: consciousness; Category 2: motor function; Category 3: appearance; Category 4: hyperactivity; Category 5: involuntary movement. Data are presented as mean ± SEM.

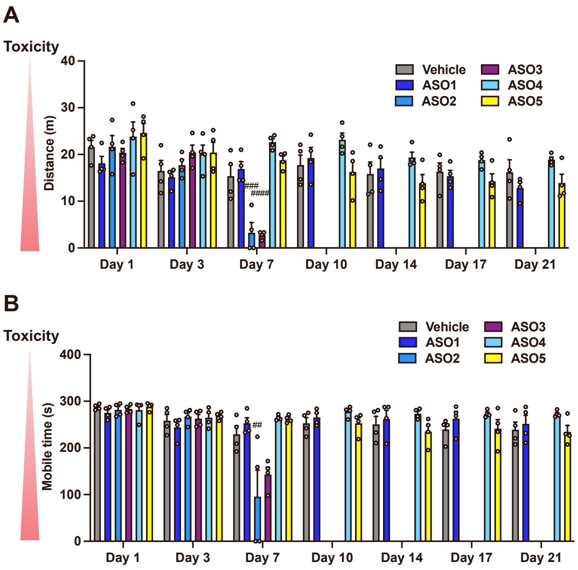

Figure S2. Distance traveled and mobile time in open-field tests in mice post-PS-ASO injection.

Seven-week-old female C57BL/6 mice (n = 4 per group) were injected intracerebroventricularly with ASO1 (38.4 nmol, 200 µg), ASO2 (19.0 nmol, 100 µg), ASO3 (15.2 nmol, 100 µg), ASO4 (39.9 nmol, 250 µg), or ASO5 (39.9 nmol, 280 µg). Mice treated with ASO2 and ASO3 were evaluated up to day 7. (**A**) Distance traveled and (**B**) mobile time in open-field tests. Data are presented as mean ± SEM. # indicates significance against vehicle.

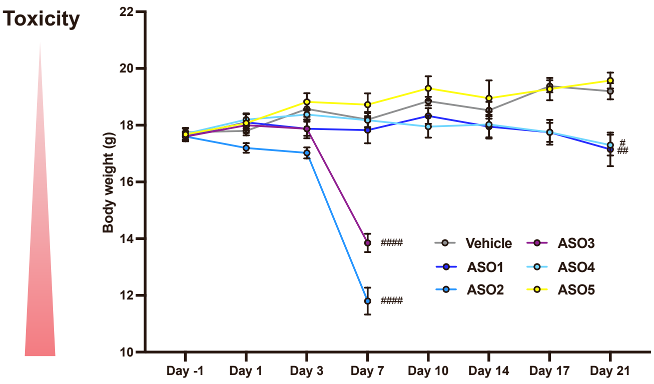

Figure S3. Changes in body weight in mice post-PS-ASO injection.

Seven-week-old female C57BL/6 mice (n = 4 per group) were injected intracerebroventricularly with ASO1 (38.4 nmol, 200 µg), ASO2 (19.0 nmol, 100 µg), ASO3 (15.2 nmol, 100 µg), ASO4 (39.9 nmol, 250 µg), or ASO5 (39.9 nmol, 280 µg). Mice treated with ASO2 and ASO3 were evaluated up to day 7. Data are presented as mean ± SEM. # indicates significance against vehicle.

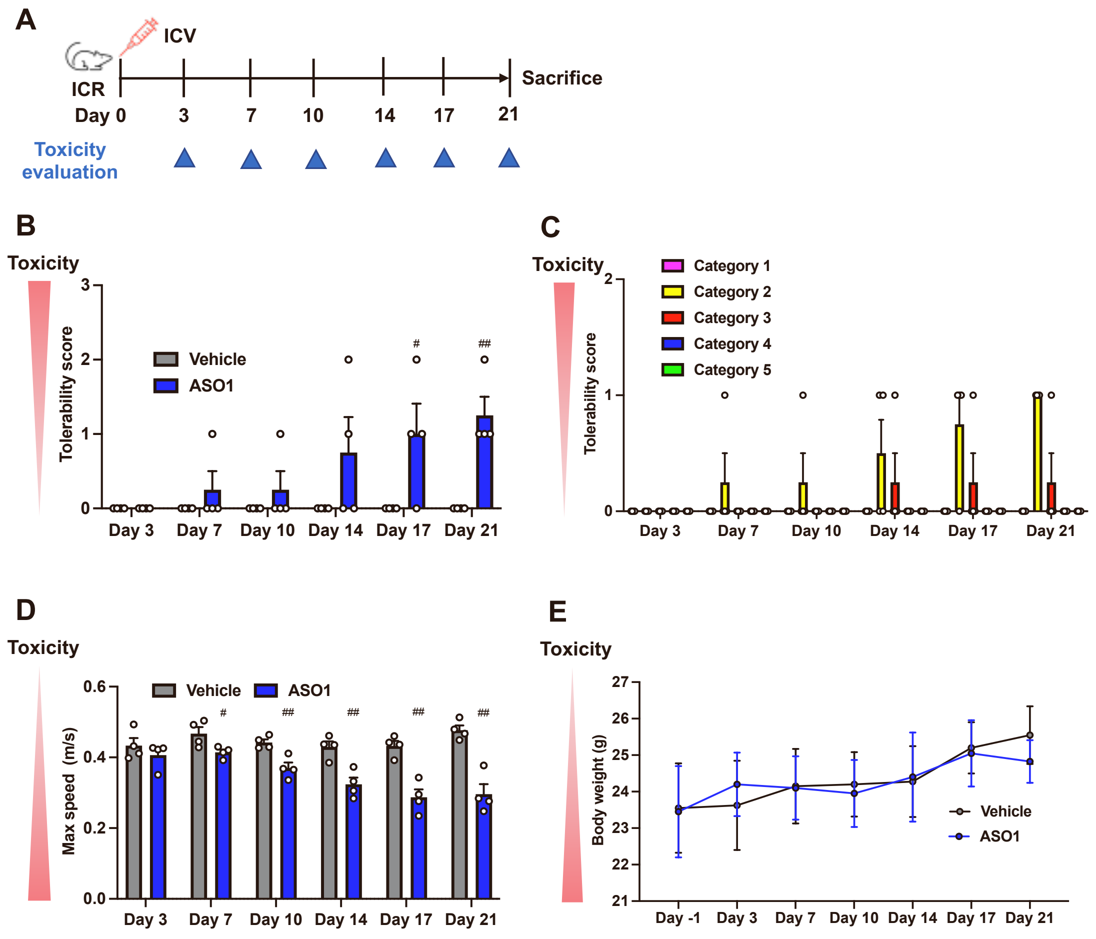

Figure S4. Late-onset neurotoxicity in mice after ICV-injection of ASO1.

(**A-E**) Seven-week-old female ICR mice (n = 4 per group) were injected intracerebroventricularly with 38.4 nmol (200 µg) of ASO1. Toxicity was assessed on the indicated days, and mice were sacrificed 21 days postinjection. (**B**) Tolerability scores over time postinjection. (**C**) Breakdown of tolerability score by category. (**D**) Maximum speed in open-field tests at the indicated time points. (**E**) Changes in body weight over time. Data are presented as mean ± SEM. # indicates significance against vehicle.

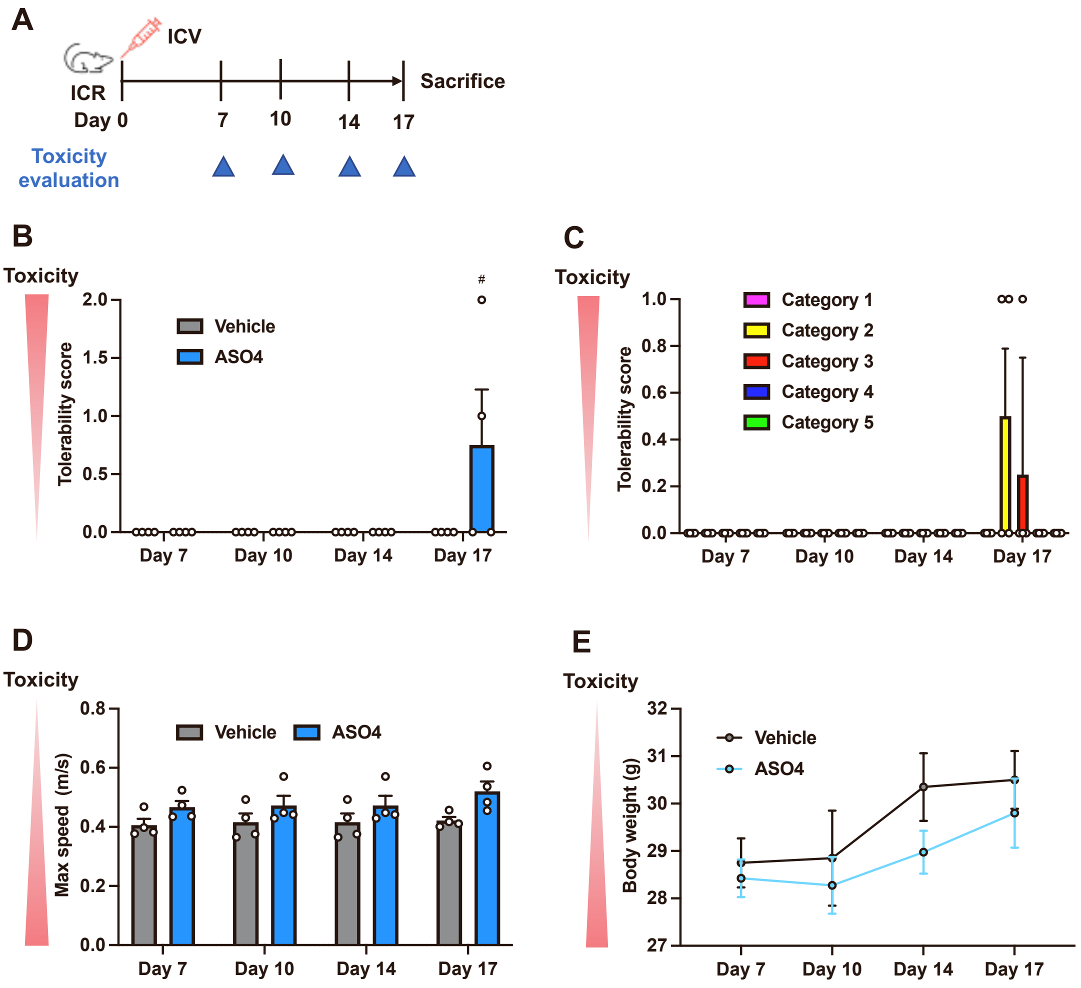

Figure S5. Late-onset neurotoxicity in mice after ICV-injection of ASO4.

(**A-E**) Seven-week-old female ICR mice (n = 4 per group) were injected intracerebroventricularly with 39.9 nmol (250 µg) ASO4. Toxicity was assessed on the indicated days, and mice were sacrificed 17 days postinjection. (**B**) Tolerability scores over time postinjection. (**C**) Breakdown of tolerability score by category. (**D**) Maximum speed in open-field tests at the indicated time points. (**E**) Changes in body weight over time. Data are presented as mean ± SEM. # indicates significance against vehicle.

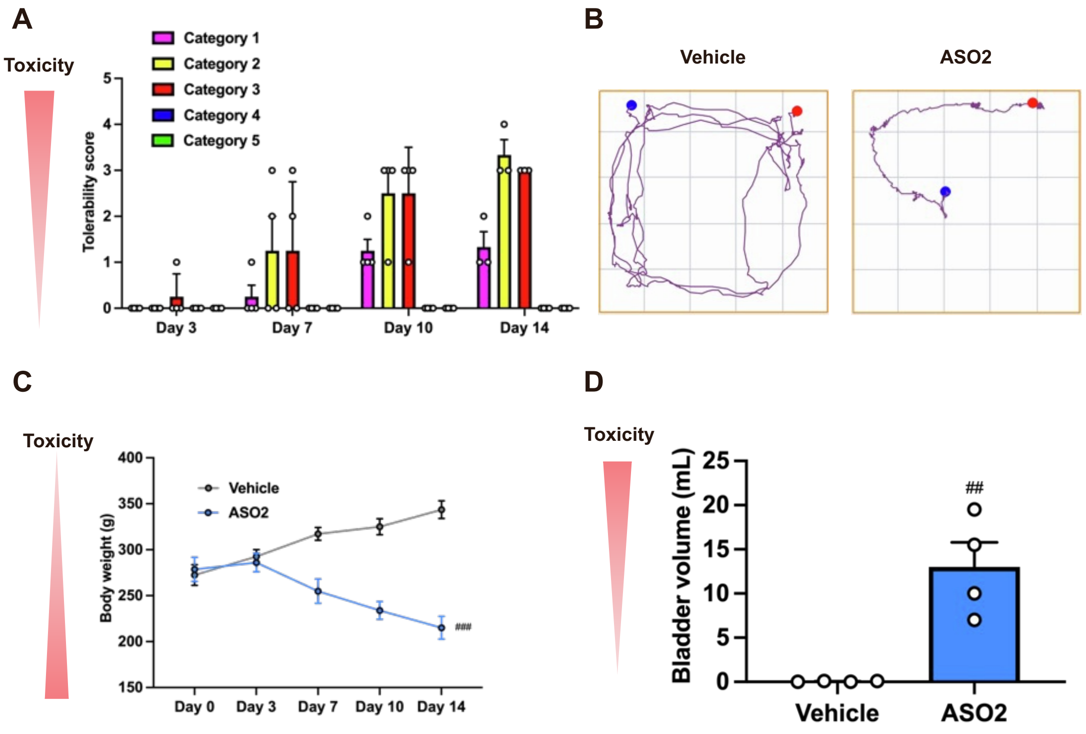

Figure S6. Late-onset neurotoxicity in rats after ICV-injection of ASO2.

Nine-week-old male Slc:SD rats injected intrathecally via spinal canal catheters with 190 nmol (1.0 mg) of ASO2. (**A**) Breakdown of tolerability score by category. (**B**) Representative track plots in open-field tests 14 days postinjection. (**C**) Changes in body weight. (**D**) Bladder volume at sacrifice (14 days postinjection). Data are presented as mean ± SEM. # indicates significance against vehicle.

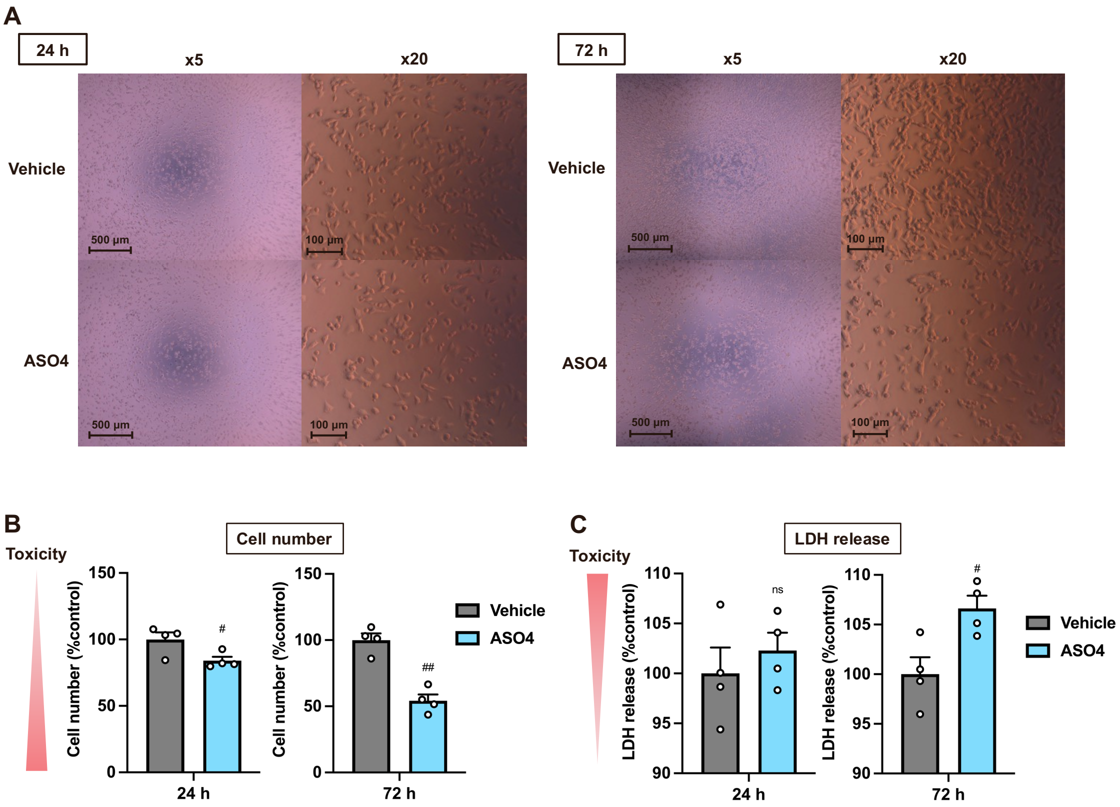

Figure S7. Neuronal cytotoxicity of ASO4 in BE(2)-M17 cells

BE(2)-M17 cells were transfected with 50 nM ASO4. (**A**) Representative microscopic images of cells 24 and 72 h after transfection. (**B**) cell number 24 h and 72 h post-transfection. (**C**) LDH release in medium 24 and 72 h post-transfection. Data in (**B, C**) are presented as mean ± SEM, as a percentage relative to the vehicle (n = 4 per group). Ns, not significant ($p > 0.05$). # indicates significance against vehicle.

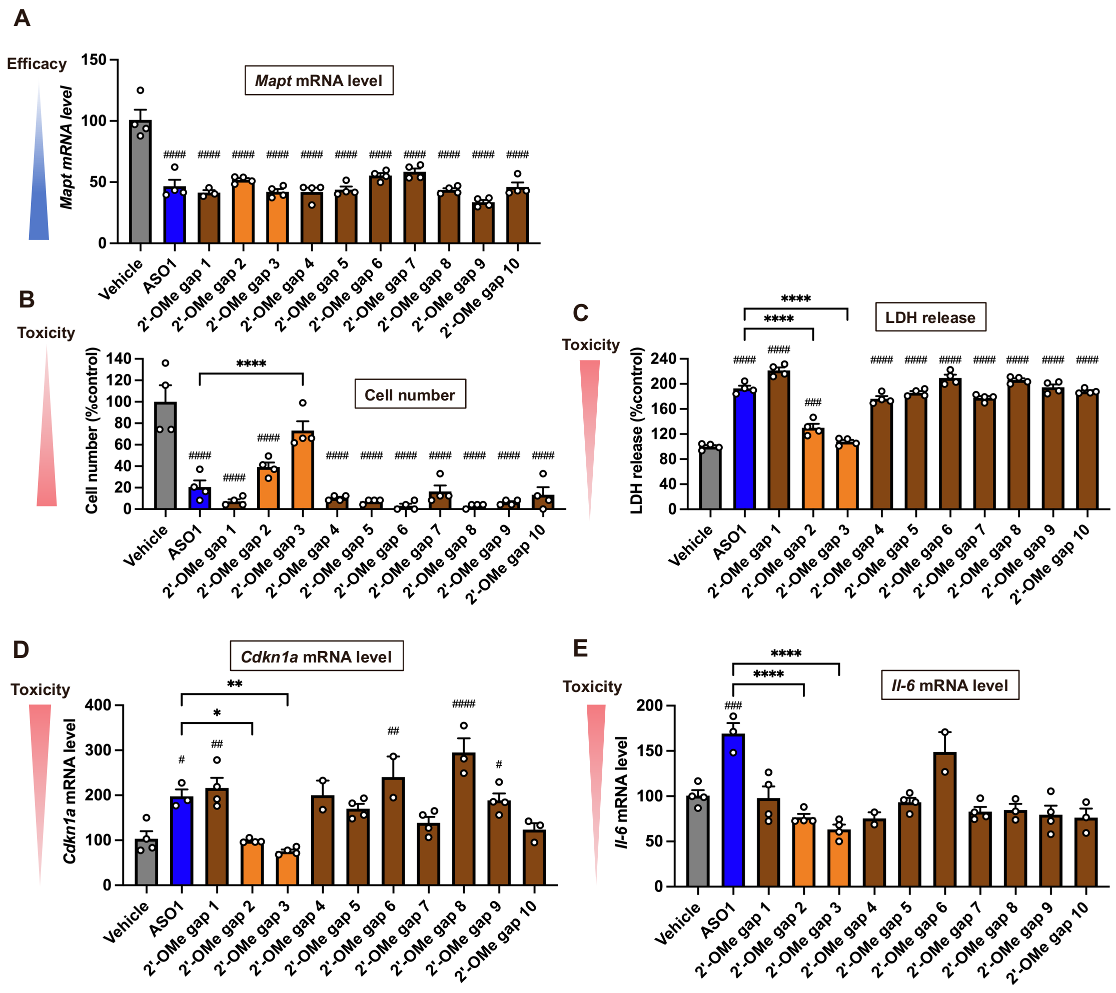

Figure S8. In vitro assessment of 2′-OMe gap walking in ASO1.

Neuro-2a cells were transfected with 4 nM ASO1 (knockdown activity assessment) or 50 nM ASO1 (toxicity assessment), with or without 2′-OMe modification at each gap position. Target *Mapt* mRNA levels (**A**), cell number (**B**). LDH release (**C**), *Cdkn1a* mRNA levels (**D**), and *Il-6* mRNA levels (**E**) 72 h post-transfection are presented as mean ± SEM, as a percentage relative to the vehicle (n = 2 per group for *Il-6* mRNA of ASO1 with 2′-OMe at gap position 4; n = 4 per group for the others). The highlighted sequences with 2′-OMe modifications show an increase in a cell number and a decrease in LDH release compared to ASO1. # and * indicates significance against vehicle and ASO1, respectively.

A

Sequence ASO4	Modification	T_m and ΔT_m (℃)	*SNCA* mRNA level (%control)	Cell number (%control)	LDH release (%control)	*CDKN1A* mRNA level (%control)
CCATTTATATACAAACACA	Parent	57	40	53	113	231
CCATTTATATACAAACACA	2′-OMe gap 1	+1	62 (+22)	49 (-4)	111 (-2)	293 (+62)
CCATTTATATACAAACACA	2′-OMe gap 2	+0	67 (+27)	61 (+8)	106 (-7)	368 (+137)
CCATTTATATACAAACACA	2′-OMe gap 3	+1	59 (+19)	51 (-2)	112 (-1)	210 (-21)
CCATTTATATACAAACACA	2′-OMe gap 4	-1	63 (+23)	66 (+13)	108 (-5)	146 (-85)
CCATTTATATACAAACACA	2′-OMe gap 5	+0	66 (+26)	54 (+1)	113 (+0)	93 (-138)
CCATTTATATACAAACACA	2′-OMe gap 6	-2	66 (+26)	60 (+7)	113 (+0)	41 (-190)

Sequence ASO4	Modification	T_m and ΔT_m (℃)	*SNCA* mRNA level (%control)	Cell number (%control)	LDH release (%control)	*CDKN1A* mRNA level (%control)
CCATTTATATACAAACACA	Parent	57	39	58	107	409
CCATTTATATACAAACACA	2′-OMe gap 7	+0	60 (+21)	48 (-10)	103 (-4)	386 (-23)
CCATTTATATACAAACACA	2′-OMe gap 8	-1	58 (+19)	63 (+5)	107 (+0)	391 (-18)
CCATTTATATACAAACACA	2′-OMe gap 9	+0	54 (+15)	50 (-8)	110 (+3)	269 (-140)
CCATTTATATACAAACACA	2′-OMe gap 10	-1	46 (+7)	52 (-6)	112 (+5)	417 (+8)
CCATTTATATACAAACACA	2′-OMe gap 11	+1	41 (+2)	47 (-11)	112 (+5)	384 (-25)
CCATTTATATACAAACACA	2′-OMe gap 12	-1	51 (+12)	48 (-10)	114 (+7)	365 (-44)

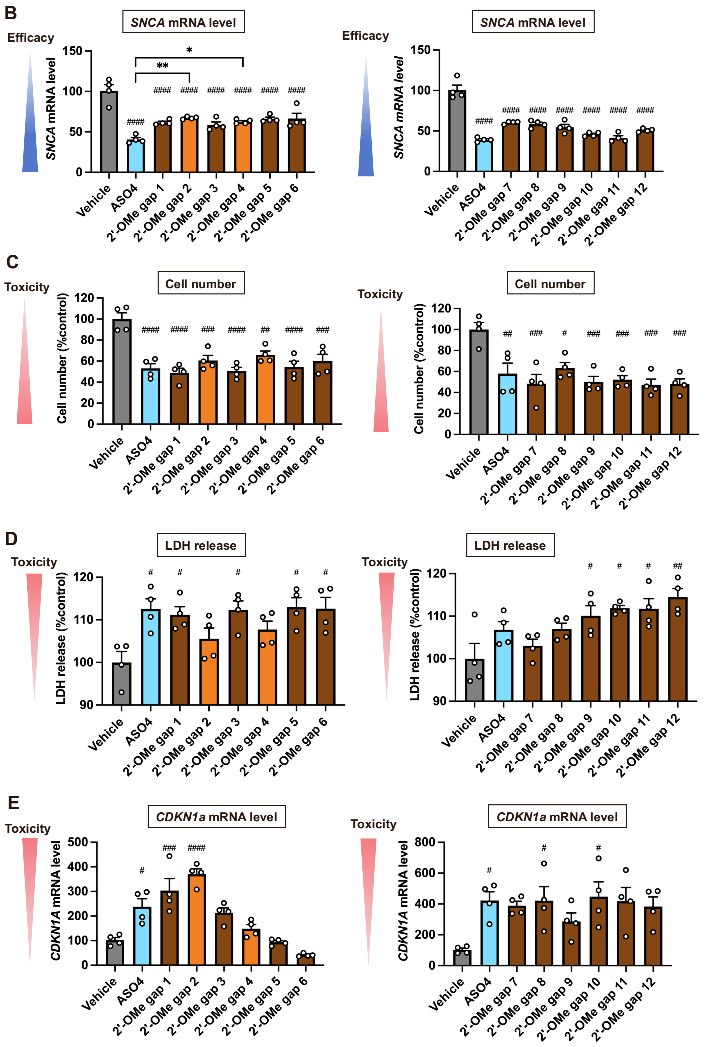

Figure S9. In vitro assessment of 2′-OMe gap walking in ASO4.

(**A**) BE(2)-M17 cells were transfected with 1 nM ASO4 (knockdown activity assessment) or 50 nM ASO4 (toxicity assessment), with or without 2′-OMe modification at each gap position. Blue letters indicate LNA, whereas blue "C" indicates LNA with 5-methylcytosine. Black letters indicate DNA and red letters indicate 2′-OMe modified DNA. ΔT_m indicates the T_m of the sequence minus the T_m of the parent ASO. Target mRNA levels, cell number, LDH release, and *CDKN1A* mRNA levels 72 h post-transfection are shown as the mean percentage relative to vehicle (n = 4 per group). The highlighted sequences with 2′-OMe modifications show an increase in cell number and a decrease in LDH release compared to the parent ASOs. (**B-E**) Dot plots for (**A**): Target *SNCA* mRNA levels (**B**), cell number (**C**). LDH release (**D**), and *CDKN1A* mRNA levels (**E**) 72 h post-transfection. Data are presented as mean ± SEM, as a percentage relative to the vehicle. The highlighted sequences with 2′-OMe modifications show an increase in cell number and a decrease in LDH release compared to ASO4. # and * indicates significance against vehicle and ASO4, respectively.

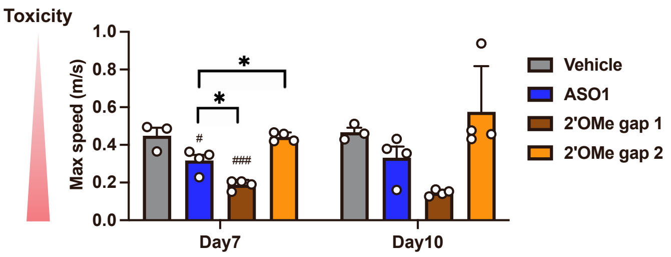

Figure S10. 2′-OMe at gap position 2 reduces late-onset neurotoxicity of ASO1 via ICV-injection.

Seven-week-old female ICR mice (n = 3 per group for vehicle, and n = 4 per group for all other groups) were injected intracerebroventricularly with 38.4 nmol of ASO1 and ASO1 modified with 2′-OMe at gap position 1 or 2. Maximum speed in open-field tests at the indicated time points. Data are presented as mean ± SEM. # and * indicates significance against vehicle and ASO1, respectively.

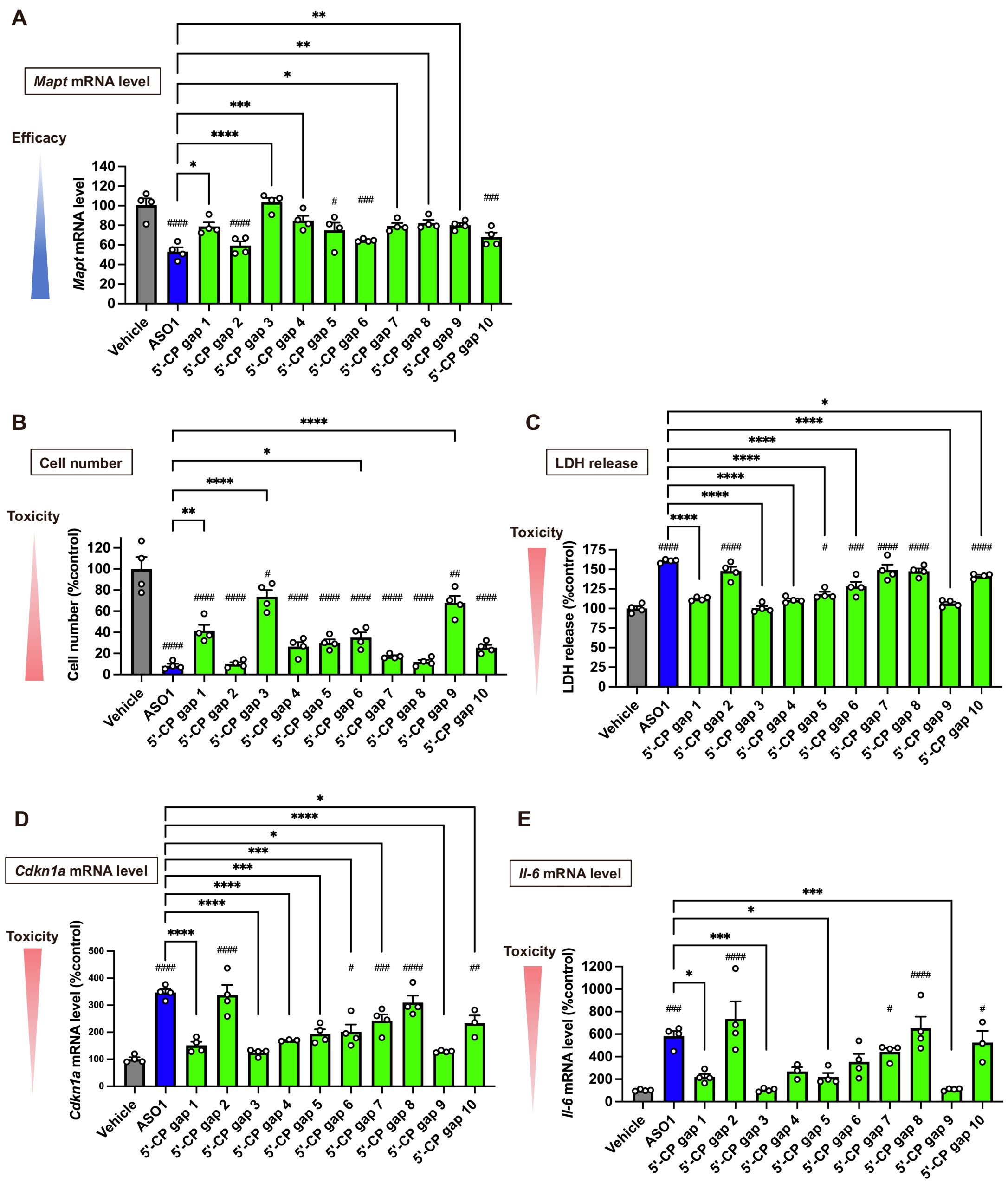

Figure S11. In vitro assessment of 5′-CP gap walking in ASO1.

Neuro-2a cells were transfected with 4 nM ASO1 (knockdown activity assessment) or 50 nM ASO1 (toxicity assessment), with or without 5′-CP modification at each gap position. Target *Mapt* mRNA levels (**A**), cell number (**B**). LDH release (**C**), *Cdkn1a* mRNA levels (**D**), and *Il-6* mRNA levels (**E**) 72 h post-transfection are presented as mean ± SEM, as a percentage relative to the vehicle (n = 3 per group for *Cdkn1a* and *Il-6* mRNA of ASO1 with 5′-CP at gap position 4 and 10; n = 4 per group for others). # and * indicates significance against vehicle and ASO1, respectively.

A

Sequence ASO4	Modification	T_m and ΔT_m (℃)	*SNCA* mRNA level (%control)	Cell number (%control)	LDH release (%control)	*CDKN1A* mRNA level (%control)
CCATTTATATACAAACACA	Parent	57	35	52	106	346
CCATTTATATACAAACACA	5′-CP gap 1	-2	65 (+30)	70 (+18)	100 (-6)	182 (-164)
CCATTTATATACAAACACA	5′-CP gap 2	-2	63 (+28)	64 (+12)	100 (-6)	207 (+139)
CCATTTATATACAAACACA	5′-CP gap 3	-2	58 (+23)	58 (+6)	100 (-6)	235 (-111)
CCATTTATATACAAACACA	5′-CP gap 4	-1	39 (+4)	44 (-8)	103 (-3)	337 (-9)
CCATTTATATACAAACACA	5′-CP gap 5	-2	47 (+12)	46 (-6)	103 (-3)	240 (-106)
CCATTTATATACAAACACA	5′-CP gap 6	-1	43 (+8)	30 (-22)	108 (+2)	325 (-21)

Sequence ASO4	Modification	T_m and ΔT_m (℃)	*SNCA* mRNA level (%control)	Cell number (%control)	LDH release (%control)	*CDKN1A* mRNA level (%control)
CCATTTATATACAAACACA	Parent	57	31	59	105	217
CCATTTATATACAAACACA	5′-CP gap 7	-2	44 (+13)	77 (+18)	102 (-3)	157 (-60)
CCATTTATATACAAACACA	5′-CP gap 8	-2	43 (+12)	78 (+19)	100 (-5)	186 (-31)
CCATTTATATACAAACACA	5′-CP gap 9	-2	46 (+15)	79 (+20)	101 (-4)	134 (-83)
CCATTTATATACAAACACA	5′-CP gap 10	-3	49 (+18)	63 (+4)	106 (+1)	177 (-40)
CCATTTATATACAAACACA	5′-CP gap 11	-2	43 (+8)	47 (-12)	111 (+6)	297 (+80)
CCATTTATATACAAACACA	5′-CP gap 12	-2	47 (+16)	68 (+9)	104 (-1)	170 (-47)

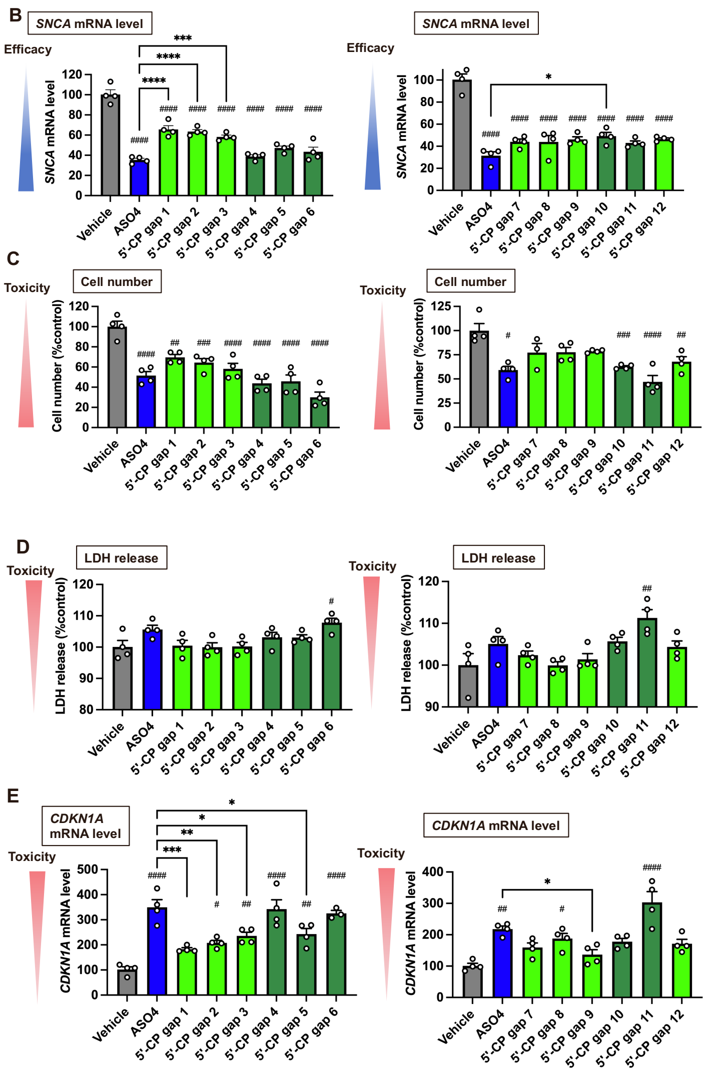

Figure S12. In vitro assessment of 5′-CP gap walking in ASO4.

(**A**) BE(2)-M17 cells were transfected with 1 or 50 nM ASO4, with or without 5′-CP at each gap position. Blue letters indicate LNA, whereas blue "C" indicates LNA with 5-methylcytosine. Black letters indicate DNA and green letters indicate 5′-CP-modified DNA. ΔT_m indicates the T_m of the sequence minus the T_m of the parent ASO. Target mRNA levels, cell number, LDH release, and *CDKN1A* mRNA levels 72 h post-transfection are shown as the mean percentage relative to vehicle (n = 4 per group). The highlighted sequences with 5′-CP modifications show an increase in cell number and a decrease in LDH release compared to the parent ASOs. (**B**-**E**) Dot plots for (**A**): Target *SNCA* mRNA levels (**B**), cell number (**C**). LDH release (**D**), and *CDKN1A* mRNA levels (**E**) 72 h post-transfection. Data are presented as mean ± SEM, as a percentage relative to the vehicle. The highlighted sequences with 5′-CP modifications show an increase in cell number and a decrease in LDH release compared to ASO4. # and * indicates significance against vehicle and ASO4, respectively.

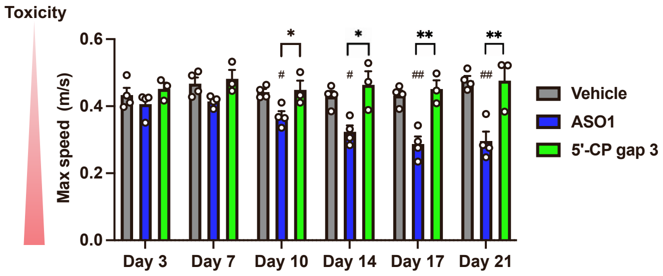

Figure S13. 5′-CP at gap position 3 mitigates late-onset neurotoxicity of ASO1 via ICV-injection.

Seven-week-old female ICR mice (n = 3 per group for ASO1 with 5′-CP at gap position 3, n= 4 per group for other groups) were injected intracerebroventricularly with 38.4 nmol of ASO1, with or without 5′-CP at gap position 3. Maximum speed in open-field tests were recorded on the indicated days postinjection. Data are presented as mean ± SEM. # and * indicates significance against vehicle and ASO1, respectively.

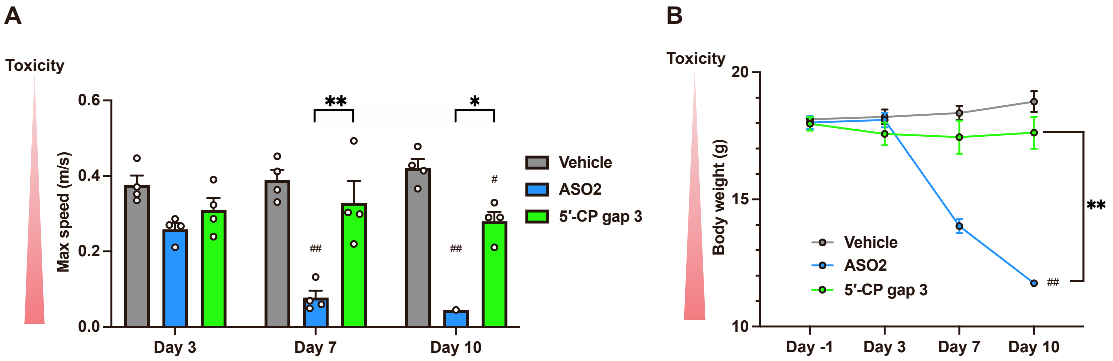

Figure S14. 5′-CP at gap position 3 mitigates late-onset neurotoxicity of ASO2 via ICV-injection.

Seven-week-old female C57BL/6 mice (n = 4 per group) were injected intracerebroventricularly with 19.0 nmol of ASO2, with or without 5′-CP modification at gap position 3. Mice were sacrificed 11 days postinjection. (**A**) Maximum speed in open-field tests were recorded on the indicated days postinjection. (**B**) Changes in body weight over time. Data are presented as mean ± SEM. # and * indicates significance against vehicle and ASO2, respectively.

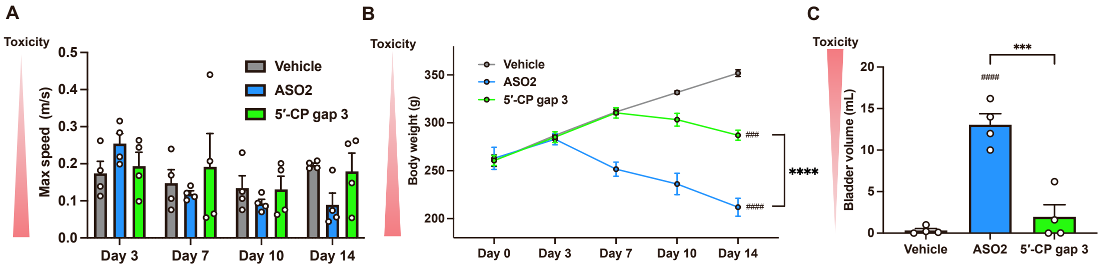

Figure S15. 5′-CP at gap position 3 mitigates late-onset neurotoxicity of ASO2 via IT-injection in rats.

Nine-week-old male Slc:SD rats (n = 4 per group) were injected intrathecally via spinal canal catheters with 190 nmol of ASO2, with or without 5′-CP modification at gap position 3. Rats were sacrificed 14 days postinjection. (**A**) Maximum speed in open-field tests were documented on the indicated days postinjection. (**B**) Changes in Body weight over time. (**C**) Bladder volume at sacrifice. Data are presented as mean ± SEM. # and * indicates significance against vehicle and ASO2, respectively.

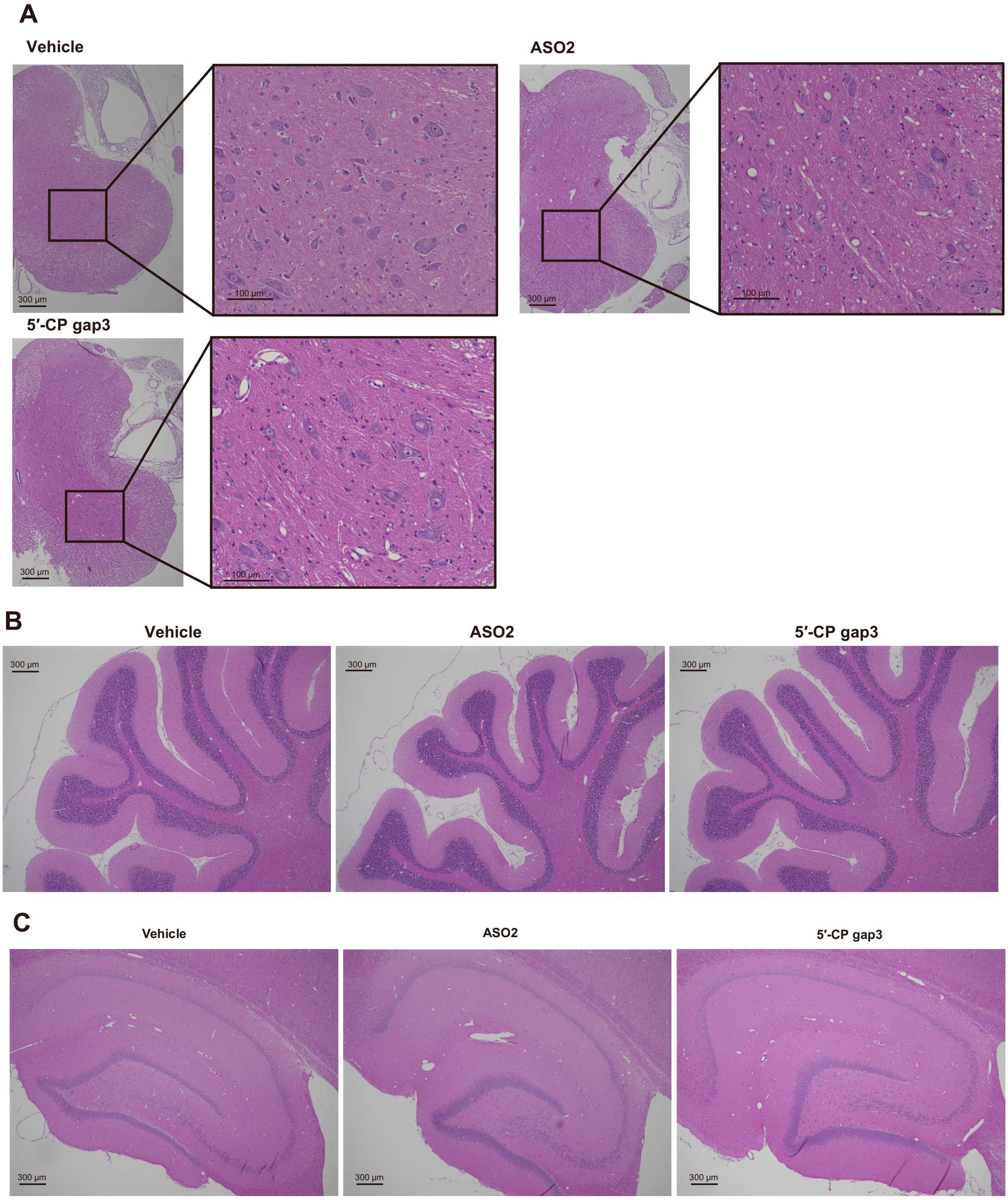

Figure S16. Representative H&E-stained images of rat lumbar cord (A), cerebellum (B), and hippocampus (C), 10 days after intrathecal injection of 190 nmol of ASO2, with or without 5′-CP modification at gap position 3.

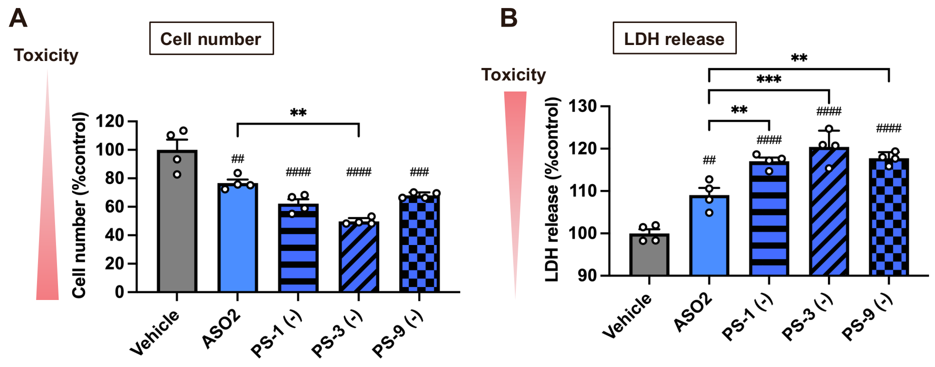

Figure S17. In vitro assessment of PS-1(-), PS-3(-), or PS-9(-) in the gap region in ASO2.

BE(2)-M17 cells were transfected with 20 nM ASO2 with or without replacement of a PS bond 5′-adjacent a DNA monomer at gap position 1, 3, or 9 with a PO bond (PS-1(-), PS-3(-), or PS-9(-)). Cell number (**A**) and LDH release (**B**) 72 h post-transfection are presented as mean ± SEM, as a percentage relative to the vehicle (n = 4 per group). # and * indicates significance against vehicle and ASO2, respectively.

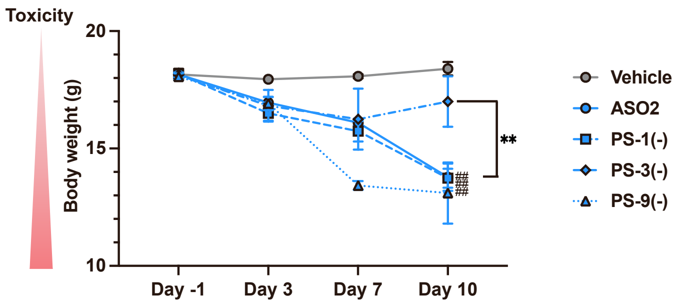

Figure S18. Changes in body weight in mice after ICV-injection of ASO2 with PS-1(-), PS-3(-), and PS-3(-).

Seven-week-old female C57BL/6 mice (n = 4 per group for vehicle and the parent ASO2; n=5 per group for the others) were injected intracerebroventricularly with 19.0 nmol of ASO2 or ASO2 with PS-1(-), PS-3(-), or PS-9(-). Toxicity was assessed on the indicated days, and mice were sacrificed 10 days postinjection. ** indicates a p-value ($p \leq 0.01$) between parent ASO2 and ASO2 PS-3(-). Data are presented as mean ± SEM. # indicates significance against vehicle.

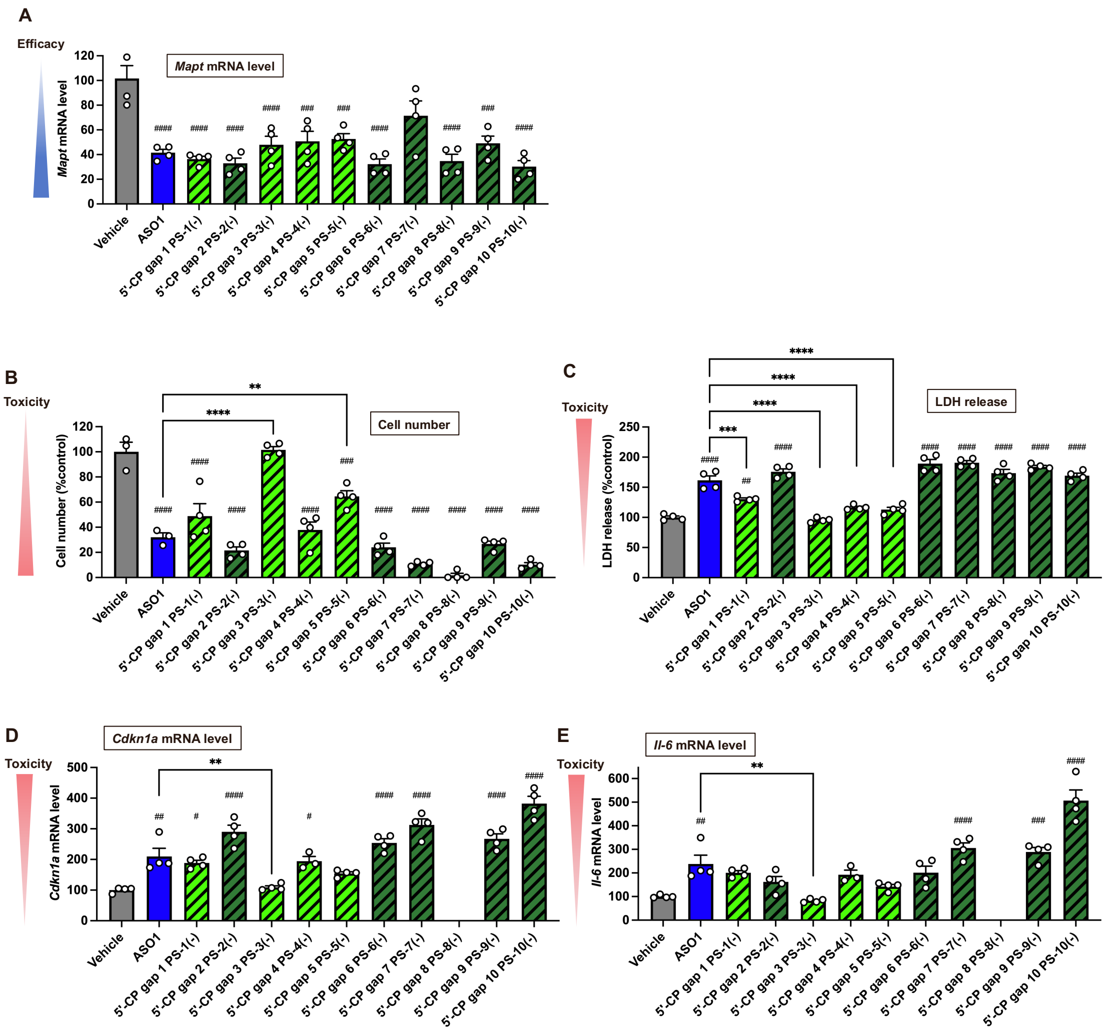

Figure S19. In vitro assessment of 5′-CP with PO-replacement gap walking in ASO1.

Neuro-2a cells were transfected with 4 nM ASO1 (knockdown activity assessment) or 50 nM ASO1 (toxicity assessment), with or without replacement of 5′-CP modification and the 5′-adjacent PS-bond with PO-bond at each gap position. Target *Mapt* mRNA levels (**A**), cell number (**B**). LDH release (**C**), *Cdkn1a* mRNA levels (**D**), and *Il-6* mRNA levels (**E**) 72 h post-transfection are presented as mean ± SEM, as a percentage relative to the vehicle. The highlighted sequences with 5′-CP modifications show an increase in a cell number and a decrease in LDH release compared to ASO1. For cell number, n = 3 per group in vehicle and parent ASO1; n = 4 per group for the others. *Cdkn1a* and *Il-6* mRNA levels for ASO1 with 5′-CP at gap position 8 PS-8(-) were unmeasurable due to insufficient RNA retraction with severe cytotoxicity. # and * indicates significance against vehicle and ASO1, respectively.

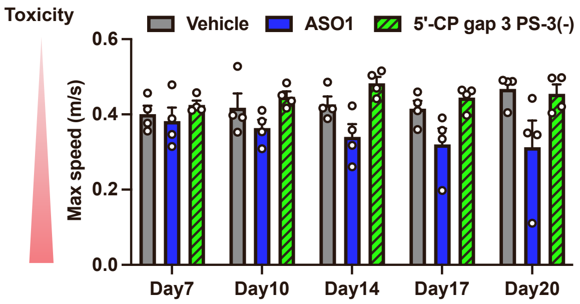

Figure S20. 5′-CP at gap position 3 with PS-3(-) mitigate late-onset neurotoxicity of ASO1 via ICV-injection.

Seven-week-old female ICR mice (n = 4 per group) were injected intracerebroventricularly with 38.4 nmol of ASO1 and ASO1 with 5′-CP at gap position 3 with PS-3(-). Toxicity was assessed on the indicated days, and mice were sacrificed 21 days postinjection. Maximum speed in open-field tests were documented on the indicated days postinjection. Data are presented as mean ± SEM.

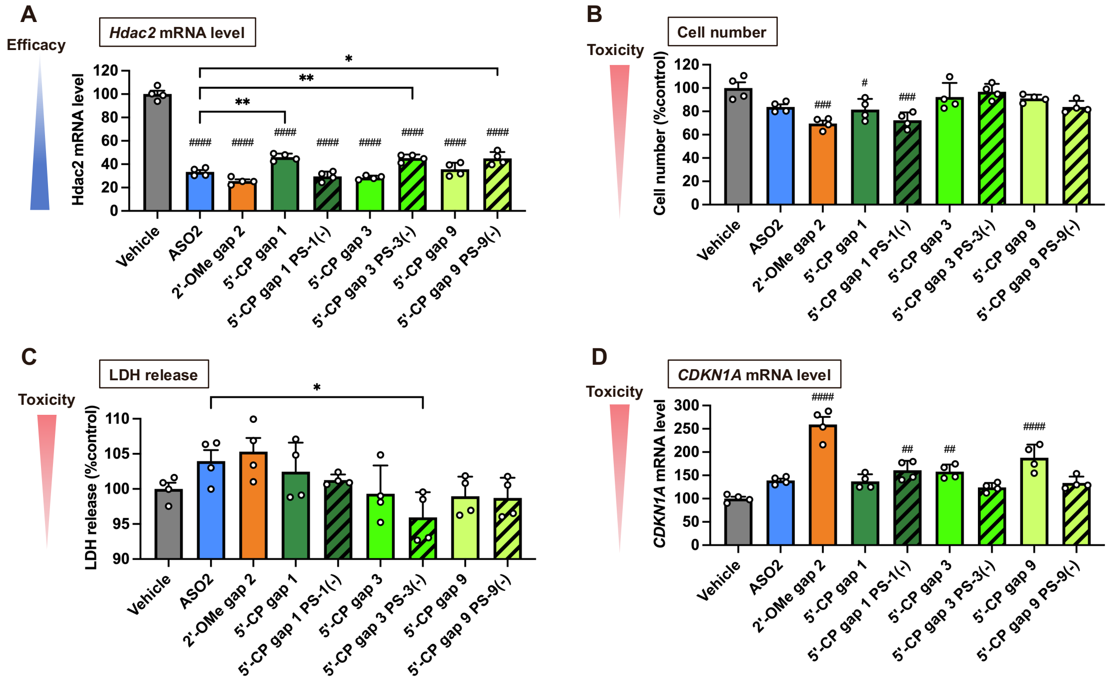

Figure S21. In vitro assessment of 2′-OMe, 5′-CP, and 5′-CP with PO-replacement in the gap region in ASO2.

Neuro-2a cells were transfected with 2 nM ASO (knockdown activity assessment) (**A**) and BE(2)-M17 cells were transfected with 20 nM ASO (toxicity assessment) (**B-D**), using the following ASOs: ASO2, ASO2 with 2′-OMe at gap position 2, and ASO2 with 5′-CP at gap position 1, 3, or 9 with or without PO-replacement. Target *Hdac2* mRNA levels (**A**), cell number (**B**). LDH release (**C**), and *CDKN1A* mRNA levels (**D**) 72 h post-transfection are presented as mean ± SEM, as a percentage relative to the vehicle (n = 4 per group). # and * indicates significance against vehicle and ASO2, respectively.

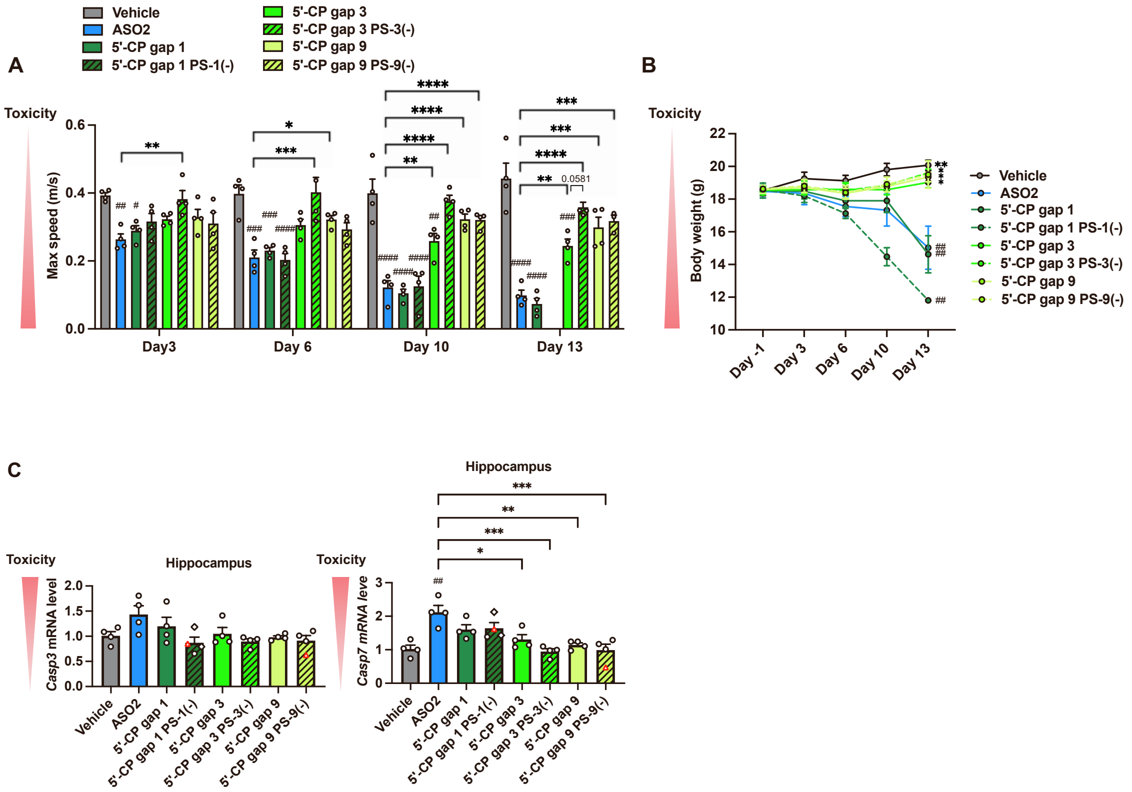

Figure S22. In vivo assessment of ASO2 with 5′-CP at gap positions 1, 3, or 9, with or without PO-replacement via ICV-injection.

Seven-week-old female C57BL/6 mice (n = 4 per group) were injected intracerebroventricularly with 19.0 nmol of the parent ASO2 and ASO2 with 5′-CP at gap positions 1, 3, or 9, with or without PO-replacement. Toxicity was assessed on the indicated days, and mice were sacrificed 14 days postinjection. (**A**) Maximum speed in open-field tests were recorded on the indicated days postinjection. (**B**) Changes in body weight postinjection. (**C**) *Caspase-3* (*Casp3*) and *Caspase-7* (*Casp7*) mRNA levels in the left hippocampus were measured 14 days postinjection. Symbols indicate collection days: rounds (day 14), rhombus (day 10), and red triangles (day 13, post-mortem). Data are presented as mean ± SEM. # and * indicates significance against vehicle and ASO2, respectively.

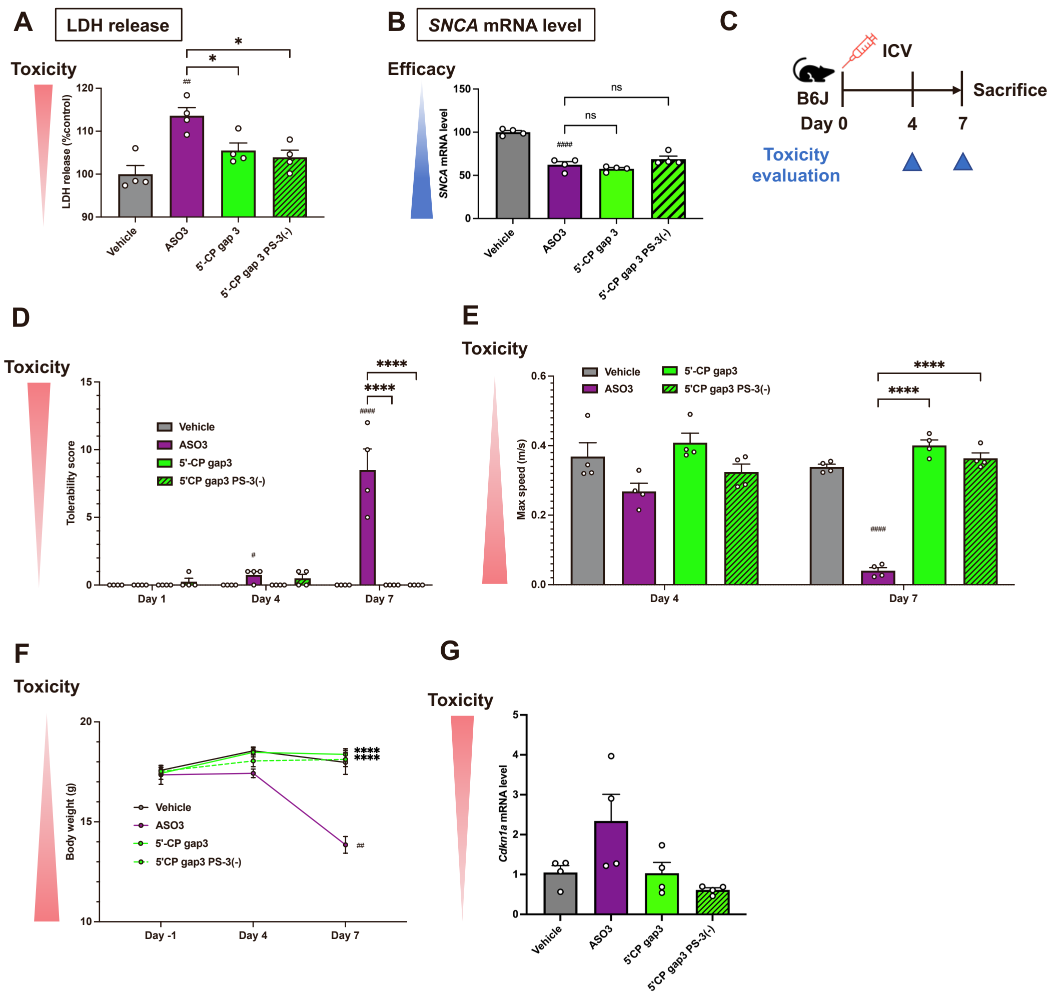

Figure S23. In vitro and in vivo assessment of ASO3 with 5′-CP at gap positions 3 with or without PO-replacement.

(**A, B**) BE(2)-M17 cells were transfected with 50 nM ASO3 (**A**) or 0.4 nM ASO3 (**B**), with 5′-CP at gap positions 3 or 5′-CP at gap positions 3 with PS-3(-). LDH release (**A**) and target mRNA levels (**B**) 72 h post-transfection are shown as mean ± SEM, as a percentage relative to the vehicle. (**C-G**) Seven-week-old female C57BL/6 mice (n = 4 per group) were injected intracerebroventricularly with 15.2 nmol of the parent ASO3 and ASO3 with 5′-CP at gap positions 3 with or without PO-replacement. Tolerability scores (**D**), maximum speed (**E**), change in body weight (**F**) and *Cdkn1a* mRNA levels in the left hippocampus (**G**) were measured. Data are presented as mean ± SEM. $^{\#}p \leq 0.05$, $^{\#\#}p \leq 0.01$, and $^{\#\#\#\#}p \leq 0.0001$; data were analyzed using Student's two-tailed t-tests with vehicle. *$p \leq 0.05$ and ****$p \leq 0.0001$; data were analyzed using one-way ANOVA, followed by Tukey's post hoc tests with ASO3. Ns, not significant ($p > 0.05$).

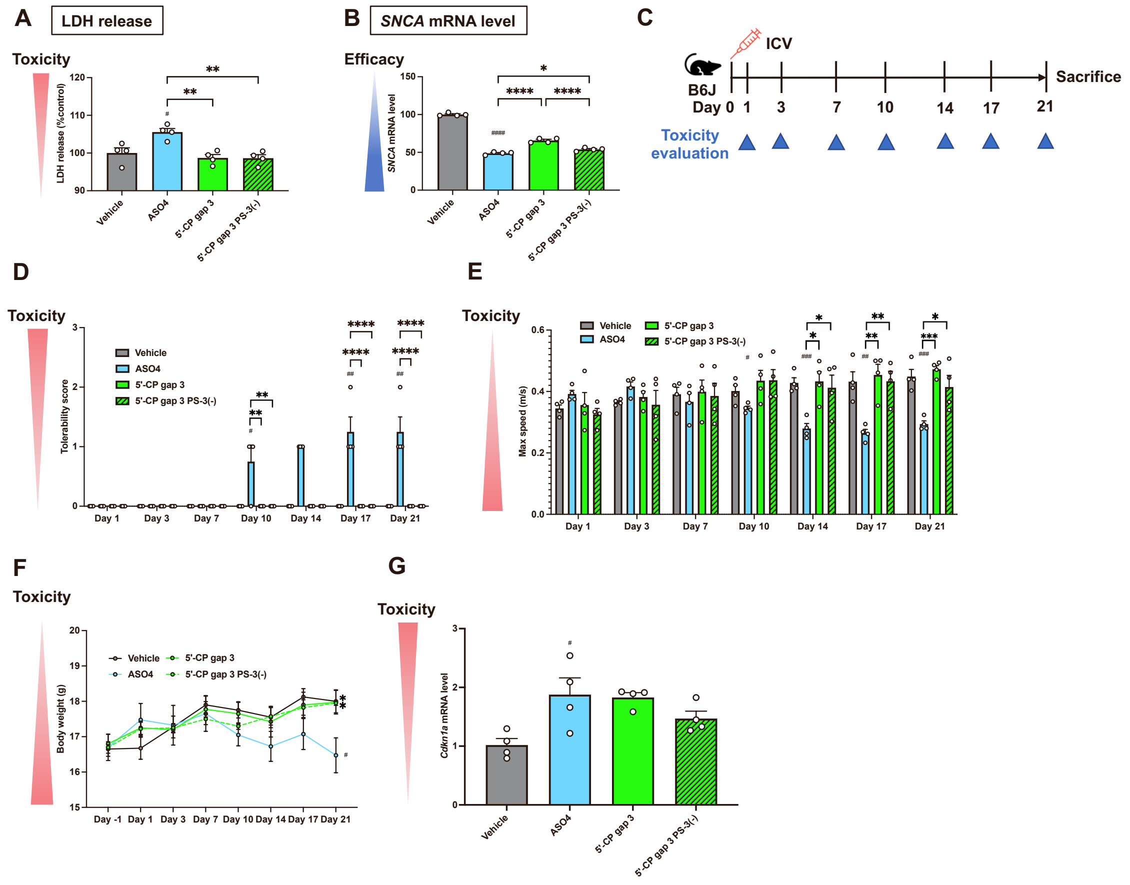

Figure S24. In vitro and in vivo assessment of ASO4 with 5′-CP at gap positions 3 with or without PO-replacement.

(**A, B**) BE(2)-M17 cells were transfected with 50 nM ASO4 (**A**) or 1 nM ASO4 (**B**), with 5′-CP at gap positions 3 or 5′-CP at gap positions 3 with PS-3(-). LDH release (**A**) and target mRNA levels (**B**) 72 h post-transfection are shown as mean ± SEM, as a percentage relative to the vehicle. (**C-G**) Seven-week-old female C57BL/6 mice (n = 4 per group) were injected intracerebroventricularly with 39.9 nmol of the parent ASO4 and ASO4 with 5′-CP at gap positions 3 with or without PO-replacement. Tolerability scores (**D**), maximum speed (**E**), change in body weight (**F**) and *Cdkn1a* mRNA levels in the left hippocampus (**G**) were measured. Data are presented as mean ± SEM. $^{\#,}p \leq 0.05$, $^{\#\#,}p \leq 0.01$, $^{\#\#\#}p \leq 0.001$, and $^{\#\#\#\#}p \leq 0.0001$; data were analyzed using Student's two-tailed t-tests with vehicle. *$p \leq 0.05$, **p ≤ 0.01, ***p ≤ 0.001, and ****p ≤ 0.0001; data were analyzed using one-way ANOVA, followed by Tukey's post hoc tests with ASO4, or as indicated.

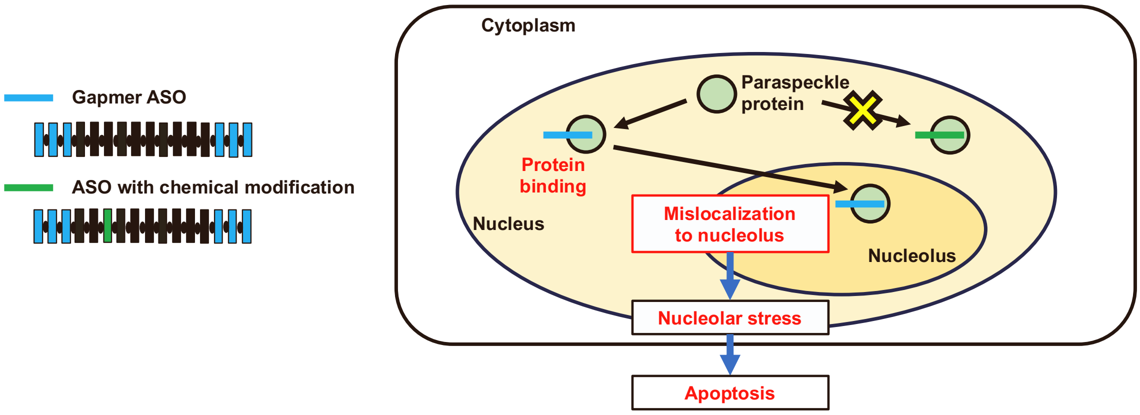

Figure S25. Schematic of PS-ASO-induced cytotoxicity and its prevention by chemical modification.

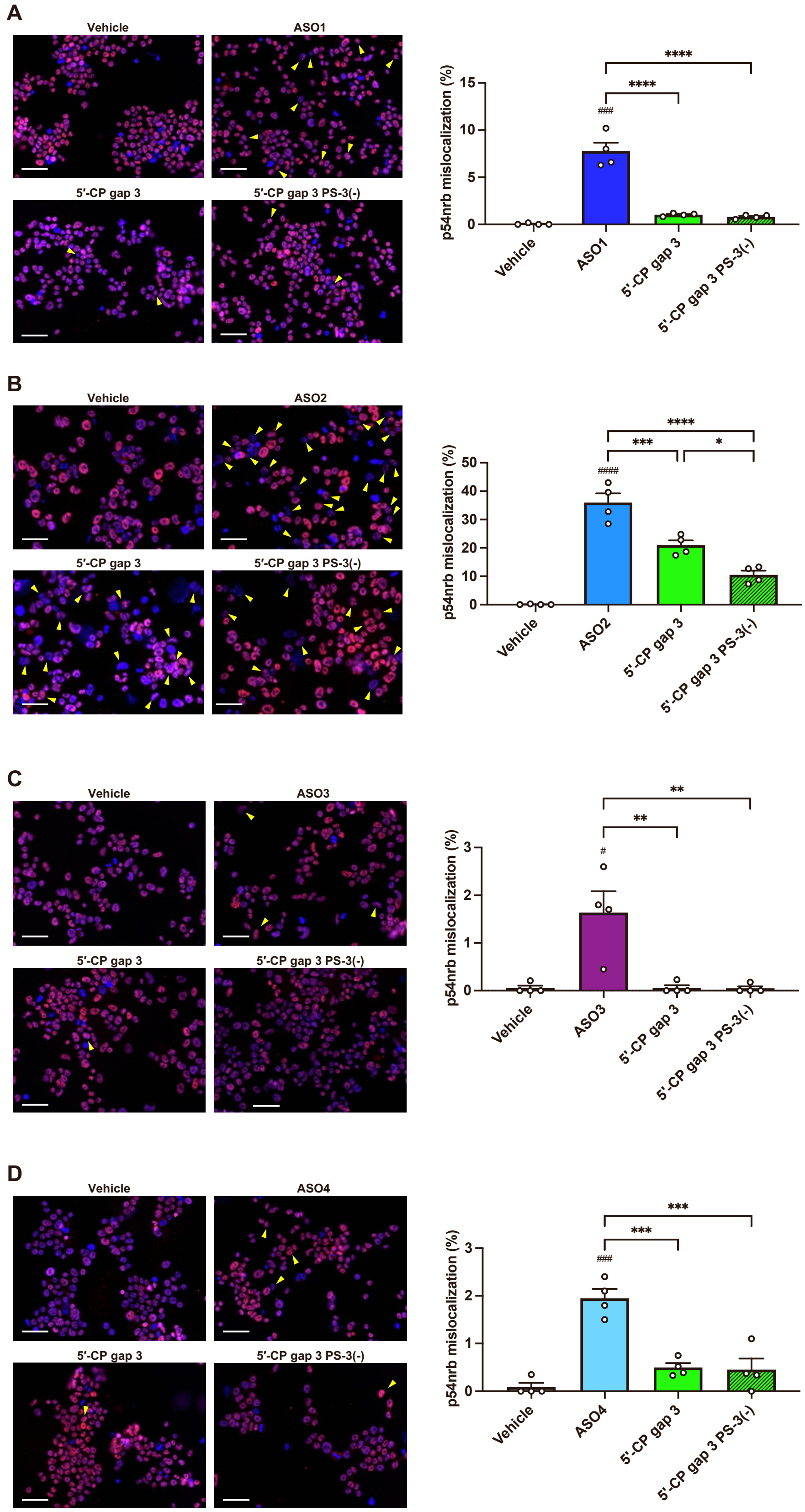
A
Vehicle
ASO1
5′-CP gap 3
5′-CP gap 3 PS-3(-)
p54nrb mislocalization (%)
****
****
###
Vehicle
ASO1
5′-CP gap 3
5′-CP gap 3 PS-3(-)
B
Vehicle
ASO2
5′-CP gap 3
5′-CP gap 3 PS-3(-)
p54nrb mislocalization (%)
****
***
*
####
Vehicle
ASO2
5′-CP gap 3
5′-CP gap 3 PS-3(-)
C
Vehicle
ASO3
5′-CP gap 3
5′-CP gap 3 PS-3(-)
p54nrb mislocalization (%)
**
**
#
Vehicle
ASO3
5′-CP gap 3
5′-CP gap 3 PS-3(-)
D
Vehicle
ASO4
5′-CP gap 3
5′-CP gap 3 PS-3(-)
p54nrb mislocalization (%)
***
***
###
Vehicle
ASO4
5′-CP gap 3
5′-CP gap 3 PS-3(-)

Figure S26. Immunofluorescence study of p54nrb in BE(2)-M17 cells transfected with ASO1-4 modified with 5′-CP at gap position 3, with or without PO-replacement.

BE(2)-M17 cells were transfected for 3 h with 200 nM of ASOs modified with 5′-CP at gap position 3, with or without PO-replacement. Representative microscopic immunostaining images of p54nrb and percentage of p54nrb mislocalization to nucleoli are shown for each ASO: (**A**) ASO1, (**B**) ASO2, (**C**) ASO3, and (**D**) ASO4. Data are presented as mean ± SEM of different microscopic images within the same well (n = 4 per group). $^{\#}p \leq 0.05$, $^{\#\#\#}p \leq 0.001$, and $^{\#\#\#\#}p \leq 0.0001$; data were analyzed using Student's two-tailed t-tests with vehicle. $^{*}p \leq 0.05$, $^{**}p \leq 0.01$, $^{***}p \leq 0.001$, and $^{****}p \leq 0.0001$; data were analyzed using one-way ANOVA, followed by Tukey's post hoc tests. Scale bar, 50 µm.

Videos S1-S3: Videos of mice from the experiment shown in Figures 3E-3H (n=4 per group), injected with either vehicle (Video S1), ASO2 (Video S2), or ASO2 with 2′-OMe modification at gap position 2 (Video S3), recorded 6 days postinjection.

Videos S4-S6: Representative videos of a rat from the experiment shown in Figures 4L and 4M (n=4 per group), injected with either vehicle (Video S4), ASO2 (Video S5), or ASO2 with 5′-CP modification at gap position 3 (Video S6), recorded 14 days postinjection.

Videos S7-S13: Representative videos of two mouse from the experiment shown in Figures 6H-6K (n=4 per group), injected with ASO2 (Video S7), ASO2 with 5′-CP at gap position 1 (Video S8), ASO2 with 5′-CP at gap position 1 with PS-1(-) (Video S9), ASO2 with 5′-CP at gap position 3 (Video S10), ASO2 with 5′-CP at gap position 3 with PS-3(-) (Video S11), ASO2 with 5′-CP at gap position 9 (Video S12), ASO2 with 5′-CP at gap position 9 with PS-9(-) (Video S13), recorded 10 days postinjection.
